# 3D Structures of Plant Phytochrome A as Pr and Pfr From Solid-State NMR: Implications for Molecular Function

**DOI:** 10.3389/fpls.2018.00498

**Published:** 2018-04-24

**Authors:** Chen Song, Maria Andrea Mroginski, Christina Lang, Jakub Kopycki, Wolfgang Gärtner, Jörg Matysik, Jon Hughes

**Affiliations:** ^1^Institut für Analytische Chemie, Universität Leipzig, Leipzig, Germany; ^2^Leids Instituut voor Chemisch Onderzoek, Universiteit Leiden, Leiden, Netherlands; ^3^Institut für Chemie, Technische Universität Berlin, Berlin, Germany; ^4^Institut für Pflanzenphysiologie, Justus-Liebig-Universität, Giessen, Germany

**Keywords:** chromophore–protein interaction, photochromicity, QM/MM, protein refolding, signal transduction

## Abstract

We present structural information for oat phyA3 in the far-red-light-absorbing (Pfr) signaling state, to our knowledge the first three-dimensional (3D) information for a plant phytochrome as Pfr. Solid-state magic-angle spinning (MAS) NMR was used to detect interatomic contacts in the complete photosensory module [residues 1–595, including the NTE (N-terminal extension), PAS (*P*er/*A*rnt/*S*im), GAF (c*G*MP phosphodiesterase/*a*denylyl cyclase/*F*hlA) and PHY (*phy*tochrome-specific) domains but with the C-terminal PAS repeat and transmitter-like module deleted] auto-assembled *in vitro* with ^13^C- and ^15^N-labeled phycocyanobilin (PCB) chromophore. Thereafter, quantum mechanics/molecular mechanics (QM/MM) enabled us to refine 3D structural models constrained by the NMR data. We provide definitive atomic assignments for all carbon and nitrogen atoms of the chromophore, showing the Pfr chromophore geometry to be periplanar *ZZEssa* with the ***D***-ring in a *β*-facial disposition incompatible with many earlier notions regarding photoconversion yet supporting circular dichroism (CD) data. The Y268 side chain is shifted radically relative to published Pfr crystal structures in order to accommodate the *β*-facial ring ***D***. Our findings support a photoconversion sequence beginning with Pr photoactivation via an anticlockwise ***D***-ring *Za*→*Ea* photoflip followed by significant shifts at the coupling of ring ***A*** to the protein, a ***B***-ring propionate partner swap from R317 to R287, changes in the ***C***-ring propionate hydrogen-bonding network, breakage of the D272–R552 salt bridge accompanied by sheet-to-helix refolding of the tongue region stabilized by Y326–D272–S554 hydrogen bonding, and binding of the NTE to the hydrophobic side of ring ***A***. We discuss phyA photoconversion, including the possible roles of mesoscopic phase transitions and protonation dynamics in the chromophore pocket. We also discuss possible associations between structural changes and translocation and signaling processes within the cell.

## Introduction

Plant phytochromes mediate the largest environmentally determined developmental changes known in nature, including the induction of germination, de-etiolation and flowering, about 20% of all genes showing major transcriptional regulation by phytochrome. This is achieved by photoconversion of the physiologically inactive Pr state [*λ*_max_ in the red (R) region, ∼660 nm] to the Pfr signaling state [*λ*_max_ in the far-red (FR) region, ∼730 nm]. Although both represent singlet electronic ground states (S_0_), Pfr slowly reverts to Pr in darkness or can be photoconverted back to Pr by FR light. The situation is complicated significantly, however, by the existence of two functionally dominant phytochromes in plants, namely phyA and phyB, each with very different physiological properties and ecological roles, yet likely undergoing similar photochemical processes. Although plant phytochromes have been studied intensively for many years on account of their importance in natural ecosystems and agriculture, many fundamental aspects are still scarcely understood. As expected, the discovery of Cph1 phytochrome from the cyanobacterium *Synechocystis* 6803 (Hughes et al., 1997; Yeh et al., 1997) and subsequently BphPs (Davis et al., 1999) provided access to powerful biophysical methods, in particular X-ray crystallography, 3D structures of both Pr and Pfr *inter alia* providing clues regarding the mechanism of photoactivation and signaling. In particular, it seems that the remarkable tongue-like hairpin refolds radically upon photoconversion, perhaps leading to a shift of the PHY domain and the associated transmitter module (comprising DHp and CAT domains) which functions as a light-repressed HK (Anders et al., 2013; Takala et al., 2014). Although particularly Cph1 has close similarities to plant phytochromes, including the position of the chromophore attachment site in the GAF domain, differences between plant and cyanobacterial phytochromes should be kept in mind. In particular, (i) in Cph1 and other prokaryotic phytochromes Pr is the signaling state, whereas in plant phytochromes it is Pfr, (ii) the transmitter-like module of plant phytochromes resembles but is not a HK, (iii) even though other domains are involved, the light-signal from plant phytochromes arises from the sensory module itself (Matsushita et al., 2003; Qiu et al., 2017), and (iv) plant phytochromes alone include a tandem PAS-domain repeat between the N-terminal sensory and C-terminal transmitter-like modules. Consequently, the extent to which even Cph1 represents a valid model for plant phytochromes is limited. Nevertheless, on the basis of the Cph1 structure (PDB code 2VEA; Essen et al., 2008) the first crystal structure of a plant phytochrome fragment was recently solved (PDB code 4OUR; Burgie et al., 2014a), although at 3.4-Å resolution several important details are unclear, including the geometry of the bilin chromophore.

The present paper focuses on oat (*Avena sativa*) phyA, the material on which most of the pioneering phytochrome work was focused. Indeed, it was the first phytochrome to be purified intact (Vierstra and Quail, 1982) and spectrally functional (Litts et al., 1983; Vierstra and Quail, 1983), cloned (Hershey et al., 1984), sequenced (Hershey et al., 1985), and overexpressed in transgenic plants (Boylan and Quail, 1989; Keller et al., 1989). Moreover, type-A phytochromes confer the exquisite light sensitivity unique to higher plants and might thereby have been a crucial factor in their evolutionary success by providing improved physiological regulation of germination and de-etiolation. It is therefore of interest to understand its particular molecular characteristics and action mechanism.

Although spectroscopic methods do not rival crystallography in solving 3D structures of complete proteins, MAS NMR continues to gain in value for protein structural studies, as it unveils outstandingly precise molecular details without the need to crystalize the protein. MAS NMR has contributed to phytochrome research, Cph1 auto-assembled with *u*-[^13^C,^15^N]-PCB providing information on the little-known Pfr→Pr back-conversion route, confirming that the chromophore is fully protonated in both Pr and Pfr, and revealing the existence of at least two Pr sub-states, only one of which is represented by the crystal structure (Rohmer et al., 2008, 2010; Song et al., 2011a,b; Stöppler et al., 2016). We also showed that Pr is intrinsically less rigid than Pfr, implying a mesoscopic phase transition associated with photoconversion (Song et al., 2011a). Furthermore, our MAS NMR work on the oat phyA3 sensory module in the Pr state also provided the first structural information of any kind for a plant phytochrome, and revealed that here too Pr exists in at least two sub-states (Song et al., 2012). In this study we extend that work, presenting complete and unambiguous ^13^C and ^15^N chemical shift assignments for the chromophore and also tentative ^1^H assignments for the surrounding protein in both Pr and Pfr states. The latter was achieved with the help of 3D homology models optimized by NMR-restrained QM/MM calculations in which 198 atoms of the chromophore and its immediate environment were treated quantum mechanically. The Pr and Pfr models (deposited in Supplementary Data Sheet S1) provide insight into the photoconversion process.

## Results

### Chemical Shift Assignments of the Chromophore in Its Pfr State

**FIGURE 1:**
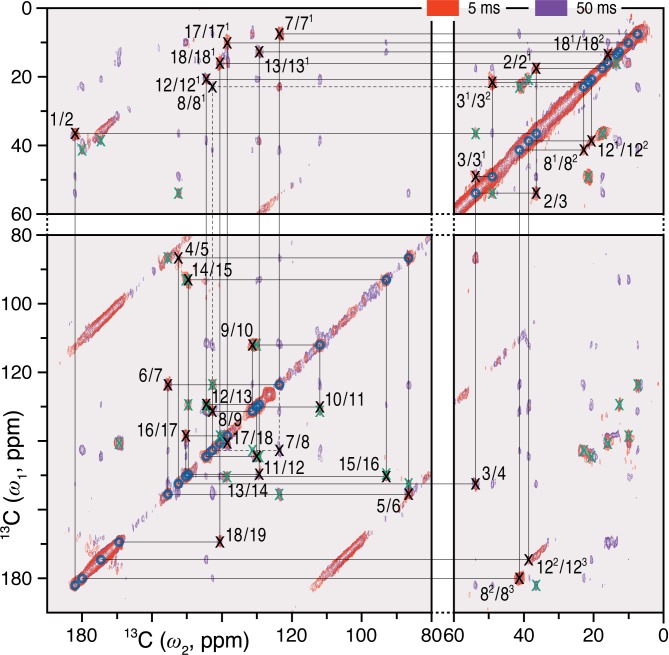
2D ^13^C–^13^C DARR spectra of the oat phyA3 *u*-[^13^C,^15^N]-PCB chromophore as Pfr with ^1^H mixing times of 5 (red) and 50 (purple) ms. The cross-peaks assigned to the directly bonded chromophore carbons are marked × in black and numbered (for PCB chromophore numbering, see **Figure 3**). Corresponding off-diagonal counterparts are marked × in green. The cross-peaks that are not fully resolved using the mixing time of 5 ms are indicated by dashes. See also Supplementary Figure S1.

Complete and unambiguous ^13^C and ^15^N assignments of the *u*-[^13^C,^15^N]-PCB chromophore as Pfr were derived from a series of 2D homo- and heteronuclear correlation experiments as for Pr (Song et al., 2012; see also Supplementary Results). ^13^C–^13^C dipolar correlation (DARR) experiments allowed the detection of direct and indirect ^13^C–^13^C correlations of the PCB chromophore within its binding pocket (**Figure 1** and Supplementary Figure S1). C4, C6, C18^1^ and C18^2^, previously unresolved for Pr (Rohmer et al., 2008), were assigned unambiguously, whereby assignment of the bilin carbon atoms in the Pfr state was completed (Supplementary Table S1). Neither the propionate side chains nor the ***A***-ring carbons showed the signal splitting observed in Pr (Song et al., 2012). The C11^…^C12^2…^C13^1…^C17^…^C18^2^ correlation network was well resolved in Pfr (Supplementary Figure S1), the 3.7–3.8 Å internuclear distances according to the QM/MM model corresponding to the maximum effective detection range using a 50 ms mixing time with our current sensitivity.

**FIGURE 2:**
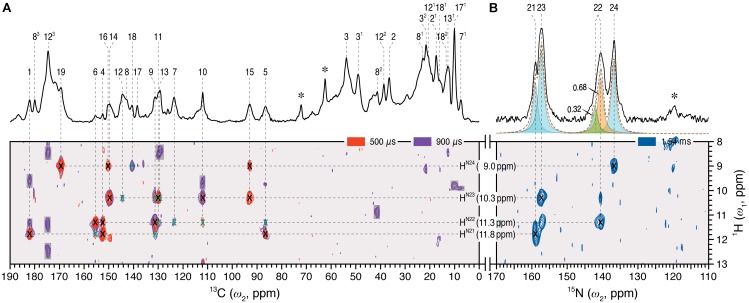
2D ^1^H–^13^C MELODI–HETCOR and ^1^H–^15^N HETCOR spectra of the oat phyA3 *u*-[^13^C,^15^N]-PCB chromophore as Pfr. **(A)** The MELODI–HETCOR spectrum acquired with an LG–CP contact time of 500 μs (red) reveals mostly intramolecular ^1^H contacts of the chromophore carbons (for example, ^1^H^N21 - N24^–^13^C^PCB^, marked × in black). Heteronuclear signals obtained with a mixing period of 900 μs (purple) fall into two categories: (i) Intramolecular ^1^H^N21 - N24^–^13^C^PCB^ correlations (marked × in black and green for fully and less resolved signals, respectively), and (ii) interfacial correlations between the chromophore and protein residues in its vicinity (shaded in gray). Intramolecular ^1^H^N21 - N24^–^13^C^PCB^ correlations are summarized in Supplementary Table S2. **(B)** The ^1^H–^15^N HETCOR spectrum was acquired for tracing direct ^1^H–^15^N connectivities. For all three correlation spectra, only the characteristic spectral region of *δ*^H^ = 8–13 ppm (*ω*_1_-dimension) is shown. *δ*^H^ and *δ*^C^/(*δ*^N^) values are indicated by the horizontal and vertical lines, respectively. The 1D ^13^C and ^15^N MAS spectra of the same protein as Pfr are shown with assignments as the external projections along the *ω*_2_-dimension above the spectra in **(A,B)**, respectively. Resonances marked with an asterisk indicate glycerol carbons and amide nitrogens of protein backbone originating from ^13^C and ^15^N in natural abundance.

Dipolar-filtered ^1^H–^13^C heteronuclear correlation (MELODI–HETCOR) then allowed ^1^H intramolecular contacts between the chromophore carbons and the retained NH (H^N21-N24^) and OH protons to be established, the spectrum with a cut-off distance of ∼2.8 Å (**Figure 2A**, red) revealing four clear pyrrolic ^1^H resonances. The NH proton resonating at 9.0 ppm was assigned to H^N24^ via its contacts with C19 (169.5 ppm), C16 (150.3 ppm), and C18 (140.7 ppm) at ring ***D***. These carbons showed no contacts to other NH protons even with a ∼3.5 Å cut-off distance (**Figure 2A**, purple; Supplementary Table S2). The NH proton at 11.8 ppm showed correlations to C1 and C4 of ring ***A*** as well as C5 and hence was assigned to the H^N21^ (**Figure 2A**, red). The integrated intensity ratio of the H^N21^ contacts to C1 and C4 is ∼45:55, implying that the two ***A***-ring carbons are roughly equidistant from this proton (van Rossum et al., 2002), supporting the assignment. Although the ^1^H signals corresponding to the nitrogens of rings ***B*** and ***C*** correlate with both C9 and C11, their different intensities allowed the assignment of H^N22^ and H^N23^ 11.3 and 10.3 ppm, respectively (in Pr the two protons were indistinguishable at 10.7 ppm; Supplementary Table S2). The assignment of all four NH protons is thereby complete. State-related *δ*^H^ changes (Δ*δ*^H^) were seen: -0.3, +0.6, -0.4 and +0.8 ppm as Pfr minus Pr for H^N21^ to H^N24^, respectively. The lack of correlations involving OH protons of the propionic side chains of rings ***B*** and ***C*** in the two MELODI–HETCOR spectra implies that both are deprotonated, as in earlier studies (Bhoo et al., 1997; Essen et al., 2008; Yang et al., 2008; Song et al., 2011b).

From the ^1^H data, ^1^H–^15^N HETCOR (**Figure 2B**) allowed us to assign the nitrogens themselves straightforwardly, with N21–N24 located at 159.0, 140.7, 157.3, and 136.9 ppm, respectively (Supplementary Table S1). The experimental *δ*^N^ values were closely matched by those computed using a QM/MM approach (*δ*^N21-N24^ = 165.3, 140.7, 154.1, and 138.0 ppm, respectively; see Supplementary Table S3). It should also be noted that the recent ^15^N DNP enhanced MAS NMR characterization of the Pr state in Cph1 finalized the assignment of the Pr resonances (Stöppler et al., 2016), in which the previous tentative assignments of N22 and N23 (Rohmer et al., 2008) were interchanged. This thus forms a solid basis on calculating the state-induced *δ*^N^ changes of the chromophore in oat phyA3 (see below).

### Changes in Chromophore ^13^C and ^15^N Chemical Shifts and Line-Widths in Pr and Pfr

**FIGURE 3:**
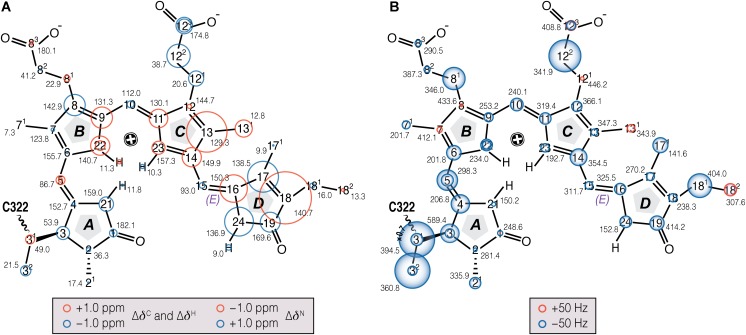
Chemical shift and FWHM line-width (*ν*_1/2_) changes of chromophore ^13^C and ^15^N resonances associated with Pr→Pfr photoconversion in oat phyA3. **(A)** The ^13^C, ^15^N (N21–N24), and ^1^H (H^N21 - N24^) chemical shifts of the chromophore in Pfr are labeled (black numbers) and their corresponding changes associated with photoconversion represented as red and blue circles (for down- and up-field shifted as Pfr, respectively; Supplementary Tables S1, S2). **(B)** The *ν*_1/2_ values of the Pfr resonances are labeled in black numbers. Pr-to-Pfr *ν*_1/2_ changes of the chromophore resonances are represented as red and blue circles (for broader and narrower resonances as Pfr, respectively; Supplementary Table S4). Atoms showing signal doublings are labeled with two circles.

The *δ*^C^ and *δ*^N^ changes (Δ*δ*) of the chromophore associated with photoconversion (**Figure 3A**, Supplementary Table S1) indicate that the ***C***- and ***D***-ring regions are predominantly affected. In particular, the C13, C16–C18 and N24 signals shift dramatically relative to Pr, together with the C13^1…^C17 DARR contact – seen only in Pfr – providing clear evidence for a ***D***-ring photoflip. Δ*δ*^C^ of the ***C***-ring propionate carbons reflect structural rearrangements of the binding pocket during photoconversion. Further details of these changes are provided by the interfacial ^1^H contacts of the chromophore atoms (see Discussion). Whereas in the ***A***–***B*** ring region most carbon atoms show only small changes upon photoconversion, larger Δ*δ*^C^ values are seen for the ***A***-ring linkage to the protein at C3, C3^1^, and C3^2^. State-related *δ*^C^ changes in the conjugated *π*-system of the chromophore are also apparent (**Figure 3A**).

Our earlier studies revealed that the Pr state is structurally heterogeneous in both oat phyA3 and Cph1, several carbons in/around the ***A***-ring region, the ***C***-ring propionate and several protein residues nearby showing at least two conformations associated with different hydrogen bonding networks (Song et al., 2013). Heterogeneity in Pfr has also been described on the basis of resonance Raman and ultra-fast absorbance spectroscopy (Kim et al., 2014; Velázquez Escobar et al., 2015; Stensitzki et al., 2017). In the present work, the N22 peak at ∼141 ppm in the 1D ^15^N spectrum (**Figure 2B**) is asymmetrical and significantly broader than the other three, deconvolution revealing components at 140.7 and 142.1 ppm with an intensity ratio ∼0.7:0.3 (**Figure 2B**), perhaps implying heterogeneity at this point. Moreover, the ^15^N line-shapes become more Gaussian and less Lorentzian at -40°C relative to those obtained from the spectra recorded at temperatures just below the freezing point of the sample solution (*e.g.*, <-17°C), indicating an increased distribution of chemical shifts at lower temperatures. On the other hand, Pfr heterogeneity was apparent in neither the DARR spectra (**Figure 1**) nor interfacial ^1^H contacts of the Pfr chromophore (see below). Indeed, the line-widths of the ^13^C and ^15^N signals of the chromophore are distinctly narrower in Pfr than in Pr (**Figure 3B**, Supplementary Table S4). Pfr line narrowing is most dramatic for the ***A***-ring linkage to the protein at C3, C3^1^ and C3^2^. The C8^1^, C12^2^, C17^1^ and C18^1^ peripheral carbons of rings ***B***–***D*** also narrow. The observed ^13^C and ^15^N line-narrowing in the Pfr chromophore does not result from an increase in spin-spin relaxation time (Siemer et al., 2012) but rather from a general *decrease* in structural heterogeneity of the surrounding protein relative to Pr.

### Interfacial ^1^H Contacts of the Chromophore in Its Pfr State

As for Pr (Song et al., 2012), ^1^H^residue^–^13^C^PCB^ contacts of oat phyA3 in Pfr were identified using MELODI–HETCOR and its variant SIDY with the help of QM/MM calculations (see Supplementary Materials and Methods). For instance, the side chain of conserved H323 (oat phyA3 numbering used throughout the article, otherwise noted) showed contacts to the pyrrolic carbons of rings ***B*** and ***C*** as well as their propionates in Pr, the corresponding ^1^H shifts nicely distinguishing cationic and neutral *τ* tautomers of the imidazole in the two Pr isoforms (Song et al., 2012). However, the Pfr spectra imply that the side chain is neutral (Supplementary Figures S2, S3 and Supplementary Tables S5–S9). We thus conclude that H323 exists in the N*𝜀*2-protonated *τ* tautomeric form in the Pfr state, well supported by both our earlier MAS NMR studies (Song et al., 2011b) and a recent investigation combining pH-dependent resonance Raman spectroscopy and QM/MM calculations (Velázquez Escobar et al., 2017a) on Cph1. Moreover, our starting Pfr QM/MM model was based on the BphP *Pa*BphP Pfr structure (PDB code 3C2W), consequently with ring ***D*** on the *α*-face (***D***-*α*_f_) relative to rings ***B*** and ***C*** (Yang et al., 2008). Such a ***D***-*α*_f_ disposition proved inconsistent with many ^1^H contacts of the chromophore resolved in the two correlation spectra, in particular for ***D***-ring methyl groups like C17^1^ and C18^2^. A *β*-facial ring ***D*** (***D***-*β*_f_) allowed most of its ^1^H contacts to be assigned straightforwardly (Supplementary Table S9). The final models for Pfr and Pr are presented as PDB files in the Supplementary Data Sheet S1. The assignments have been deposited as BMRB acquisition 27434.

### Conformational Changes in Residues Surrounding the Chromophore

**FIGURE 4:**
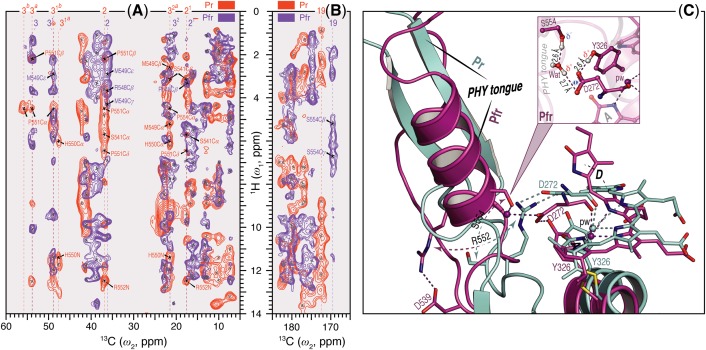
Sheet-to-helix refolding in the tongue region associated with Pr→Pfr photoconversion in oat phyA3. **(A)** The 2D SIDY data in both states (Pr, red and Pfr, purple) were collected with a ^1^H spin diffusion mixing time of 800 μs and CP contact time of 96 μs. The spectral region 5–60 ppm in the *ω*_2_-dimension and 0–14 ppm in the *ω*_1_-dimension is shown for the ***A***-ring ^1^H contacts with the tongue residues (see also Supplementary Figures S3, S5). **(B)** The 2D MELODI–HETCOR data were acquired with an LG–CP contact time of 2.3 ms. The spectral region 165–185 ppm in the *ω*_2_-dimension and 0–14 ppm in the *ω*_1_-dimension is shown for ***D***-ring C19 ^1^H contacts with the tongue residues (see also Supplementary Figures S2, S4). ^13^C resonances of the ***A***-ring carbons **(A)** and C19 **(B)** are indicated by vertical lines and their ^1^H contacts of the tongue residues are labeled in red and purple as Pr and Pfr, respectively. Non-^1^H^tongue^–^13^C ***^A^***^/^***^D^***^-ring^ correlations resolved in the spectra are marked × in gray. **(C)** QM/MM-optimized structure models of oat phyA3 in the Pr (gray) and Pfr (purple) states show a sheet-to-helix refolding in the tongue region as confirmed in the SIDY **(A)** and MELODI–HETCOR **(B)** spectra. For example, the Pr-state *β*-sheet (gray) forms a R552/D272 salt-bridge hydrogen-bonded to the chromophore, whereas in the Pfr-state *α*-helix (purple) S554 acts as a hydrogen-bond donor via a water molecule to D272 of the GAF domain which in turn interacts with the hydroxyl group of Y326 (see *inset*). R552 and S554 form part of the conserved PRXSF motif of the PHY tongue.

Unlike Pr, only local Pfr conformational heterogeneity was detected in the chromophore (N22 in ring ***B***) and none within ∼6.0 Å of its immediate protein environment, indicating that the pocket is much more rigid in Pfr. The NTE too is likely to be less mobile in Pfr, as a single ^1^H contact with C2 of ring ***A*** was seen in Pr, yet 15 SIDY contacts (mainly with the ethylidene and methyl substituents of ring ***A*** and C5) were apparent in Pfr (Supplementary Table S7). Whereas in Pr, MELODI–HETCOR (Supplementary Figure S2) and SIDY (Supplementary Figure S3) detected several correlations connecting P540, S541 and M549–R552 of the tongue to ***A***-ring carbons, of these only M549 was detected in Pfr, albeit via different carbon atoms (Supplementary Table S9). Indeed, from the available crystal structures as well as our QM/MM model for Pfr, the other tongue residues would be too distant to interact with the chromophore. Besides M549, in Pfr the ***D***-ring carbons contacted S554 from the conserved PRXSF^551-555^ motif (**Figures 4A,B**). R548, another tongue residue seen by SIDY only in Pfr, showed two contacts to ring ***A*** at C2 and C2^1^ (Supplementary Table S8). The Pfr-state QM/MM model corroborated this *a priori*, whereas in Pr R548 would be much too distant to be detected.

**FIGURE 5:**
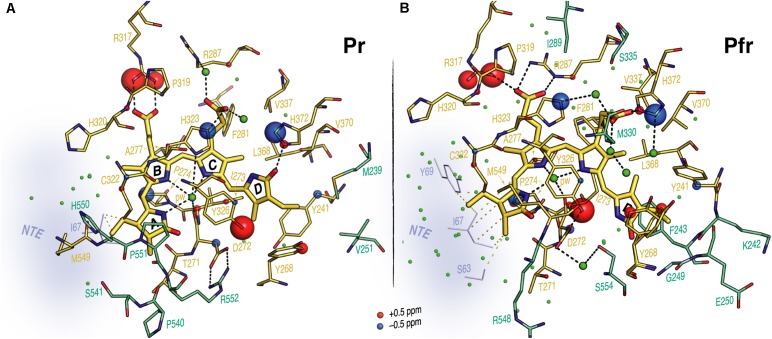
Interfacial ^1^H contacts of the PCB carbon atoms in oat phyA3 as Pr and Pfr. Structural views show the chromophore and its ^1^H residue contacts observed with MELODI–HETCOR and SIDY experiments for **(A)** Pr and **(B)** Pfr (all resolved ^1^H–^13^C contacts summarized in Supplementary Table S9). For both states, the 3D visualizations use the oat phyA3–PCB models optimized by QM/MM. Amino acids with at least one observable ^1^H contact in both states are shown as yellow sticks. The light-driven *δ*^H^ changes are represented as red and blue spheres representing down- and upfield shifts, respectively. Amino acids resolved only in Pr or Pfr are colored green-cyan. The NTE region is shown in blue-gray and its possible interfacial contacts with the chromophore are shown as yellow dashes. Potential hydrogen-bonding interactions are shown as black dashes. Water molecules are shown as green spheres.

Interfacial ^1^H contacts of the chromophore in Pr and Pfr are given in Supplementary Table S9, amino acids with at least one ^1^H contact in either Pr or Pfr are shown in **Figure 5**. Prominent changes were detected for R317 (at H*η*1 and H*η*2) and Y326 (H*η*). The ***B***-ring propionate likely forms a salt bridge with R317 in Pr, but the Δ*δ*^H^ values of ∼1.5 ppm associated with photoconversion and the contacts between the ***B***-ring propionate and R287 seen only in Pfr indicate a light-driven partner swap associated with a shift of the two inner rings, also implied by changes in ^1^H contacts to I273 and P274 (Supplementary Table S9).

Contacts between ring ***D*** and Y241, Y268 and Y326 which form a hydrophobic subpocket around it (**Figure 5**) are radically different in Pr and Pfr (Supplementary Table S9), indicating dramatic structural rearrangement of their phenolic side chains. C17^1^ and C18^1^ interact with different sides of the Y241 ring in Pr and Pfr. Indeed, our QM/MM models suggest a *t*,*g*^+^ rotamer (*χ*_1_ = 191°, *χ*_2_ = 61°) for this tyrosine in Pr and a *g*^-^,*t* rotamer (*χ*_1_ = 304°, *χ*_2_ = 333°) in Pfr (see Supplementary Materials and Methods for rotamer definitions), associated with a reduction of ∼1.5 Å in the (*χ*_1_,*χ*_2_)-dependent C*𝜀*2–N*α* internuclear distance. Similarly, large changes were observed for Y268: in Pr its phenolic ring is close to C17^1^, C18^1^ and C18^2^ of the ring ***D***, whereas in Pfr only a single ^1^H contact to C18 was apparent (Supplementary Table S9). The hydroxyl of Y326 on the opposite bilin *α*-face correlated with C15 and C16 (**Figure 4C**, Supplementary Figure S4, and Supplementary Table S9). Although the global *δ*^H^ changes of the Y326 ring and a +1.7 ppm Δ*δ*^H^ for H*η* might be interpreted as hydrogen bonding to N24 in Pfr, the associated contact to C19 is clearly absent (Supplementary Figure S4 and Supplementary Table S9). Indeed, with a likely ***D***-*β*_f_ such an interaction would be impossible (see Discussion). Further prominent *δ*^H^ changes associated with photoconversion were detected for the imidazole rings of H372 and the neighboring H323 (Δ*δ*^H^ for H*𝜀*2 of -1.4 and -1.2 ppm for H372 and H323, respectively; Supplementary Table S9).

## Discussion

^13^C–^13^C DARR and ^1^H–^13^C/^15^N HETCORs provided insight into the structure/functional relationships in both the inactive Pr and signaling Pfr states of the chromophore and ^1^H neighbors up to ∼6 Å distant in oat phyA3. With the help of QM/MM-based models and complete ^13^C and ^15^N assignments of all 37 PCB carbon and nitrogen atoms (**Figure 3A** and Supplementary Table S1), we tentatively identified many specific chromophore–protein contacts (^1^H^residue^–^13^C^PCB^) in both Pr and Pfr (Supplementary Figures S2–S5 and Supplementary Table S9; Song et al., 2012). Although these data alone are still far from providing complete 3D structural solutions, our work provides novel insight into the characteristics of A-type plant phytochromes in both parent states, as well as the underlying photoconversion mechanisms.

### Chromophore

The present work relies on generating holophytochrome in which the chromophore but not the apoprotein is isotopically labeled, exploiting the *in vitro* assembly reaction typical of the phytochrome family. We caution that, at least in the case of Cph1, small but perhaps functionally significant differences exist between material produced *in vitro* and *in vivo*, perhaps as a result of cotranslational assembly and thus different cooperative folding. In the case of phyA3 from oat there seems to be no functional difference between the holoproteins extracted from plants and apoprotein assembled *in vitro* (Schmidt et al., 1998).

All the chromophore carbon and nitrogen atoms in both states are now assigned definitively (**Figures 1**–**3A** and Supplementary Table S1), the DARR correlations showing the chromophore to be periplanar *ZZZssa* and *ZZEssa* in Pr and Pfr, respectively. For example, H^N24^ correlated with the ring ***C*** at C13, C13^1^, and C14 in Pr but not Pfr (Supplementary Table S1), whereas a DARR correlation connecting C13^1^ of ring ***C*** and C17 of ring ***D*** is only seen in Pfr (Supplementary Figure S1). Furthermore, the interfacial correlation data show ring ***D*** to be *α*- and *β*-facial in Pr and Pfr, respectively, geometries corresponding to those for Cph1, whereas in bacteriophytochromes both states are *α*-facial. This difference was implied by CD spectroscopy (Rockwell et al., 2009). In that study it was suggested that the direction of isomerization in plant phytochromes and Cph1 is anticlockwise, the slump arising from steric hindrance of the ring ***D*** by the ***C***-ring methyl side chain leading to rotation around the C14–C15 single bond, whereas in bacteriophytochromes the likely direction of isomerization would be clockwise leaving the ***D***-ring at the α face. A ***D***-*β*_f_ Pfr would require radical changes in the cavity accommodating ring ***D*** relative to the situation in BphP Pfr. Indeed, the present study reveals exactly such changes (see below).

Although most intramolecular ^1^H contacts (^1^H^N21-N24^) of the ***A***-ring carbons are unaffected by photoconversion (Supplementary Table S2), H^N21^ of ring ***A*** exhibited a weak but clearly resolved contact with C9 of ring ***B*** in the ^1^H–^13^C MELODI–HETCOR spectrum solely in Pfr (**Figure 2A**, purple; Song et al., 2012), implying a modified distortion at the ***A***–***B*** methine bridge in response to local protein structural changes during photoconversion. Such changes were probably the origin of the confusion that the ring ***A*** photoisomerizes (see Song et al., 2014).

The Δ*δ*^C^ pattern shown in **Figure 3A** indicates electronic rearrangement in the chromophore *π*-system associated with photoconversion. In Pfr, whereas the Δ*δ*^C^ values alternate for the conjugated chains of rings ***A***–***B*** and ring ***D***, all four ^13^C signals of the ring ***C*** are uniformly shifted upfield relative to Pr, indicating a local electron density increase in Pfr. This might be a general event in the phytochrome family, as nearly identical effects were also seen in Cph1 (Supplementary Figure S6). The interruption of the alternating pattern at ring ***C*** is also seen in the phytochrome-related CBCRs fragments such as AnPixJg2 (Song et al., 2015) and NpR6102g4 (Rockwell et al., 2015) during their red/green photocycles (Supplementary Figure S6). State-related *δ*^C^ changes in the *π*-conjugated C4–C19 systems in oat phyA3 and Cph1 are generally similar (Supplementary Figure S7), particularly so for carbons associated with the ***A***–***B*** and ***B***–***C*** methine bridges as well as C17 of the ring ***D***, implying similar electronic changes. The smaller Δ*δ*^C^ values for C14 and C16 in oat phyA3 relative to Cph1 might arise from the larger tilt angle between rings ***C*** and ***D*** (the angle between the normal vectors) in plant phytochromes. Our Pr model for oat phyA3 predicts 57°, whereas 46° was predicted for *Arabidopsis* phyA (Mroginski et al., 2011). The Cph1 crystal structure indicates a shallower angle of 27° (Essen et al., 2008), whereas that for *Arabidopsis* phyB implies ∼60° (Burgie et al., 2014a). A ***D***-ring twist greater than ∼40° would significantly reduce conjugation with the rest of the *π*-electron system, leading to *δ*^C^ changes localized to the ***C***–***D*** methine bridge and ring ***D***. The differences observed at C13 and C18 in the present study also support such an effect (Supplementary Figures S6, S7).

Although changes in the extent of conjugated *π*-electron systems lead to shifts in the absorbance *λ*_max_, it is by no means clear how or even if the R/FR photochromicity characteristic of canonical phytochromes derives from this. The conjugated system would have to be longer in Pfr than Pr to induce the bathochromic absorbance shift: yet, on the contrary, following denaturation Pfr is *hypso*chromically shifted relative to Pr (Zhao and Scheer, 1995; Ishizuka et al., 2007; Hirose et al., 2008; Song et al., 2014). Interaction with the protein pocket is thus fundamentally significant, but the actual effect is unknown. Progress here will probably require an adequate understanding of the border orbitals of both Pr and Pfr.

Our ^1^H and ^15^N Pfr data of the pyrrole NH groups (**Figure 2**) give clear evidence that a protonated cationic bilin ring system is present in both Pr and Pfr, paralleling conclusions from earlier work on prokaryotic phytochromes (Hahn et al., 2008; Rockwell and Lagarias, 2010). State-induced *δ*^H^ changes seen at the two NH protons associated with rings ***B*** and ***C*** (**Figure 3A**) reflect changes in the pocket, in particular for the highly conserved D272 and H323 as well as the pyrrole water, all of which are within hydrogen-bonding distance (**Figure 5**). Indeed, the *δ*^N^ values calculated quantum-mechanically for the chromophore nitrogens in the Pfr state agree well with those measured experimentally (Supplementary Table S3). Moreover, the ^15^N Pfr spectra of oat phyA3 and Cph1 are remarkably similar (Supplementary Figure S8), all four maxima deviating by less than 1.5 ppm and the N22 signal showing a similar splitting. The Pr→Pfr Δ*δ*^N^ patterns are similar too (Supplementary Figure S6). This resemblance suggests that the hydrogen bonds connecting the four pyrrole nitrogens are very similar. Interestingly, N22 of ring ***B*** in oat phyA3 and Cph1 in both states resonates at least 13-ppm upfield of N23 of ring ***C*** (Supplementary Table S1). We have proposed that the distinct *δ*^N^ difference between these inner ring nitrogens arises from the asymmetry of the strong hydrogen-bonding interactions with the nearby carbonyl backbone of D272 (D207 in Cph1) and the pyrrole water (Rohmer et al., 2010). Recent DNP-enhanced MAS NMR studies on Cph1 in Pr have suggested that the positive charge carried by the tetrapyrrole system is mainly localized at ring ***B*** and thus would account for a downfield shift of N22 relative to that of N23 (Stöppler et al., 2016).

### Bilin–Protein Linkage

Subtle but significant state-related changes were detected at the ***A***-ring ethylidene linkage to the protein (Supplementary Table S1). Similar Δ*δ*^C^ values at these carbons are also seen in Cph1 (Supplementary Figure S6) and a non-canonical Group II phytochrome *SyB*.Cph2(GAF) fragment (Ulijasz et al., 2010). Moreover, in the case of the red/green CBCR NpR6102g4, much more dramatic shifts of the ethylidene carbons of ring ***A*** and on the three methine bridges were found (Rockwell et al., 2015; Supplementary Figure S6), implying a stronger mechanical distortion of the bilin chromophore upon photoproduct formation. The pattern of ^13^C line-width changes at the linkage is also similar to that in Cph1 (Supplementary Figure S9). Changes in interactions with the protein in this region probably result from restructuring of the binding pocket deriving from the ***D***-ring movement rather than from autonomous movements of the chromophore. Whereas significant changes in *δ*^C^ and line-widths at the ***A***-ring linkage to the protein imply that covalent attachment is functionally important, biochemical evidence contradicts this: mutation or chemical blocking of the Cys attachment site prevents covalent attachment, yet the chromophore is still bound and photochromicity seen, the observed bathochromic shifts in *λ*_max_ being consistent with the more extensive conjugated *π*-system of the unattached chromophore (Jorissen et al., 2002; Lamparter et al., 2002). Signaling in plant phyB requires the chromophore, but not covalent attachment (Oka et al., 2011).

### N-Terminal Extension

**FIGURE 6:**
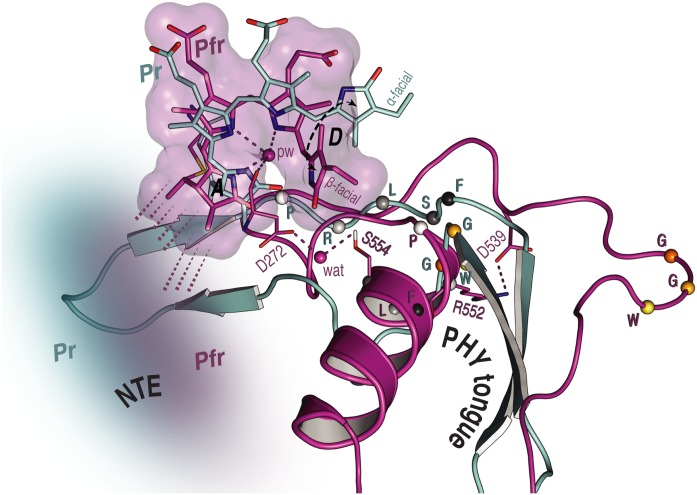
Overview of the NTE activity and tongue refolding caused by Pfr formation in oat phyA3. The NTE region (shown as a cloud) is more compact with the Pfr chromophore (purple) relative to that in Pr (cyan), as indicated by dashed lines. For the tongue region undergoing an *α*-helix formation and a *β*-sheet melting, the conformational rearrangement of the conserved PRXSF motif and WGG motif is highlighted (amino acids shown as spheres).

Unfortunately, beyond secondary structure predictions, there exist no structural data for the NTE of any plant phytochrome. Indeed, even in the case of prokaryotic phytochromes, the structure of the (much smaller) NTE seems to be influenced critically by subtle interactions with the tip of the tongue-like hairpin extending from the PHY domain. For example, the PAS–GAF and PHY domains in wild-type (2VEA) and Y263F mutant (3ZQ5) crystal structures of the Cph1 Pr sensory module (Essen et al., 2008; Mailliet et al., 2011) are almost identical except at that point. The fact that deletion of the NTE in plant phytochromes leads to a hypsochromic shift of the Pfr absorption maximum and accelerated dark reversion implies, however, that its interactions with the sensory module are functionally important. Indeed, for example Jordan et al. (1995, 1997) found that, whereas alanine substitutions in the serine-rich 2–18 region lead to enhanced physiological sensitivity to light, modifications to the downstream region lead to hypsochromic shifts particularly of Pfr and accelerated Pfr→Pr dark reversion. The current study provides insight into the behavior of the NTE at the molecular level. Whereas our SIDY experiments revealed only one ^1^H NTE contact in Pr, no less than 15 interactions were detected in Pfr. Although we are not yet able to assign these contacts with certainty, we consider that they involve I67 in Pr and predominantly Y69 in Pfr (Supplementary Figures S3, S5 and Supplementary Table S8). Similarly, in MELODI–HETCOR we observed 16 contacts likely to involve the NTE in Pfr but none in Pr (Supplementary Figures S2, S4 and Supplementary Table S6). The much more extensive interaction between the NTE and the chromophore in Pfr than in Pr derives from hydrogen bonds from the hydrophobic side of ring ***A*** (**Figures 5**, **6**). The Pfr peaks are also narrower than those in Pr (**Figure 3B**), consistent with reduced mobility. The state-dependent changes in dynamics of the NTE based on HDX-MS data for *Arabidopsis* phyB (von Horsten et al., 2016) showed two points in the NTE with more rapid H/D exchange in Pfr than in Pr, implying increased mobility or accessibility in Pfr. It is important to note, however, that the phyA and phyB sub-families have very different physiological roles in plants and that the NTE in phyB is always much larger and shows no sequence similarity to that of phyA.

### Tongue

We observed a number of Pr correlations connecting the chromophore to five residues of the tongue-like extension of the PHY domain in the MELODI–HETCOR spectrum (Supplementary Table S6), implying that the tongue is bound strongly to the rest of the protein. R552 from the conserved PRXSF^551-555^ tongue motif is also seen to contact ring ***A*** in the SIDY spectrum, commensurate with a salt bridge to D272 (**Figure 4A**). In the corresponding Pfr spectra, however, only R548, M549 and S554 were detected (**Figure 4A** and Supplementary Table S9), implying that the S554 side chain points away from the chromophore in Pr but is rotated inward to face ring ***D*** in Pfr (**Figure 4C**). The S554 hydroxyl group probably hydrogen bonds to D272 and via a strategically-placed water to rings ***A***–***C*** in Pfr (**Figure 4C**, *inset*), replacing the D272–R552 salt bridge which connects the chromophore to the tongue in Pr. These findings accord with the likely changes associated with photoconversion derived from Pr and Pfr structures of Cph1 and bacteriophytochromes. A particular R residue in the tongue specific to the ‘bathy’-subgroup of bacteriophytochromes has been suggested to be an important factor in promoting Pfr formation in darkness (Velázquez Escobar et al., 2017b). Interestingly, this residue is R/K in plant phytochromes (R548 in oat phyA3), none of which show the ‘bathy’ phenotype. This residue might nevertheless help stabilize Pfr. However, although we indeed see contacts between the ring ***A*** and R548, they are to C*β*, not to the guanidinium moiety (Supplementary Table S9).

These data support the notion that the tongue forms a helix in Pfr in contrast to antiparallel sheets in Pr in the case of oat phyA3 (**Figure 4C**), as has been proposed for prokaryotic phytochromes (Anders et al., 2013; Stojković et al., 2014; Takala et al., 2014). Recent HDX-MS experiments on *Arabidopsis* phyB (von Horsten et al., 2016) and the non-canonical BphP *Is*PadC (Gourinchas et al., 2017) also imply reduced dynamics in the WGG motif region (533–540 in oat phyA3) in Pr (**Figure 6**). Although tongue refolding and associated shifts of the PHY domains in the dimer offer an attractive model for downstream intramolecular signaling at least in prokaryotic phytochromes, we note that the *Deinococcus* BphP (*Dr*BphP) Pfr crystal structure was an inappropriate model for the small-angle X-ray scattering data (Takala et al., 2014). The recent structure/functional analysis of *Is*PadC also questions the causal role of tongue refolding in PHY domain shifts and signaling (Gourinchas et al., 2017).

### Arginine Swap

**Figure 5** shows the prominent *δ*^H^ changes associated with the conserved arginine pair R317 and R287, the carboxylate oxygens of the ***B***-ring propionate associating with R317 in Pr, but with R287 in Pfr. This could arise from a shift of rings ***B*** and ***C*** relative to the protein as implied by the ^1^H contacts involving I273 and P274 of the conserved DIP motif (Supplementary Table S9) that provide hydrophobic packing contacts for the ***B***–***C*** plane from the *β*-face. These contacts would be particularly sensitive to movements of chromophore and/or its pocket. Such a propionate partner swap was proposed for Cph1 in analogy to the heme-based oxygen sensor FixL, the ***B***-ring propionate swapping from R254 in Pr to R222 in Pfr, perhaps with the function of transmitting the light-signal to the protein surface (Essen et al., 2008; Song et al., 2011b). Although, surprisingly, the *Dr*BphP Pr and presumptive Pfr structures described by Takala et al. (2014) showed no such rearrangements, the partner swap is clear in the equivalent structures of Burgie et al. (2014b, 2016) for the same molecule. The recent crystal structure of an *Arabidopsis* phyB sensor fragment (Burgie et al., 2014a) is ambiguous in this respect, however, showing one subunit with the ***B***-ring propionate clearly salt-bridged to R352 while in the other the arginine is twisted away. The partner swap may be important in plant phytochrome signal transduction via PIF (phytochrome-interacting factor) transcription factors (see below).

### Histidine Interactions

Prominent *δ*^H^ changes upon photoconversion were also seen for H372 and the neighboring H323. In the case of H372 this is due to the hydrogen-bonding partner-swap of its imidazole ring (N*𝜀*2) with the chromophore during the photoflip. Specifically, H372 hydrogen-bonds to the ***D***-ring carbonyl group in Pr, but to the ***C***-ring propionate carboxylate group in Pfr (**Figure 5**). C12^3^ interacts in Pfr with H372 at H*𝜀*2, and accordingly, the model predicts a C12^3^-O^…^H372-N*𝜀*2 distance of 3.2 Å, consistent with a hydrogen bond. The formation of such a bond in Pfr is facilitated by the positional shift of the inner rings and the ring rotameric flip of H372 (**Figure 5**).

The *δ*^H^ changes associated with the imidazole ring of H323, particularly the prominent Δ*δ*^H^ of -1.2 ppm seen at H*𝜀*2 (Supplementary Table S9) might arise from either a hydrogen-bond partner-swap or a rebalance between hydrogen bonding and geometric strain caused by a change in the imidazole rotameric conformation (**Figure 5**). As in other phytochromes, this histidine is strategically placed above the ***A***–***C*** ring nitrogens, an intricate web of hydrogen bonds connecting them to the histidine via the pyrrole water and thence to the ***C***-ring propionate carboxylate group. Conversely, in Pfr, H323 is bridged via another water molecule to the ***C***-ring propionate and further to H372 (**Figure 5B**). In this way, the bilin chromophore protonates itself rather than the wall of the binding pocket providing an appropriately acidic side chain. Moreover, in H260Q of Cph1 pH values above 7.5 lead to chromophore deprotonation in both states (Hahn et al., 2006), consistent with a role for H260 in protonation dynamics.

### Tyrosine Triad

According to the available crystal structures, three conserved tyrosine residues attend ring ***D***, the two on the *β*-face of the chromophore generally adopting very different side-chain positions in Pr and Pfr. These residues are Y241, Y268 and Y326 in oat phyA3, our data providing novel insights into their roles in this and perhaps other phytochromes.

Y241 clearly shifts radically upon photoconversion (**Figure 5** and Supplementary Table S9), the atomic contacts and QM/MM modeling implying a similar flip to that seen in bacteriophytochromes (Yang et al., 2008; Takala et al., 2014) with the ring roughly parallel and perpendicular to the chromophore in Pr and Pfr, respectively. Y241H-homologous substituents are intensely fluorescent in Cph1 (Fischer and Lagarias, 2004) and plant phytochromes but not in bacteriophytochromes. Remarkably, in plant phytochromes these mutants show a *cop* (constitutively photomorphogenic) phenotype, implying that they mimic Pfr in darkness (Su and Lagarias, 2007).

Y268-homologous residues in bacteriophytochromes generally show a side-chain flip too but in the opposite direction, such that the ring is perpendicular and parallel to the chromophore in Pr and Pfr, respectively. This is not feasible in plant phytochromes and Cph1, however, because the ***D***-ring slumps in Pfr to occupy the position that the Y268 ring would have adopted (**Figure 5**). Applying QM/MM calculations to the two different starting points consistent with the NMR data reported here for Pfr, QM/MM calculations yielded similar optimized structures in which the Y268 ring is roughly perpendicular to the chromophore plain, as in Pr (**Figure 5B**). Given that the known Pr structures of bacteriophytochromes are quite similar to those of Cph1 and *Arabidopsis* phyB in this region, in Pfr the radically different positioning of Y268 in phyA relative to that in bacteriophytochromes is remarkable – especially as the photochromicity seems to be similar too. However, the CD data implying that the chirality of the chromophore inverts upon photoconversion in Cph1 and plant phytochromes but not bacteriophytochromes is unambiguous (Rockwell et al., 2009). Moreover, we found no way to reconcile the NMR data reported here with a ***D***-α_f_ in Pfr. In contrast to bacteriophytochromes, the ***D***-*β*_f_ in our Pfr model shows no hydrogen bonding but is in van der Waals contact with Y268, perhaps providing an alternative stabilizing force. It was also recently found that Y268V substituents in oat phyA3 and their homologs in phyB show a *cop* phenotype similar to that of Y241H-homologous substituents (Jeong et al., 2016).

As in bacteriophytochromes, we found only subtle effects of photoconversion on the geometry of the third member of the triad, the α-facial Y326. In the prototypical ‘bathy’-type *Pa*BphP 3C2W Pfr structure (Yang et al., 2008), the homologous hydroxyl was suggested to hydrogen-bond to ring ***D***, and indeed our MAS NMR data for Cph1 Pfr (Song et al., 2011b) implied that a similar bonding might take place. However, this seems not to be the case at least in oat phyA3 Pfr. Although our NMR data for Pfr reveal contacts between the Y326 hydroxyl group (H*η*) and chromophore C15 and C16, indicating proximity to rings ***C*** and ***D*** (Supplementary Table S9), not only is the expected Y326-H*η*^…^C19 contact missing (**Figures 4C**, **5B**), QM/MM robustly exclude hydrogen bonding. Nevertheless, this tyrosine is perfectly conserved, so what important role does it play if not in hydrogen bonding to N24 in Pfr? In accordance with our data and QM/MM calculations, we propose that it is involved in hydrogen bonding to S554 of the tongue (**Figure 4C**, *inset*), thereby stabilizing its Pfr-specific helix, the formation of the hydrogen bond being supported by a +1.7 ppm Δ*δ*^H^ of the Y326-O*η* group (Supplementary Table S9).

### Dynamics and Heterogeneity

Our data imply reduced structural heterogeneity in Pfr relative to Pr at the ***A***-ring, the ***C***-ring propionate and a number of neighboring residues (Supplementary Tables S1, S9; Song et al., 2012). The overall pattern of ^13^C and ^15^N line narrowing is similar to that of Cph1 (Supplementary Figure S9) although the region involved is smaller in phyA3 (**Figure 3A** and the overlaid Pr and Pfr ^1^H–^13^C correlation spectra in **Figures 4A,B**). The observed ^13^C and ^15^N line-narrowing in Pfr results from greater structural homogeneity and reduced mobility of the surrounding protein relative to Pr in which the chromophore is loosely embedded in a soft pocket. Resonance Raman spectra of *Xanthomonas* BphP also imply increased dynamics in the Pr chromophore (Otero et al., 2016), whereas other prokaryotic phytochromes showed greater Pfr chromophore heterogeneity (Salewski et al., 2013; Kim et al., 2014; Velázquez Escobar et al., 2015; Stensitzki et al., 2017). Even if these effects also exist in plant phytochromes, we suspect that their dynamics would be too fast and/or the movements too subtle to be detected by MAS NMR.

Such concerted, widespread changes of order at the mesoscopic scale offer an alternative paradigm in protein function, very different from that of a chemical reaction. We proposed that these effects reflect a mesoscopic phase transition from soft and unordered to hard and ordered, presumably caused by a large-scale charge redistribution (Song et al., 2011a, 2013). Local changes in molecular dynamics might be important in photoconversion and signaling by providing access to alternative topologies, as suggested by the capture-and-collapse model (Stewart et al., 2016; see also below). On the other hand, the ‘*soft*’ state would allow for fast energy dissipation which is needed to keep a product in a local minimum, whereas the ‘*hard*’ state might provide for mechanochemistry and organized tension.

### Photoconversion and Physiological Signaling

Nearly all protein structural data are static, and even NMR can only hint at the dynamics underlying functional mechanisms. Nevertheless, the extensive information provided by phytochrome crystal structures together with NMR and other spectroscopic techniques provides insight into the processes underlying photoconversion between Pr and Pfr. Here we summarize changes and possible mechanisms involved in the photoactivation of plant phytochromes and the associated cell-physiological responses, abstracting ideas accumulated in earlier proposals and from the work presented here.

The resting Pr state shows an intense (*𝜀* ≈ 100 mM^-1^ cm^-1^), sharp absorption peak at ∼660 nm corresponding to a cationic bilin chromophore with periplanar *ZZZssa* geometry. The proton probably originates from the ***C***-ring propionic carboxyl itself and is transferred via H323 and the pyrrole water above the ***A***–***B***–***C***-ring nitrogen triad. It seems that Pr comprises at least two sub-states, Pr-**I** and Pr-**II** (Song et al., 2011b, 2013). Upon photon absorption, ring ***D*** of the Pr-**II** sub-state alone is able to achieve the anticlockwise *Z→E* photoflip to Lumi-R arising from C15=C16 isomerization (Müller et al., 2008; Song et al., 2011b; Yang et al., 2014). Simultaneously, steric interaction between the ***C***- and ***D***-ring methyl side chains pushes the ring ***D*** downward to a *β*-facial disposition by rotation around C14–C15 (Rockwell et al., 2009; Song et al., 2011b; Yang et al., 2014). Although the 660-nm photon energy is more than sufficient to power the photoflip, how it occurs is unclear. It is probably significant, however, that the bond order is reduced in S_1_, lowering the activation energy for double-bond isomerization. Photochemical impotence of the Pr-**I** sub-state would help to explain the modest overall photoconversion quantum efficiency of ∼0.15 (Yang et al., 2014).

The photoflip necessarily breaks the Pr-stabilizing hydrogen bond between the ***D***-ring carbonyl oxygen and H372. Anticlockwise rotation of the ring would force it to slump because of steric interactions between the ***C***- and ***D***-ring methyl groups. The Y241 ring flips from parallel to perpendicular relative to the plane of chromophore rings ***B*** and ***C***, whereas — in contrast to bacteriophytochromes — Y268 seems only to shift slightly on photoconversion. Also on account of its *β*-facial disposition, the ***D***-ring nitrogen in Pfr is hydrogen-bonded neither to Y326 nor to D272 (**Figure 5B**). Instead, it seems to be stabilized by van der Waals interactions with Y268.

The initial *ZZEssa* ground-state photoproduct, Lumi-R, is followed by spectroscopic Meta-R intermediates, the last of which prior to Pfr formation shows weak absorbance probably as a result of chromophore deprotonation accompanied by proton loss and re-uptake to/from the medium (van Thor et al., 2001; Borucki et al., 2005), the cause of which is not known. Why Pr→Pfr photoconversion is associated with a *batho*chromic shift is also unknown, but it certainly derives from changed interactions with the protein pocket rather than isomerization itself: the *ZZEssa* geometry (as in Pfr) is *hypso*chromically shifted relative to *ZZZssa* (as in Pr) under denaturing conditions (Zhao and Scheer, 1995; Ishizuka et al., 2007). We acknowledge the suggestion of Prof. Clark Lagarias (UC Davis) during the review process that photoconversion to Pfr allows entry of additional water molecules into the binding pocket, as suggested by the homology models for Pr and Pfr (**Figure 5**), constraining the 15*Ea* photoproduct and perhaps providing an explanation for the enigmatic red shift of the Pfr state.

Unfortunately, how the various spectral intermediates are associated with the dynamics of photoconversion is quite unclear. Following the photoflip, the chromophore slides in the direction of ring ***D*** relative to the protein pocket (Yang et al., 2009). Although causality is uncertain, the ***B***-ring propionate swaps its salt-bridge partner from R317 to R287 (**Figure 5**) in association with the movement, thereby perhaps inducing a small shift at the PAS–GAF domain interface and thereby Pfr-dependent binding to the PIF transcription factors (Essen et al., 2008; Kikis et al., 2009; see also below). On the other side of the chromophore, the D272–R552 salt-bridge connecting the chromophore to the tongue breaks, the tongue undergoing a remarkable sheet-to-helix refolding. Thereby, W533 sealing the chromophore pocket in Pr is replaced by F558 in Pfr, analogously to the tryptophan swap model (Anders et al., 2013; Takala et al., 2014). The Pfr-specific structure of the tongue is stabilized by a S554–D272–Y326 hydrogen-bonding network (**Figure 4C**, *inset*): accordingly, in *Arabidopsis* phyB, the S554-homologous mutant S584A shows extremely rapid Pfr→Pr dark reversion (Burgie et al., 2014a). Indeed, the tongue region seems generally to be important for Meta-R_c_ reprotonation and Pfr formation/stability.

Fundamental questions arise regarding the actual mechanism of photoconversion. The energy of a red photon is about 100-fold larger than necessary to break a hydrogen bond, but why should the ring ***D*** rotate at all when its amphiphilic character in the *Za* configuration looks rather favorable in the subpocket? Indeed, Pr-**I** is thought not to isomerize (Yang et al., 2014). Superficially, the problem is exacerbated by the fact that a second photon reverses the photoactivation process. Photoreversion, however, takes a quite different route provided by the specific Pfr structure, as mentioned above, transient deprotonation in Meta-R perhaps acting as a valve to prevent a simple back-reaction to Pr. Three points are notable regarding the forward reaction: firstly, subtle structural changes make Pfr rather than Pr the resting state in ‘bathy’-type bacteriophytochromes; secondly, several substituents of the Y residues near the ***D***-ring phenocopy Pfr in darkness (Su and Lagarias, 2007; Jeong et al., 2016); thirdly, the energy of a far-red photon is lower than usually required for photochemistry. Thus, we propose that numerous, subtle structural changes rather than a conventional local chemical reaction might lie at the heart of Pfr formation. The mesoscopic phase transition at least in the chromophore pocket as revealed by MAS NMR in both Cph1 and phyA (see above and also Song et al., 2011a, 2013) would provide the physicochemical basis for this. We suggest that the mobile Pr state is able to adopt numerous energetically similar conformations (sub-states) and that one or other of these can be captured to convert to Pfr with the help of energy from the chromophore (or subtle structural variations), following the capture-and-collapse concept (Stewart et al., 2016). The ‘cocked’ Pfr state would then be under tension and relatively immobile [as apparent from the ^13^C and ^15^N line-narrowing in the Pfr chromophore (**Figure 3B**) and implied by the highly intense 814-cm^-1^ hydrogen-out-of-plane mode observed in Raman spectroscopy; Matysik et al., 1995] but could be released to form Pr by the action of a sensitive, hairspring-like trigger requiring either a second photon, a particular biochemical environment, or just thermal fluctuations.

Atomic contacts between the NTE and the chromophore are much more numerous in Pfr than in Pr, implying novel binding to the GAF domain associated with stabilization of the Pfr state (**Figures 5**, **6**). Phosphorylation of the N-terminal S/T residues in phyA is associated with reduced sensitivity to light (Jordan et al., 1995, 1997), but how this occurs is unknown. Unfortunately, the structure of the NTE is not known for any plant phytochrome.

However it occurs, sheet-to-helix refolding of the tongue would present a radically different surface for Pfr-dependent partner interactions. It would also shorten the tongue, pulling the PHY and GAF domains together with the help of a hinge in the long connecting helix (Takala et al., 2014). Native phytochromes stably dimerize as a result of interactions between the DHp-like domains, thus such a shift would pull the neighboring PHY domains apart and probably also affect the associated PAS-domain repeats characteristic of plant phytochromes. Intriguingly, in both phyA and phyB mutations that specifically block signaling are concentrated in a small region (the Quail box) between the two PAS domains. In plants, Pr is exclusively cytosolic whereas the light-signal requires Pfr to be present in the nucleus (although see Hughes, 2013). Translocation of phyA Pfr from the cytoplasm to the nucleus is particularly efficient, requiring the FHY1 adapter protein (Hiltbrunner et al., 2005; Rösler et al., 2007). The phyA-FHY1 interaction is thought to involve the GAF–PHY region of the sensory module (Genoud et al., 2008) and thus might be regulated by tongue refolding.

Whereas canonical prokaryotic phytochromes signal via their C-terminal HK transmitter module, plant phytochromes are not HKs even though their C-termini resemble transmitters. At least in phyB, the transmitter-like module, the plant-phytochrome-specific PAS-repeat and even the PHY domain can all be deleted while retaining the light signal as long as the PAS–GAF lobe is dimerized and in the nucleus (Matsushita et al., 2003; Palágyi et al., 2010; Ádám et al., 2011; Qiu et al., 2017). The light-signal itself arises from Pfr-dependent binding of the PIF3 transcription factor to the PAS–GAF lobe, whereby the PIF is inactivated and skotomorphogenesis suppressed. Thereafter, other parts of the molecule probably assist PIF phosphorylation by MUT9-like kinases, polyubiquitination and finally destruction of both PIF and phytochrome in the proteasome (Ni et al., 1998, 1999, 2014, 2017; Al-Sady et al., 2006; Oka et al., 2008; Qiu et al., 2017). Interestingly, interaction between phyB and all PIF family members in *Arabidopsis* requires an arginine at the residue homologous to R317 in oat phyA3 (Oka et al., 2008; Kikis et al., 2009). Even small changes affecting the PAS–GAF interface arising from the ***B***-ring propionate–R317/R287 partner swap might regulate PIF binding affinity and thus the photomorphogenic signal. We caution, however, that signaling from phyA might be rather different, as phyA and phyB bind different regions of PIF3 (Al-Sady et al., 2006), thus the corresponding binding regions of the photoreceptors are quite likely to be different too. It seems nevertheless that Pfr-dependent destruction of PIF transcription factors is the major route for phytochrome light signaling in higher plants (Leivar et al., 2009).

## Conclusion

In this work we analyzed structure/functional relationships in oat phytochrome A3 by probing the PCB chromophore of the complete sensory module using MAS NMR in tandem with QM/MM methods, here concentrating on Pfr and thereby providing the first structural data of any kind for the signaling state of a plant phytochrome. Comparison with our earlier data for Pr provides insight into the mechanism of photoconversion. Many structure/functional aspects parallel those described for prokaryotic phytochromes, especially Cph1, such as the ***D***-ring photoflip, movements at the covalent linkage of ring ***A*** to the protein, the ***B***-ring propionate partner swap (**Figure 5**), and the refolding of the tongue region (**Figure 6**). Like Cph1 but in contrast to bacteriophytochromes, ring ***D*** is *β*-facial in Pfr. The far more numerous atomic contacts between the ring ***A*** and the NTE region in Pfr imply that the latter interacts more extensively with the protein in Pfr than Pr (**Figures 4**–**6**). The different mobilities of the Pr and Pfr states might represent processes fundamental to photoconversion and intramolecular signal transduction in phytochromes. Thus, although complete 3D protein structures cannot yet be derived from MAS NMR, the interfacial ^1^H^residue^–^13^C^PCB^ contacts revealed (Supplementary Table S9) provide insight into structure/functional relationships in plant phytochrome systems. Further studies are needed to clarify the relationship between the ***D***-ring photoflip, structural changes in the protein and cellular translocation and signaling.

## Materials and Methods

### Sample Preparation

The holophytochrome sample [the complete sensory module (residues 1–595 plus a C-terminal His_6_ tag) of *Avena sativa* (oat) phytochrome A3 expressed in yeast and assembled with *u*-^13^C,^15^N-PCB chromophore] used in our earlier MAS NMR study (Song et al., 2012) was irradiated to generate the Pfr/Pr 4/1 photoequilibrium mixture which was then frozen in liquid N_2_. UV-vis absorbance spectra are shown in Supplementary Figure S10, see also Mozley et al. (1997).

### Construction of a Structural Model for Oat phyA3 Pfr

The 3D structural model of the oat (*Avena sativa*) phyA3 PCB chromophore in the Pfr state was constructed in four steps: A preliminary model based on the oat phyA3 sequence and published crystal structures was produced using MODELLER and Chimera programs, then improved by crude QM/MM geometry optimization of the chromophore-binding pocket using a small QM region. That model was used for preliminary assignments and refined on the basis of NMR-determined chromophore interfacial ^1^H contacts in an iterative fashion. Finally, the refined model was subjected to QM/MM geometry optimization of the chromophore-binding pocket using a 198-atom QM region (including the chromophore, D272, I273, the side chains of C322, Y241, F243, Y268, H323, Y326 and S554 as well as three important water molecules, see Supplementary Figure S11) together with the NMR restraints. These steps as well as the procedure used to calculate the chemical shifts are described in detail in the Supplementary Materials and Methods.

### MAS NMR Data Collection

All NMR data were acquired on a Bruker AV-750 WB spectrometer (Rheinstetten, Germany) operating at Larmor frequencies of 750.13 MHz for ^1^H, 188.62 MHz for ^13^C, and 76.01 MHz for ^15^N using a 4 mm triple resonance MAS DVT probe. In this study, 15 mg (67 μL) of holophytochrome (4:1 Pfr/Pr ratio) was loaded into a 4 mm zirconia MAS rotor and frozen in liquid N_2_. In the magnet, the sample was kept at -40°C. The MAS rate of 2D ^13^C–^13^C DARR, ^1^H–^15^N HETCOR (**Figure 2B**), and 1D ^13^C (Supplementary Figure S1) and ^15^N CP (cross polarization)/MAS (**Figure 2B**) experiments was 13000 Hz. Typical ^1^H *π*/2 and ^13^C *π* pulses were 3.1 and 5.2 μs, respectively. The ^1^H power followed a tangent amplitude ramp (100–80%) during ^1^H–X (X = ^13^C or ^15^N) CP. The ^1^H–^13^C MELODI–HETCOR (**Figure 2A** and Supplementary Figure S2) and SIDY (Supplementary Figure S3) spectra for identification of chromophore–protein interactions were acquired at a MAS rate of 11778 Hz. Intra- and intermolecular ^1^H contacts of the bilin chromophore (Supplementary Tables S2, S5–S9) were assigned based on the corresponding QM/MM models for Pr and Pfr (deposited in Supplementary Data Sheet S1) and validated in the same manner as in Song et al. (2012). All Pfr spectra were obtained by subtraction of the corresponding Pr spectra (Song et al., 2012) from those of the Pfr/Pr (4:1) mixture used in this study with an appropriate weighting constant of ∼0.2. Acquisition and processing parameters of the MAS spectra are given in Supplementary Materials and Methods.

### Improved Structural Model as Pr

In our earlier paper (Song et al., 2012) the Pr signals were assigned with the help of a QM/MM-optimized model for the oat phyA3 sensory module based on that of *Arabidopsis* phyA with the native PΦB chromophore (Mroginski et al., 2011). Here we present an improved model for Pr with 204 atoms treated quantum-mechanically based on the interfacial ^1^H–^13^C heteronuclear correlation spectra using the PCB model corresponding to the *u*-[^13^C,^15^N]-PCB chromophore investigated experimentally (Supplementary Figures S4, S5 and Supplementary Tables S10, S11). The selected structural parameters of the PCB chromophore of the QM/MM models in both Pr and Pfr states for oat phyA3 are summarized in Supplementary Table S12.

## Author Contributions

CS, WG, JM, and JH conceived the experiments. CS, MM, WG, and JM designed the experiments. CS, MM, CL, and JK performed the experiments. CS, MM, WG, JM, and JH analyzed the data. CS and JH wrote the manuscript with extensive input from MM, WG, and JM. All the authors read and corrected the manuscript.

## Conflict of Interest Statement

The authors declare that the research was conducted in the absence of any commercial or financial relationships that could be construed as a potential conflict of interest.

## Abbreviations

BphP: bacteriophytochrome
CAT: catalytic ATPase
CBCR: cyanobacteriochrome
CD: circular dichroism
CP: cross polarization
Cph1: cyanobacterial phytochrome 1
Cph2: cyanobacterial phytochrome 2
DARR: dipolar-assisted rotary resonance
DHp: dimerization and histidine phosphoacceptor
DNP: dynamic nuclear polarization
*Dr*BphP: *Deinococcus radiodurans* BphP
GAF: c*G*MP phosphodiesterase/*a*denylyl cyclase/*F*hlA
HDX-MS: hydrogen-deuterium exchange coupled to mass spectrometry
HK: histidine kinase
MAS: magic-angle spinning
MELODI–HETCOR: medium- and long-distance heteronuclear correlation
NTE: N-terminal extension
*Pa*BphP: *Pseudomonas aeruginosa* BphP
PAS: *P*er/*A*rnt/*S*im
PCB: phycocyanobilin
Pfr: far-red-light-absorbing state
PHY: *phy*tochrome-specific
phyA: phytochrome A
PIF3: phytochrome-interacting factor 3
Pr: red-light-absorbing state
PΦB: phytochromobilin
QM/MM: quantum mechanics/molecular mechanics
SIDY: selective interface detection spectroscopy
*Xc*BphP: *Xanthomonas campestris* BphP
