# Supplementary material for: 3D Structures of Plant Phytochrome A as Pr and Pfr From Solid-State NMR: Implications for Molecular Function
Source: Front Plant Sci. 2018 Apr 24;9:498. doi: 10.3389/fpls.2018.00498 (PMC5928327; doi:10.3389/fpls.2018.00498)
Supplement: Supplementary file 2 [file Presentation_1.PDF]

Supplementary Material

Structures of Plant Phytochrome A as Pr and Pfr from Solid-State NMR: Implications for Molecular Function

Chen Song*, Maria Andrea Mroginski, Christina Lang, Jakub Kopycki, Wolfgang Gärtner, Jörg Matysik, and Jon Hughes*

* **Correspondence:** Jon Hughes: jon.hughes@uni-giessen.de; Chen Song: chen.song@uni-leipizg.de.

This pdf file includes:

Supplementary Results

Supplementary Materials and Methods

Supplementary References

Supplementary Figures

Supplementary Figure 1. Enlarged contour plots of the DARR spectra shown in Figure 1 with complete chromophore assignments.

Supplementary Figure 2. Interfacial ^{1}H–^{13}C contacts between the PCB chromophore carbons and the surrounding residues (within the region of ~4.5 Å) obtained from *u*-[^{13}C,^{15}N]-PCB oat phyA3 as Pfr.

Supplementary Figure 3. Selective observation of interfacial 2.5–6.5 Å ^{1}H contacts of the chromophore non-quaternary carbons obtained from *u*-[^{13}C,^{15}N]-PCB oat phyA3 as Pfr.

Supplementary Figure 4. Resonance reassignment of MELODI–HETCOR spectrum of oat phyA3 as Pr.

Supplementary Figure 5. Resonance reassignments of SIDY spectrum of oat phyA3 as Pr.

Supplementary Figure 6. State-related changes in ^{13}C and ^{15}N chemicals shifts of the PCB chromophore in phytochromes oat phyA3 and Cph1 and in two red/green CBCRs AnPixJg2 and NpR6102g4.

Supplementary Figure 7. State-related changes in π-conjugated ^{13}C chemical shifts for oat phyA3 and Cph1.

Supplementary Figure 8. ^{15}N Pfr signal splitting associated with the ***B***-ring nitrogen in oat phyA3 and Cph1.

Supplementary Figure 9. State-related changes in ^{13}C resonance FWHM line-widths ($\nu_{1/2}$) of the chromophore in oat phyA3 and Cph1.

Supplementary Figure 10. UV-visible absorbance spectra of the oat phyA3 preparations used in this study.

Supplementary Figure 11. Definition of the 198-atom QM region for QM/MM calculations of oat phyA3.

Supplementary Figure 12. Superimposition of the Pr chromophore pocket of oat phyA3 (see PDB file deposited in Supplementary Material) and *Arabidopsis* phyB (Burgie et al., 2014).

Supplementary Tables

Supplementary Table 1. Overview of ^{13}C and ^{15}N chemical shifts of the *u*-[^{13}C,^{15}N]-PCB chromophore in oat phyA3 as Pr and Pfr.

Supplementary Table 2. Intramolecular ^{1}H–^{13}C correlations of the PCB chromophore (between $H^{N21–N24}$ and PCB carbon atoms).

Supplementary Table 3. Experimental ^{15}N chemical shifts for the tetrapyrrole nitrogen atoms of the oat phyA3 PCB chromophore as Pfr compared with the computed values using QM/MM model in the corresponding Pfr state.

Supplementary Table 4. FWHM line-widths of ^{13}C and ^{15}N resonances ($\nu_{1/2}$) of the PCB chromophore atoms in *u*-[^{13}C,^{15}N]-PCB oat phyA3.

Supplementary Table 5. Overview of the interfacial ^{1}H contacts of the chromophore between the PCB carbon atoms and neighboring protons of the protein residues ($^{1}H^{residue}$–$^{13}C^{PCB}$).

Supplementary Table 6. The intermolecular ^{13}C chromophore contacts of the protein residues (cut-off distance ~5.0 Å).

Supplementary Table 7. Selective observation of interfacial 2.5–6.5 Å ^{1}H contacts of the non-quaternary PCB carbon atoms.

Supplementary Table 8. The interfacial ^{13}C chromophore contacts of the protein residues.

Supplementary Table 9. The intermolecular ^{13}C chromophore contacts of the protein residues.

Supplementary Table 10. Reassigning the interfacial ^{1}H contacts of the PCB carbon atoms in the Pr state.

Supplementary Table 11. Reassigning the interfacial 2.5–6.5 Å ^{1}H contacts of the non-quaternary PCB carbon atoms as Pr.

Supplementary Table 12. Selected structural parameters of the PCB chromophore of the QM/MM models in both Pr and Pfr states for oat phyA3.

Supplementary Table 13. Amino acid conservation of oat phyA3 (*As*.phyA3) and *Arabidopsis* phyB (*At*.phyB) in the direct bilin environment.

Pr- and Pfr-state structural models for oat phyA–PCB

SUPPLEMENTARY RESULTS

Chemical shift assignments of the chromophore in its Pfr state. Using a similar strategy to that previously employed for Pr (Song et al., 2012), we derived complete and unambiguous ^{13}C and ^{15}N assignments of the PCB chromophore as Pfr from a series of 2D homo- and heteronuclear correlation experiments with the sensory module of oat phyA3 carrying *u*-[^{13}C,^{15}N]-labeled PCB chromophore. As a first step, we acquired ^{13}C–^{13}C DARR spectra with two mixing times of 5 and 50 ms (Figure 1, red and purple, respectively; enlarged view with 1D traces on both dimensions in Supplementary Figure S1) which were found to be most efficient in detecting direct ^{13}C–^{13}C connectivities (5 ms) and medium-range carbon atom pairs (50 ms) such as C1–C3 or C2–C4 (see Figure 3 for numbering) of the PCB chromophore within its binding pocket (Rohmer et al., 2008; Song et al., 2012). The unresolved set of C4 and C6 as well as C18^1 and C18^2 of the ***D***-ring ethyl group in our previous DARR study (Rohmer et al., 2008) can now be assigned unambiguously, thus completing assignment of the bilin carbon atoms in the Pfr state (Supplementary Table S1). The assignment of C4 (δ^C = 152.7 ppm) and C6 (155.7 ppm) derived from the well-defined correlation network connecting C3–C4–C5–C6–C7 (indicated by solid lines, Figure 1). Correlations involving the C18-ethyl group (C18–C18^1–C18^2, solid lines) are clearly present using a short proton mixing time of 5 ms, allowing assignment of the peaks at 16.0 and 13.3 ppm to C18^1 and C18^2, respectively. The assignments were validated by the correlations between non-bonded ^{13}C pairs such as C18^1–C19 and C18–C18^2 in the spectrum recorded with a mixing time of 50 ms (Supplementary Figure S1, purple). Further, the DARR spectra allowed unambiguous assignment of the propionate side chains attached to rings ***B*** and ***C***, the latter [C12^1 (20.6 ppm)–C12^2 (38.7 ppm)–C12^3 (174.8 ppm), Figure 1] showing none of the doubling/splitting observed in the Pr state (Song et al., 2012). No signal doublings were seen for the ***A***-ring pyrrolic carbons either (Supplementary Table S1). Using a mixing time of 50 ms, a long-distance correlation network involving C11$\cdots$C12$^2\cdots$C13$^1\cdots$C17$\cdots$C18^2 is visible (Supplementary Figure S1, purple). According to our Pfr model (see PDB file in Supplementary Material), the internuclear distances between these carbon pairs are 3.7–3.8 Å, roughly the maximum effective detection range at this mixing time with our current sensitivity. Most δ^C values of the bilin carbon atoms derived from the DARR data match well with those previously reported ($\leq$ 0.4 ppm; Rohmer et al., 2008), while several other pyrrolic carbon atoms like C2, C7, C9, C14, C15, and C18 exhibited a 0.5–0.8 ppm shift (Supplementary Table S1), probably deriving from subtle sample differences.

We next acquired two ^{1}H–^{13}C MELODI–HETCOR spectra with LG–CP contact times of 500 and 900 μs (Figure 2A, red and purple, respectively) to establish the ^{1}H intramolecular contacts of the chromophore carbons. This experiment permitted unambiguous ^{1}H assignment of the one-bond ^{15}N-coupled protons of the tetrapyrrole nitrogen ($H^{N21–N24}$). With a short LG–CP mixing period of 500 μs (cut-off distance set to be 2.8 Å; Li and Hong, 2011), the correlation spectrum (Figure 2A, red) revealed four clear ^{1}H resonances at δ^{H} = 9.0, 10.3, 11.3, and 11.8 ppm for Pfr. The NH proton resonating upfield at 9.0 ppm was assigned to H^{N24} via its contacts with C19 (169.5 ppm), C16 (150.3 ppm), and C18 (140.7 ppm) at the same ring. No contact was detected between these ***D***-ring carbons and the other NH protons of rings ***A***–***C***, even in the spectrum acquired with an LG–CP contact time of 900 μs (Figure 2A, purple). During this transfer period, an effective range of ~3.5 Å was set for detection of heteronuclear polarization transfers (van Rossum et al., 2002; Song et al., 2012). This is also consistent with the intramolecular distance restraints extracted from the optimized Pfr model, in which the ***D***-ring carbons are at least 3.8 Å distant from the other three NH protons, as such beyond our maximal detection range. Moreover, in the Pr state, the H^{N24} correlated with the ring ***C*** at C13, $C13^{1}$, and C14, although neither was resolved in the Pfr state (Supplementary Table S2). Also, a DARR correlation connecting $C13^{1}$ (12.8 ppm) of ring ***C*** and C17 (138.5 ppm) of ring ***D*** (Supplementary Figure S1) is only seen in Pfr. In the most downfield ^{1}H slice at 11.8 ppm, the NH proton exhibited three strong correlations to C1 (182.1 ppm) and C4 (172.5 ppm) of ring ***A*** as well as C5 (86.7 ppm) and hence was assigned to the H^{N21} of ring ***A*** (Figure 2A, red). The integrated intensity ratio of the two ***A***-ring contacts of H^{N21} (H^{N21}–C1 vs. H^{N21}–C4) is ~45:55, implying that the two carbons are roughly equidistant from the proton (van Rossum et al., 2002), consistent with the distance restraints extracted from the Pfr model, thereby confirming the assignment. Two remaining unassigned ^{1}H signals at 11.3 and 10.3 ppm in the Pfr spectra (Figure 2A) correspond to the inner rings ***B*** and ***C***, both containing the correlations from C9 of ring ***B*** and C11 (130.1 ppm) of ring ***C***. Assignment is nevertheless possible on the basis of their different intensities. For example, in the spectrum acquired with a 900-μs LG−CP mixing time (Figure 2A, purple), the proton at 11.3 ppm yielded strong and weak correlations with C9 and C11, respectively, whereas the situation was reversed for the proton at 10.3 ppm. This leads to unequivocal assignment of the two ^{1}H signals at 11.3 and 10.3 ppm to H^{N22} and H^{N23}, respectively (these two NH protons in Pr were indistinguishable in the ^{1}H dimension at 10.7 ppm; Supplementary Table S2). The assignment of all four NH protons is thereby complete. State-related δ^{H} changes ($\Delta\delta^{H}$) were seen: −0.3, +0.6, −0.4 and +0.8 ppm as Pfr minus Pr for H^{N21} to H^{N24}, respectively.

We then used a ^{1}H–^{15}N HETCOR experiment to correlate tetrapyrrole nitrogens to the assigned NH protons via their direct connectivities (Figure 2B). Each ^{15}N atom exhibits a single robust correlation to its bonded proton, and is therefore assigned directly (summarized in Supplementary Table S1). N21 of ring ***A*** is located at 159.0 ppm, N23 and N22 of the two inner rings ***C*** and ***B*** are further upfield at 157.3 and 140.7 ppm, respectively, and N24 of ring ***D*** is most upfield at 136.9 ppm (Supplementary Table S1). These measured ^{15}N resonances were closely matched by those computed using a QM/MM approach with the QM subsystem consisting of 198 atoms ($\delta^{N21-N24}$ = 165.3, 140.7, 154.1, and 138.0 ppm, respectively; Supplementary Table S3). It should also be noted that the recent ^{15}N DNP enhanced MAS NMR characterization of the Pr state in Cph1 finalized the assignment of the Pr resonances (Stöppler et al., 2016), in which the previous tentative assignments of N22 and N23 were interchanged (Rohmer et al., 2008). This thus forms a solid basis on calculating the state-induced δ^{N} changes of the chromophore in oat phyA3 (see below).

Changes in ^{13}C and ^{15}N shifts of the chromophore during Pr → Pfr photoconversion. The state-induced δ^{C} and δ^{N} changes ($\Delta\delta$) of the chromophore are illustrated in Figure 3A. It is clear from the $\Delta\delta$ pattern that the ***C***- and ***D***-ring regions of the chromophore (for example, the pyrrolic quaternary carbons as well as the ***C***-ring propionate side chain) are predominately affected upon photoconversion to Pfr. In particular, the C13 and C16–C18 signals are shifted by at least 4.0 ppm relative to Pr (Supplementary Table S1), consistent with a ***D***-ring photoflip and in harmony with the DARR contact between $C13^{1}$ and C17 seen only in Pfr (see above). In addition, $\Delta\delta^{C}$ at the ***C***-ring propionate (all carbon atoms displaced upfield by at least 1.9 ppm) as well as a $\Delta\delta^{N}$ of +4.2 ppm for N24 reflect structural rearrangement of the binding site during photoconversion. Further details are provided by the interfacial ^{1}H contacts of the chromophore atoms (see **Discussion**). Moreover, whereas in the region of rings ***A***–***B*** 12 of 16 carbon atoms show $|\Delta\delta^{C}| \leq 1.3$ ppm upon photoconversion, $\Delta\delta^{C}$ values of −1.9, +1.3, and −1.5 ppm are seen for the ***A***-ring linkage to the protein at C3, $C3^{1}$, and $C3^{2}$, respectively (Supplementary Table S1). Figure 3A also implies state-related δ^{C} changes in the conjugated π-system of the chromophore. Unlike the nearly complete alternating $\Delta\delta^{C}$ pattern for the conjugated chains from C4 to C9 of rings ***A***–***B*** and from C16 to C19 of ring ***D*** (alternating red and blue circles), all four ^{13}C Pr signals of the ***C***-ring pyrrolic carbons are displaced downfield (red circles).

Changes in the chromophore ^{13}C and ^{15}N line-widths upon Pr → Pfr photoconversion. Our earlier ^{1}H and ^{13}C MAS NMR studies revealed Pr-state structural heterogeneity of both oat phyA3 and Cph1, some

carbons in/around the ***A***-ring region, the ***C***-ring propionate side chain and several protein residues nearby showing at least two conformations (Song et al., 2013). For the Pfr chromophore, however, we found no evidence of structural heterogeneity on this scale, only a single set of δ^{C} values being apparent in the two DARR spectra (Supplementary Table S1). In addition, intermolecular ^{1}H contacts of the Pfr chromophore imply no alternative side chain conformations and/or protonation shifts for the nearby residues in the pocket (see below). However, we noticed that in the 1D ^{15}N spectrum (Figure 2B, projection onto the ^{15}N axis), the maximum assigned to N22 at ~141 ppm is asymmetrical and significantly broader than the other three maxima. Band-fitting analysis using a Voigt profile (a mixed Lorentzian–Gaussian ratio of 1:1; Bruce et al., 2000) revealed a major component at 140.7 ppm and minor downfield at ~142.1 ppm with an intensity ratio ~0.7:0.3 (see also Supplementary Figure S8).

Besides the global structural homogeneity on both ^{1}H and ^{13}C NMR time scales in the Pfr state, it is obvious that the line-widths of the ^{13}C and ^{15}N signals of the chromophore in the two states are distinct (expressed as full-width at half-maximum $\nu_{1/2}$, summarized in Supplementary Table S4). As shown in Figure 3B, almost all Pfr signals were sharper in Pfr than in Pr (blue circles). Pfr line narrowing is most dramatic for the ***A***-ring linkage to the protein at C3, C3^{1} and C3^{2}, for example, the ^{13}C line-width of C3^{1} decreases from 763.6 Hz as Pr to 394.5 Hz as Pfr ($\Delta\nu_{1/2} = -369.1$ Hz as Pfr minus Pr). Major narrowing is also seen for C3^{2} (617.0 Hz to 360.8 Hz) and for C3 (724.2 Hz to 589.4 Hz). A number of ^{13}C signals of the peripheral carbons of rings ***B***–***D*** also narrow, *e.g.*, C8^{1}, C12^{2}, C17^{1}, and C18^{1}. The observed ^{13}C and ^{15}N line-narrowing in the Pfr chromophore does not result from an increase in spin-spin relaxation time (Siemer et al., 2012) but rather from a decrease in structural heterogeneity of the surrounding protein relative to Pr.

Interfacial ^{1}H contacts of the chromophore in its Pfr state. We have previously probed the binding pocket of the oat phyA3 chromophore in the Pr state by detecting interfacial 3.5–6.0 Å contacts between uniformly ^{13}C-labeled chromophore and unlabeled moieties ($^{1}H^{residue}$–$^{13}C^{PCB}$) using two long-range heteronuclear correlation methods, MELODI–HETCOR and SIDY (Song et al., 2012). We employed similar methods here for Pfr to allow a direct comparison of the chromophore–protein interactions in each state (Supplementary Figures S2, S3). The MELODI–HETCOR and SIDY Pfr contacts of oat phyA3 were identified with the help of QM/MM-optimized Pfr model (deposited in Supplementary Material). The overall ^{1}H assignment strategy has been described in detail in the previous Pr study (Song et al., 2012). For instance, in the Pfr model the highly-conserved H372, forms a strong hydrogen bond with

the ***C***-ring carboxylate moiety ($C12^3$) via its imidazole ring at Nε2 (an O···N distance of 3.2 Å). The model also reveals two more protons of this imidazole, Hδ2 and Hε1 within the ~4.5-Å effective detection range of $C12^3$ by choosing an LG–CP mixing time of 2.3 ms (Song et al., 2012). Indeed, in the MELODI–HETCOR slice corresponding to $C12^3$ (δ^C = 174.8 ppm; Supplementary Figure S2), these three interfacial transfer events from H372 imidazole are all present above 9 ppm in the ^{1}H dimension. The most intense signal (δ^H = 12.8 ppm) was assigned provisionally to the hydrogen-bonding interaction ($C12^3$-O···H372-Hε2), because the buildup rate of a C-H correlation relies strongly on the shortest possible distance for interfacial transfer (van Rossum et al., 2002). As shown in Supplementary Figure S2, this imidazole proton also exhibits two correlations with $C12^2$ (δ^C = 38.7 ppm) and $C13^1$ (12.8 ppm). Considering the maximum detection distance of ~4.5 Å, Hε2 is the only candidate which can be identified with intermolecular transfers involving both carbons (the shortest distances of 3.8 and 4.4 Å for $C12^2$···Hε2 and $C13^1$···Hε2, respectively; Supplementary Tables S5, S6). This proton resonating at 12.8 ppm was thus assigned to Hε2. Two remaining ^{1}H signals at 9.9 and 11.5 ppm in the downfield region of the $C12^3$ slice are thus Hδ2 and Hε1 of the imidazole. However, the MELODI–HETCOR data do not permit an unambiguous assignment of this feature, because neither proton correlates with other chromophore carbons, consistent with the Pfr model in which $C12^3$ is found to be the only carbon of the chromophore within the 4.5 Å detection range of those two imidazole protons. However, this ambiguity was readily resolved by the SIDY experiment which is more effective for longer interfacial ^{1}H–^{13}C polarization transfer, *e.g.*, up to 6.0 Å (Kiihne et al., 2005; Song et al., 2012). Each of the SIDY slices matching $C12^2$ (δ^C = 38.7 ppm) and $C13^1$ (12.8 ppm) exhibited a single correlation to one member of the two ^{1}H slices at 9.9 and 11.5 ppm (Supplementary Figure S3). $C12^2$-connected ^{1}H signal at 11.5 ppm could thus be assigned to Hδ2 since a contribution from Hε1 is very unlikely given an intermolecular distance of 7.3 Å, while its interaction with Hδ2 is well within the detection range ($C12^2$··· H372-Hδ2 = 5.4 Å; Supplementary Table S8). We thus attribute the remaining ^{1}H signal at 9.9 ppm in the $C13^1$ slice to the magnetization transfer from H372-Hε1 which are 5.1 Å apart based on the Pfr model.

The side chain of another conserved histidine residue, H323, was found to be in close contact with the pyrrolic carbons of the two inners rings as well as their propionates in Pr. The corresponding ^{1}H shifts nicely distinguished cationic and neutral τ tautomers of the H323 imidazole in the two Pr isoforms (Song et al., 2012). In the two interfacial correlation spectra for Pfr (Supplementary Figures S2, S3),

however, the apparent absence of ^{1}H signals characteristic of cationic imidazolium at the large downfield region (*e.g.*, Hδ1 signal appeared at 16.9 ppm in Pr; Supplementary Table S9) indicates that the side chain is neutral. In addition, the Pfr data do not support the coexistence of neutral τ (Nε2-H) and π (Nδ1-H) tautomers. Were two tautomeric states of the histidine to coexist in Pfr, numerous ^{1}H imidazole signals would be expected in the downfield region: we identified only two nonequivalent ^{1}H slices at 12.3 and 13.5 ppm, however (Supplementary Table S9). The former could be assigned to Hε1 by virtue of its weak connection with C8^1 in the SIDY spectrum (Supplementary Figure S3) which are 6.1 Å apart in the Pfr model, close to the 6.0-Å detection limit. Although contributions from other imidazole protons such as Hδ1 and Hε2 are possible, both are located too far away from this carbon (> 7 Å) for allowing magnetization transfer. This assignment was confirmed by the MELODI–HETCOR spectrum shown in Supplementary Figure S2 that reveals four correlations at the expected ^{1}H position with C8, C10, C11, and C12^1. These ^{1}H–^{13}C distance constraints involving Hε1 match precisely the interfacial contacts derived from the Pfr model. The agreement implies that an Nε2-protonated H323 as revealed by the model would be expected in this situation which otherwise introduces a significant effect on the ring orientation of its imidazole with respect to the ***B***–***C*** plane (Mroginski et al., 2009). We thus assign the remaining imidazole ^{1}H signal at 13.5 ppm resolved only in the SIDY spectrum to Hε2 which is observed to correlate with C12^1 and C12^2 (Supplementary Figure S3). These data again match our Pfr model in which Hε2 has no chromophore partners within the 4.5-Å detection range set by MELODI–HETCOR (Supplementary Figure S2), but two SIDY observable ^{13}C contacts at 5.3 and 4.0 Å for C12^1 and C12^2, respectively (Supplementary Table S8). We thus conclude that H323 exists in the Nε2-protonated τ tautomeric form in the Pfr state, well-supported by both our earlier MAS NMR data on Cph1 (Song et al., 2011) and a very recent investigation combining pH-dependent resonance Raman spectroscopy and QM/MM calculations on the same protein (Velazquez Escobar et al., 2017).

Conformational changes in residues surrounding the chromophore. Unlike Pr, we detected only local Pfr conformational heterogeneity in the chromophore (N22 in ring ***B***) and none in its immediate protein environment (*e.g.*, ~6.0 Å), indicative of a rigid pocket. The change in rigidity around the chromophore between Pr and Pfr is also evident in the NTE. The SIDY spectra revealed a single ^{1}H contact (with C2 of ring ***A***) in Pr, yet no less than 15 contacts (mainly with the ethylidene and methyl side chains of ring ***A*** and C5) in Pfr likely to be associated with the NTE (Supplementary Table S7). For example, C3 of ring ***A*** (with which the thioether linkage to the protein is made) in Pfr exhibited a strong correlation to a

proton associated with a neighboring aromatic side chain at 10.1 ppm (Supplementary Figure S3). As no appropriate candidates in the GAF domain and the tongue region exist within ~6.0 Å of C3, we tentatively ascribed this ^{1}H contact to the neighboring NTE region, perhaps the ring proton of Y69. This proton possibly also interacts with C3^{2}, whereas C3^{1} might contact another ring proton of Y69 resonating at 9.4 ppm (Supplementary Tables S7, S8). In the Pfr-state MELODI–HETCOR spectrum (Supplementary Figure S2), we also observed 16 correlations involving the NTE residues, again in contrast to Pr in which no such contacts were detected (Supplementary Table S6).

Detailed analysis of the tongue region by MELODI–HETCOR revealed a number of correlations in Pr connecting M549–P551, P540, and S541 of the tongue to the ***A***-ring carbons (Supplementary Table S6). Most of these interactions were preserved in the corresponding SIDY spectrum (Supplementary Figure S3, Supplementary Table S8). Most importantly, these residues, together with R552 seen in the SIDY spectrum, are in close contact with the ring ***A*** in Pr, whereas in the two corresponding Pfr spectra, only M549 of the above-mentioned tongue residues was detected (Supplementary Table S9). These differences are supported by the structural models, in which these residues in Pfr are too far away to interact with the chromophore. Besides M549, the Pfr chromophore is correlated via its ***D***-ring carbons with the side chain protons (Hβ and Hγ1) of S554 from the conserved PRXSF$^{551–555}$ motif of the tongue (Figure 4A, B). R548, another tongue residue seen by SIDY only in the Pfr state, exhibited two contacts to the ring ***A*** at C2 and C2^{1} via its Hβ (at distances of 5.8 and 5.4 Å, respectively; Supplementary Table S8). Conversely, in the case of Pr the nearest C-H spin pairs involving the side chain protons of R548 and the ***A***-ring carbons (that is, C3$^{2}\cdots$Hβ) are at least 7.5 Å apart. Moreover, although M549 is seen in both Pr and Pfr, no correlations originated from the same C-H pairs were observed (Supplementary Table S9).

^{1}H contacts of the PCB chromophore observed in the two interfacial correlation spectra for Pr and Pfr are given in Supplementary Table S9. The amino acids with at least one observable ^{1}H contact in both states are shown in Figure 5. The light-driven ^{1}H changes ($\Delta\delta^{H}$) are highlighted by red and blue spheres representing down- and up-field shifts, respectively. The most prominent ^{1}H changes are detected for R317 (Hη1 and Hη2) and Y326 (Hη). As seen in Figure 5, the ***B***-ring propionate carboxylate oxygens pair with the guanidinium moiety (Nη1/Nη2) of R317 in Pr. The observed ^{1}H changes at these positions ($\Delta\delta^{H}$ = +1.3 and +1.7 ppm) are associated with the partner swap of the ***B***-ring propionate, as evidenced by the 'Pfr-only' contacts connecting the ***B***-ring propionate to the guanidinium moiety of R287 at Nε

and Nη2 (Supplementary Table S9). This is associated with a shift of the two inner rings implied by the changes in ^{1}H contacts involving I273 and P274 (Supplementary Table S9).

As also shown in Figure 5, Y241, Y268 and Y326 form part of the hydrophobic subpocket around ring ***D***. The ^{1}H interfacial contacts of these tyrosines to the chromophore carbons are radically different in Pfr from those in Pr (Supplementary Table S9), indicating structural rearrangement of their phenolic side chains. As an example, Hδ1 and Hε1 of Y241 in Pr were seen solely by the ***D***-ring carbons like C17^{1} and C18^{1}, whereas in Pfr these two carbons interact with Hδ2 and Hε2 on the other side of the ring. Without rotamerization of the Y241 side chain in Pfr, these two ring protons would be too distant to contact the ***D***-ring methyls. Also, a −0.6 ppm $\Delta\delta^{H}$ observed for Hβ provides additional qualitative support for a change in conformation of its phenolic side chain associated with photoconversion. Indeed, our models (see PDB files in Supplementary Material) suggest a t,g^{+} rotamer ($\chi_1 = 191°$, $\chi_2 = 61°$) in Pr and a g^{-},t rotamer ($\chi_1 = 304°$, $\chi_2 = 333°$) in Pfr (see Supplementary Materials and Methods for rotamer definitions), associated with a reduction of ~1.5 Å in the (χ_1,χ_2)-dependent Cε2–Nα internuclear distance. Similarly, large side-chain rearrangements around ring ***D*** were observed for Y268 which, in Pr, places its phenolic ring close to the ***D***-ring methyl and ethyl groups, as evidenced by a number of well-resolved correlations involving C17^{1}, C18^{1} and C18^{2} and the ring protons of Y268 (Hδ1, Hε1, and Hε2). In the Pfr correlation spectra, however, a single ^{1}H contact of Y268 to the ***D***-ring carbons could be identified (C18···Y268-Hε2; Supplementary Table S9), this ring proton exhibiting a large δ^{H} shift of +0.8 ppm. Both Pfr correlation spectra for Y326 on the opposite α-face of the ***B***–***C*** plane connect the phenolic hydroxyl group (Oη) to C15 and C16 (Supplementary Table S9), indicating proximity to ring ***D*** (Figure 4C). The global δ^{H} changes for its phenolic ring and a +1.7-ppm $\Delta\delta^{H}$ for Hη in particular would be consistent with a hydrogen bond to the ***D***-ring nitrogen (N24). QM/MM calculations contradicted this, however, the Pfr model showing a Y326-Oη···N24 separation of > 4 Å, too large for a hydrogen bond. Indeed, a contact to C19 was not detected although this would clearly be expected in the case of a hydrogen bond. Further prominent δ^{H} changes of > 1.0 ppm on photoconversion to Pfr were detected for the imidazole rings of H372 and the neighboring H323 ($\Delta\delta^{H}$ for Hε2 of −1.4 and −1.2 ppm for H372 and H323, respectively; Supplementary Table S9).

SUPPLEMENTARY MATERIALS AND METHODS

Sample preparation. The holophytochrome sample [the complete sensory module (residues 1–595) of *Avena sativa* (oat) phytochrome A3 expressed in yeast and *in vitro* assembled with *u*-^{13}C,^{15}N-PCB chromophore] used in our earlier MAS NMR study (Song et al., 2012) was thawed, drawn into a Hamilton syringe, then passed slowly through a ~400 µm internal diameter glass capillary irradiated with red light from 660 nm (20 nm FWHM) LEDs to generate the Pfr/Pr 8/2 photoequilibrium mixture. The flow-through was collected directly in the MAS rotor and flash frozen in liquid N_2. No additional illumination was applied to the sample during data collection.

Construction of structural model for oat phyA3 Pfr. The 3D structural model of the oat (*Avena sativa*) phyA3 PCB adduct in the Pfr state was constructed in four steps: (*i*) Generation of a preliminary model based on the oat sequence and published crystal structures, (*ii*) crude QM/MM geometry optimization of the chromophore-binding pocket using a small QM region, (*iii*) model refinement guided by the assignment of NMR-determined chromophore interfacial ^{1}H contacts, and (*iv*) QM/MM geometry optimization of the chromophore-binding pocket using a larger QM region.

i. A preliminary structural model for the sensory module of oat phyA3 in the Pfr state was generated with the help of MODELLER (salilab.org/modeller; Šali and Blundell, 1993) and UCSF Chimera (www.rbvi.ucsf.edu/chimera; Pettersen et al., 2004). An initial structure-based amino acid sequence alignment was based on the sensory modules of Cph1 (2VEA; Essen et al., 2008), the ‘bathy’-type bacteriophytochrome *Pa*BphP (3C2W; Yang et al., 2008) and oat phyA3, omitting the first 62 residues of the plant sequence for which no structural data are available.

ii. The structural model of the protein was first solvated in a hexagonal water box containing ~90000 molecules as well as 60 Na^{+} ions and 55 Cl^{-} ions to neutralize the system. Water molecules with oxygen less than 2.8 Å from non-hydrogen protein atoms were subsequently removed. The positions of all atoms in the system were then energy-minimized with only the PCB chromophore and Cα atoms of the protein backbone fixed in space. For the energy minimization as well for the subsequent molecular dynamics steps, water molecules were modeled using the TIP3P approach (Jorgensen et al., 1983), while the protein atoms, including the PCB chromophore, were described by the CHARMM22 force field (MacKerell et al., 2001; Kaminski and Mroginski, 2010). Subsequently, the entire system (except for chromophore and Cα-atoms) was thermally equilibrated at 300 K in an isothermal-isobaric ensemble for 40 ns by means of classical MD simulations under periodic boundary conditions. For this purpose, the Langevin-Piston and Nose-

Hoover methods were employed using damping factors of 5 ps and 50 fs for temperature and pressure, respectively (Feller et al.,1995). To satisfy a time step of 2 fs, the SHAKE algorithm (Ryckaert et al., 1977) was used to constrain all bond lengths between heavy and hydrogen atoms. Non-bonded electrostatic interactions were treated by the particle-mesh-Ewald method (Darden et al., 1993). For the van der Waals interactions, a switching function was used with a cut-off at 12 Å. The solvation and initial energy minimization steps were performed with the CHARMM36 (Brooks et al., 2009). The MD simulations used NAMD V2.10 (Phillips et al., 2005). The structure of the protein solvated in a thermally-equilibrated aqueous solution was used as input for the subsequent QM/MM calculation.

The geometry optimization of the chromophore-binding site was performed at a QM/MM level using the limited memory quasi-Newton L-BFGS algorithm working with delocalized internal coordinates (Billeter et al., 2000). This algorithm is implemented in the ChemShell modular program package linking Turbomole and DL-Poly engine for the specific quantum mechanical and molecular mechanical calculations (Metz et al., 2014). In the QM/MM setup, the molecule was partitioned into three regions. The central QM region of the initial Pfr model included 85 atoms to be treated quantum mechanically. These included the PCB chromophore and the side chain of C322 covalently bound to the chromophore. Atoms in the QM region were treated at the B3LYP/6-31G* level of theory. The link atom approach was employed to saturate the dangling bond of C322 (Bakowies and Thiel, 1996). The second mobile MM shell region included all residues surrounding the QM region less than 20 Å distant from N23 of the chromophore. Atoms in the mobile MM shell were allowed to relax during energy minimization following the laws of molecular mechanics. Bonded and non-bonded interactions within these atoms were described with the CHARMM22 force field. Finally, the static MM shell region included all residues 20–40 Å from the central N23. Atoms belonging to this were modeled simply as fixed point-charges with values taken from the CHARMM22 force field. In order to reduce computational cost, all residues > 40 Å from N23 were removed from the model since they do not contribute significantly to the electrostatic energy in the QM region. Furthermore, the boundary between the QM region and the MM mobile shell was treated using the electrostatic-embedding approach combined with the charge-shifted scheme (Bakowies and Thiel, 1996).

iii. The resulting Pfr QM/MM model was then used as a guide in assigning chromophore interfacial ^{1}H contacts experimentally-determined in MELODI–HETCOR and SIDY correlation methods.

As mentioned above, the original structure was built on the basis of the BV-containing bacteriophytochrome *Pa*BphP 3C2W Pfr structure, consequently with an α-facial disposition for its ring ***D*** (***D***-α_f) relative to the rings ***B*** and ***C***. Such a ***D***-α_f disposition proved inconsistent with many ^{1}H contacts of the chromophore resolved in the two correlation spectra, in particular for ***D***-ring carbons. We found, however, that a ***D***-β_f disposition similar to that proposed for Cph1 Pfr (Song et al., 2011) allowed most ^{1}H contacts of the ***D***-ring carbons to be assigned straightforwardly. The modified model also involved some minor side chain rearrangements of the protein residues near the chromophore. For example, a SIDY contact involving C8^1 and R317-Hη2 is well resolved, (R317-Hη2 also contacting C8^2 and C8^3 in the MELODI–HETCOR spectrum) but inconsistent with the original model which predicted a 6.3 Å separation, beyond the SIDY detection limit of ~6.0 Å. Similarly for R548 of the tongue, two ^{1}H contacts of the ***A***-ring carbons (C2/C2^1···R548-Hβ) resolved in the SIDY spectrum were inconsistent with the original model that predicted 6.8 and 6.3 Å for the C2···Hβ and C2^1···Hβ separations, respectively). The side chain conformation of this arginine residue was thus modified to be closer to the ring ***A***. Also for C2 and C2^1, the ^{1}H SIDY signal located at 9.7 ppm was attributed to the magnetization transfer from T271-Hγ1, the assignment is in accordance with the observation of intermolecular transfer to the adjacent carbon (C1) in the MELODI–HETCOR spectrum, however, the C1···T271-Hγ1 pair was predicted to be 5.3 Å apart in the original model. It is very unlikely that an intense signal can be derived from such a long intermolecular transfer event. This distance was reduced to 4.7 Å in the modified model (and further to 4.5 Å in the final QM/MM-optimized model), feasible for the C1···T271-Hγ1 heteronuclear polarization transfer. In summary, a total of 220 interfacial ^{1}H^{residue}–^{13}C^{PCB} Pfr contacts (159 resolved in MELODI–HETCOR and 120 in SIDY, 59 resolved in both spectra) were used as constraints in a final model refinement using QM/MM methods.

iv. The NMR-refined structural model of the chromophore-binding pocket in (*iii*) was finally energy-minimized using once again the QM/MM method described in (*ii*) except that the QM region was drastically enhanced to 198 atoms to include not only the PCB chromophore and the C322 side chain but also D272, I273, the side chains of Y241, F243, Y268[1], H323, Y326 and

---

[1]Two different conformations of its phenolic ring (roughly perpendicular and parallel to the chromophore plain) were used as the starting point for QM/MM calculations. These yielded similar optimized structures as shown in Figure 5B.

S554 as well as three water molecules participating in hydrogen-bonding interactions with the chromophore (Supplementary Figure S11). Selected structural parameters such as bond lengths, bond angles, and dihedral angles at the methine bridges of the PCB chromophore in the QM/MM-optimized models of oat phyA3 as both Pr and Pfr are given in Supplementary Table S12.

QM/MM computation of NMR chemical shifts. The NMR shielding tensors of all 198 atoms in the QM region of the QM/MM optimized structure were computed using the GIAO method (Ditchfield, 1976) at the same DFT level as employed for the energy minimization. These computations were performed with the Gaussian 09 program (Frisch et al., 2016). The ^{15}N NMR chemical shifts were computed using ammonia as reference, with an isotropic absolute shielding constant of 254.97 ppm (Supplementary Table S3).

MAS NMR data collection. All NMR data were acquired on a Bruker AV-750 WB spectrometer (Rheinstetten, Germany) operating at Larmor frequencies of 750.13 MHz for ^{1}H, 188.62 MHz for ^{13}C, and 76.01 MHz for ^{15}N using a 4 mm triple resonance MAS DVT probe. In this study, 15 mg (67 μL) of holophytochrome at photoequilibrium (4:1 Pfr/Pr ratio) in red light was loaded into a 4 mm zirconia MAS rotor and frozen in liquid N_2. In the magnet, the sample was cooled to −40 ℃ by a flow of dry N_2 of 1200 L/*h*. The MAS rate of 2D ^{13}C–^{13}C DARR (Figure 1), ^{1}H–^{15}N HETCOR (Figure 2B), and 1D ^{13}C (Supplementary Figure S1) and ^{15}N CP/MAS (Figure 2B) experiments was maintained at 13000 ± 3 Hz by a pneumatic controller. Typical ^{1}H $\pi/2$ and ^{13}C π pulses were 3.1 and 5.2 μs, respectively. For DARR, a ^{1}H r.f. field strength was ~80 kHz (corresponding to 73 watts) CW during the proton mixing and ~43 kHz (21 watts) TPPM (two-pulse phase modulation) during acquisition. For the windowed phase-modulated Lee–Goldburg (*w*PMLG) homonuclear decoupling scheme used in both MELODI–HETCOR and SIDY, a ^{1}H r.f. field strength of ~78 kHz (70 watts) was employed. And a ^{1}H r.f. field strength of ~80 kHz (73 watts) for the TPPM decoupling during acquisition in all heteronuclear correlation experiments. The ^{1}H power followed a tangent amplitude ramp (100–80%) during ^{1}H–X (X = ^{13}C or ^{15}N) CP. The ^{1}H–^{13}C MELODI–HETCOR (Figures 2A, 4B, Supplementary Figure S2,) and SIDY (Figure 4A, Supplementary Figure S3) correlation spectra for identification of chromophore–protein interactions were acquired at a MAS rate of 11778 ± 3 Hz which yielded optimal spectral resolution with a ^{1}H r.f. field of ~80 kHz and ^{1}H r.f. offset frequency of −6000 Hz (Song et al., 2011, 2012). The DARR data were collected with 1434 complex t_2 and 120 real t_1 points with 2048 scans and with an 8-ms evolution in the indirect dimension. A recycle delay of 1.5 s was used. The overall experimental

time here was 102 h. Other acquisition and processing parameters of the MELODI–HETCOR were as follows: 3072 scans with a recycle delay of 1.2 s for each t_1 point, and a total of 128 points combined in the indirect dimension, leading to an experimental time of 131 h. The t_1 data were recorded in an off-resonance manner and linear predicted by 32 points using 40 LP coefficient. A 90° shifted squared sine bell window function was applied, and zero-filled to 2048 points prior to Fourier transformation. Other acquisition and processing parameters of the SIDY were as follows: 2664 scans with a recycle delay of 1.2 s for each t_1 point, and a total of 128 points combined in the indirect dimension, leading to an experimental time of 113 h. The t_1 data were recorded in an off-resonance manner and linear predicted by 32 points using 40 LP coefficient. A 90° shifted squared sine bell window function was applied, and zero-filled to 2048 points prior to Fourier transformation. The $^{1}H–^{15}N$ HETCOR spectrum (Figure 2B) was acquired with a recycle delay of 1.5 s. An optimized LG–CP contact time of 1.54 ms was used for tracing direct $^{1}H–^{15}N$ connectivities. The spectrum was recorded with 1536 complex t_2 and 128 real t_1 points with 2560 scans, leading to an experimental time of 132 h. Prior to Fourier transformation, the data were zero-filled to 4096 points and an exponential apodization of 50 Hz was applied. The 1D ^{13}C (Supplementary Figure S1) and ^{15}N MAS spectra (Figure 2B) were recorded with 8 k and 32 k scans, respectively. A recycle delay of 1.5 s and a CP contact time of 2.048 ms were used in both cases. A Lorentzian apodization function with line broadening factors of 10 and 25 Hz was applied to ^{13}C and ^{15}N data processing, respectively. The total experimental time for the Pfr experiments was thereby 478 hours. No detectable changes occurred in the 1D ^{13}C MAS spectrum recorded with 2k scans prior to and after each round of 2D acquisitions. ^{13}C chemical shifts were externally referenced to the α-glycine C(O)O$^-$ signal at 176.04 ppm on the TMS scale and ^{15}N shifts were referenced to the N$^{\alpha}$ signal of histidine·HCl at 49.04 ppm on the liquid NH_3 scale. ^{1}H shifts were calibrated by the $^{1}H–^{13}C$ FSLG-decoupled dipolar correlation experiment on tyrosine·HCl through its Hβ and Hη resonances at 2.1 and 13.1 ppm, respectively. Intra- and intermolecular ^{1}H contacts of the PCB chromophore (Supplementary Tables S5–S9) were assigned based on the corresponding structural models for Pr and Pfr (deposited in Supplementary Material) and validated in the same manner as in Song et al. (2011, 2012). Data were preprocessed with Topspin 3.1 (Bruker BioSpin, Rheinstetten) and subsequently analyzed with the aid of Sparky 3.115 (T. D. Goddard and D. G. Kneller, University of California, San Francisco, CA). All Pfr spectra were obtained by subtraction of the corresponding Pr spectra (Song et al., 2012) from those of the Pfr/Pr (4:1) mixture used in this study with an appropriate weighting constant of ~0.2. More specifically, the raw data (2D fid) of the Pr dataset were subtracted from those of the Pfr/Pr

mixture using the standard '*Add / subtract*' module embedded in the Topspin program. The 2D Pr and Pfr/Pr datasets have equal sizes. The multiplication factor (ALPHA) of the 2D spectrum of Pfr/Pr mixture was set to be 1, and the multiplication factor (GAMMA) of the Pr spectrum was tuned down from −0.15 to −0.25. The optimal GAMMA value (Pr weighting constant) was found to be at −0.20 with the complete disappearance of well-resolved Pr-only correlations. For example, the DARR correlations of C18–C19 and $C8^1$–$C8^3$ were taken as the reference for obtaining the corresponding Pfr DARR spectrum, both were fully resolved and clearly isolated from other correlations.

Improved structural model as Pr. In our previous paper (Song et al., 2012) the Pr signals were assigned with the help of a QM/MM-optimized homology model for the oat phyA3 sensory module based on that of *Arabidopsis* phyA with the native PΦB chromophore (Mroginski et al., 2011). Here we present an improved model for Pr based on reassignments of the two interfacial ^{1}H–^{13}C heteronuclear correlation spectra using the PCB model corresponding to the *u*-[^{13}C,^{15}N]-PCB adduct investigated experimentally (Supplementary Figures S4, S5).

Rotamer definitions for tyrosine. The side chain torsional angles χ_1 and χ_2 represent the dihedral angles of Nα–Cα–Cβ–Cγ and Cα–Cβ–Cγ–Cδ1, respectively. χ_1, conformation g^+: 0°–120°; conformation *t*: 120°–240°; conformation g^-: 240°–0°, and χ_2, conformation g^+: 30°–90° and 210°–270°; conformation g^-: 90°–150° and 270°–330°; conformation *t*: 150°–210° and 330°–30° (Shapovalov and Dunbrack, 2011).

The figures were rendered using MacPyMOL (The PyMOL Molecular Graphics System, Version 1.3 Schrödinger, LLC) and Adobe Illustrator CS5 (Adobe) software.

SUPPLEMENTARY REFERENCES

Bakowies, D., and Thiel, W. (1996). Hybrid models for combined quantum mechanical and molecular mechanical approaches. *J. Phys. Chem.* 100, 10580–10594. doi: 10.1021/jp9536514

Billeter, S., Turner, A. J., and Thiel, W. (2000). Linear scaling geometry optimization and transition state search in hybrid delocalised internal coordinates. *Phys. Chem. Chem. Phys.* 2, 2177–2186. doi: 10.1039/a909486e

Brooks, B. R., Brooks, C. L., III, Mackerell, A. D., Jr., Nilsson, L., Petrella, R. J., Roux, B., et al. (2009). CHARMM: The biomolecular simulation program. *J. Comput. Chem.* 30, 1545–1614. doi: 10.1002/jcc.21287

Bruce, S. D., Higinbotham, J., Marshall, I., and Beswick, P. H. (2000). An analytical derivation of a popular approximation of the Voigt function for quantification of NMR spectra. *J. Magn. Reson.* 142, 57–63. doi: 10.1006/jmre.1999.1911

Burgie, E. S., Bussell, A. N., Walker, J. M., Dubiel, K., and Vierstra, R. D. (2014). Crystal structure of the photosensing module from a red/far-red light-absorbing plant phytochrome. *Proc. Natl. Acad. Sci. U.S.A.* 111, 10179–10184. doi: 10.1073/pnas.1403096111

Darden, T., York, D., and Pedersen, L. (1993). Particle mesh Ewald: An $N \cdot \log(N)$ method for Ewald sums in large systems. *J. Chem. Phys.* 98, 10089–10092. doi: 10.1063/1.464397

Ditchfield, R. (1976). GIAO studies of magnetic shielding in FHF^{-} and HF. *Chem. Phys. Lett.* 40, 53–56. doi: 10.1016/0009-2614(76)80119-4

Essen, L.-O., Mailliet, J., and Hughes, J. (2008). The structure of a complete phytochrome sensory module in the Pr ground state. *Proc. Natl. Acad. Sci. U.S.A.* 105, 14709–14714. doi: 10.1073/pnas.0806477105

Feller, S. E., Zhang, Y., Pastor, R. W., and Brooks, B. R. (1995). Constant pressure molecular dynamics simulation: The Langevin piston method. *J. Chem. Phys.* 103, 4613–4621, doi: 10.1063/1.470648

Frisch, M. J., Trucks, G. W., Schlegel, H. B., Scuseria, G. E., Robb, M. A., Cheeseman, J. R., et al. (2016). Gaussian 09, Revision A.02. Gaussian, Inc., Wallingford CT.

Heringa, J. (1999). Two strategies for sequence comparison: profile-preprocessed and secondary structure-induced multiple alignment. *Comput. Chem.* 23, 341–364. doi: 10.1016/S0097-8485(99)00012-1.

Jorgensen, W. L., Chandrasekhar, J., Madura, J. D., Impey, R. W., and Klein, M. L. (1983). Comparison of simple potential functions for simulating liquid water. *J. Chem. Phys.* 79, 926–935. doi: 10.1063/1.445869

Kaminski, S., and Mroginski, M. A. (2010). Molecular dynamics of phycocyanobilin binding bacteriophytochromes: A detailed study of structural and dynamic properties. *J. Phys. Chem. B* 114, 16677–16686. doi: 10.1021/jp104903u

Kiihne, S. R., Creemers, A. F. L., de Grip, W. J., Bovee-Geurts, P. H. M., Lugtenburg, J., and de Groot, H. J. M. (2005). Selective interface detection: Mapping binging site contacts in membrane proteins by NMR spectroscopy. *J. Am. Chem. Soc.* 127, 5734–5735. doi: 10.1021/ja045677r

Li, S., and Hong, M. (2011). Protonation, tautomerization, and rotameric structure of histidine: A comprehensive study by magic-angle-spinning solid-state NMR. *J. Am, Chem. Soc.* 133, 1534–1544. doi: 10.1021/ja108943n

MacKerell, A. D., Jr., Banavali, N., and Foloppe, N. (2001). Development and current status of the CHARMM force field for nucleic acids. *Biopolymers* 56, 257–265. doi: 10.1002/10970282(2000)56:4<257::aid-bip10029>3.0.co;2-w.

Metz, S., Kästner, J., Sokol, A. A., Keal, T. W., and Sherwood, P. (2014). ChemShell—a modular software package for QM/MM simulations. *WIREs Mol. Sci.* 4, 101–110. doi: 10.1002/wcms.1163

Mroginski, M. A., von Stetten, D., Velazquez Escobar, F., Strauss, H. M., Kaminski, S., Scheerer, P., et al. (2009). Chromophore structure of cyanobacterial phytochrome Cph1 in the Pr state: Reconciling structural and spectroscopic data by QM/MM calculations. *Biophys. J.* 96, 4153–4163. doi: 10.1016/j.bpj.2009.02.029

Mroginski, M. A., Kaminski, S., von Stetten, D., Ringsdorf, S., Gärtner, W., Essen, L.-O., et al. (2011). Structure of the chromophore binding pocket in the Pr state of plant phytochrome phyA. *J. Phys. Chem. B* 115, 1220–1231. doi: 10.1021/jp108265h

Pettersen, E. F., Goddard, T. D., Huang, C. C., Couch, G. S., Greenblatt, D. M., Meng, E. C., et al. (2004). UCSF Chimera—A visualization system for exploratory research and analysis. *J. Comput. Chem.* 25, 1605–1612. doi: 10.1016/j.bpj.2009.02.029

Phillips, J. C., Braun, R., Wang, W., Gumbart, J., Tajkhorshid, E., Villa, E., et al. (2005). Scalable molecular dynamics with NAMD. *J. Comput. Chem.* 26, 1781–1802. doi: 10.1002/jcc.20289

Rockwell, N. C., Martin, S. S., Lim, S., Lagarias, J. C., and Ames, J. B. (2015). Characterization of red/green cyanobacteriochrome NpR6012g4 by solution nuclear magnetic resonance spectroscopy: A protonated bilin ring system in both photostates. *Biochemistry* 54, 2581−2600. doi: 10.1021/bi501548t

Rohmer, T., Lang, C., Hughes, J., Essen, L.-O., Gartner, W., and Matysik, J. (2008). Light-induced chromophore activity and signal transduction in phytochromes observed by ^{13}C and ^{15}N magic-angle spinning NMR. *Proc. Natl. Acad. Sci. U.S.A.* 105, 15229–15234. doi: 10.1073/pnas.0805696105

Rohmer, T., Lang, C., Bongards, C., Gupta, K. B. S. S., Neugebauer, J., Hughes, J., et al. (2010). Phytochrome as molecular machine: Revealing chromophore action during the Pfr → Pr photoconversion by magic-angle spinning NMR spectroscopy. *J. Am. Chem. Soc.* 132, 4431–4437. doi: 10.1021/ja9108616

Ryckaert, J.-P., Ciccotti, G., and Berendsen, H. J. C. (1977). Numerical integration of the Cartesian equations of motion of a system with constraints: Molecular dynamics of *n*-alkanes. *J. Comput. Phys.* 23, 321–341. doi: 10.1016/0021-9991(77)90098-5

Šali, A., and Blundell, T. L. (1993). Comparative protein modelling by satisfaction of spatial restraints. *J. Mol. Biol.* 234, 779–815. doi: 10.1006/jmbi.1993.1626

Siemer, A. B., Huang, K.-Y., and McDermott, A. E. (2012). Protein linewidth and solvent dynamics in frozen solution NMR. *PLoS ONE* 7, e47242. doi: 10.1371/journal.pone.0047242

Shapovalov, M. V., and Dunbrack, R. L. Jr. (2011). A smoothed backbone-dependent rotamer library for proteins derived from adaptive kernel density estimates and regressions. *Structure* 19, 844−858. doi: 10.1016/j.str.2011.03.019

Song, C., Psakis, G., Lang, C., Mailliet, J., Gärtner, W., Hughes, J., et al. (2011). Two ground state isoforms and a chromophore ***D***-ring photoflip triggering extensive intramolecular changes in a canonical phytochrome. *Proc. Natl. Acad. Sci. U.S.A.* 108, 3842–3847. doi: 10.1073/pnas.1013377108

Song, C., Essen, L.-O., Gärtner, W., Hughes, J., and Matysik, J. (2012). Solid-state NMR spectroscopic study of chromophore–protein interactions in the Pr ground state of plant phytochrome A. *Mol. Plant* 5, 689–715. doi: 10.1093/mp/sss017

Song, C., Rohmer, T., Tiersch, M., Zaanen, J., Hughes, J., and Matysik, J. (2013). Solid-state NMR spectroscopy to probe photoactivation in canonical phytochromes. *Photochem. Photobiol.* 89, 259–273. doi: 10.1111/php.12029

Stöppler, D., Song, C., van Rossum, B.-J., Geiger, M.-A., Lang, C., Mroginski, M.-A., et al. (2016). Dynamic nuclear polarization provides new insights into chromophore structure in phytochrome photoreceptors. *Angew. Chem. Int. Ed.* 55, 16017–16020. doi: 10.1002/anie.201608119

van Rossum, B. J., Schulten, E. A. M., Raap, J., Oschkinat, H., and de Groot, H. J. M. (2002). A 3-D structural model of solid self-assembled chlorophyll *a*/H2O from multispin labelling and MAS NMR 2-D dipolar correlation spectroscopy in high magnetic field. *J. Magn. Reson.* 155, 1–14. doi: 10.1006/jmre.2002.2502

Velazquez Escobar, F., Lang, C., Takiden, A., Schneider, C., Balke, J., Hughes, J., et al. (2017). Protonation-dependent structural heterogeneity in the chromophore binding site of cyanobacterial phytochrome Cph1. *J. Phys. Chem. B* 121, 47–57. doi: 10.1021/acs.jpcb.6b09600

Yang, X., Kuk, J., and Moffat, K. (2008). Crystal structure of *Pseudomonas aeruginosa* bacteriophytochrome: Photoconversion and signal transduction. *Proc. Natl. Acad. Sci. U.S.A.* 105, 14715–14720. doi: 10.1073/pnas.0806718105

Yao, L., Schmidt-Rohr, K., and Hong, M. (2001). Medium- and long distance ^{1}H–^{13}C heteronuclear correlation NMR in solids. *J. Magn. Reson.* 149, 139–143. doi: 10.1006/jmre.2001.2285

SUPPLEMENTARY FIGURES

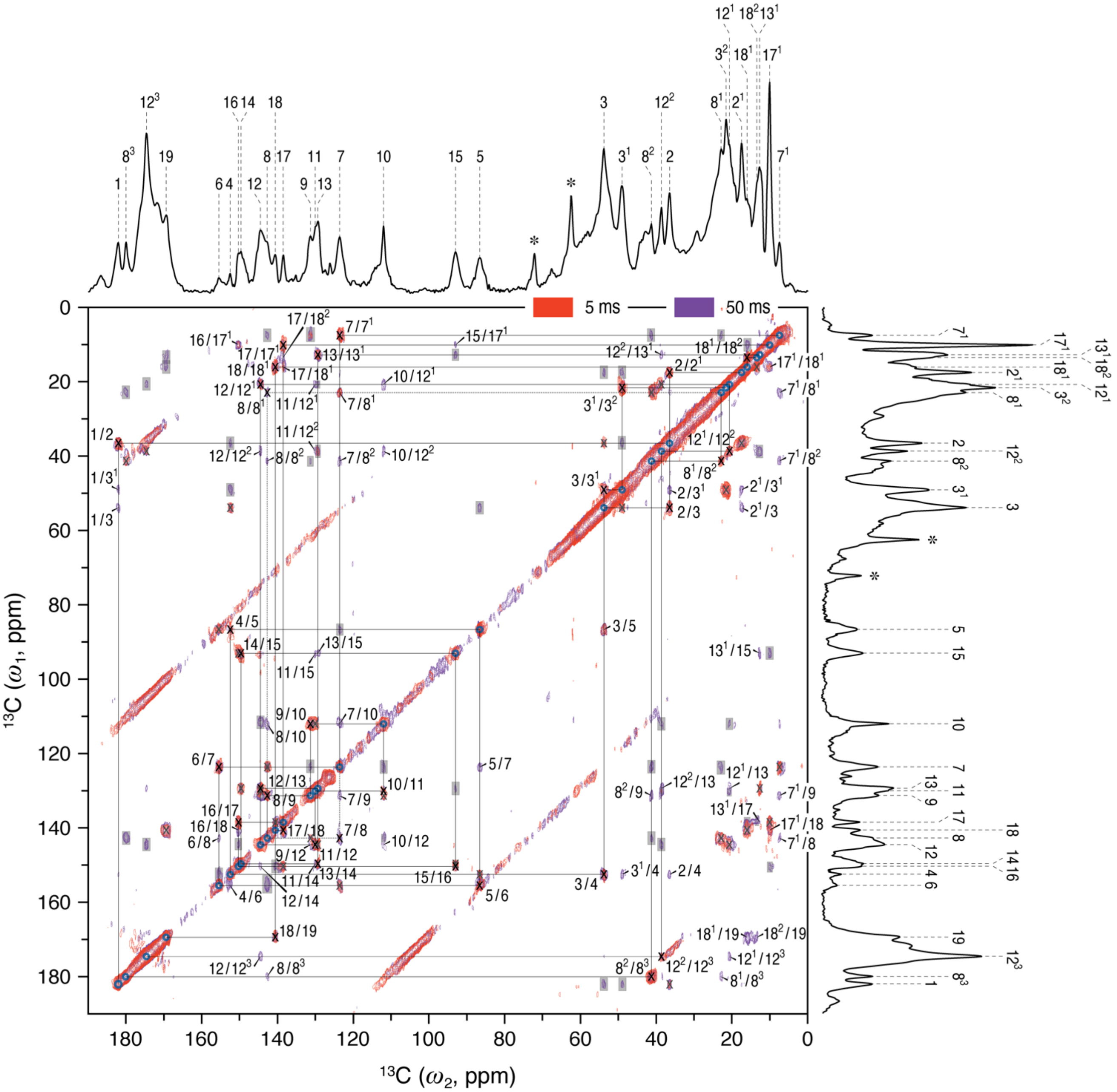

Supplementary Figure 1. Enlarged contour plots of the DARR spectra shown in Figure 1 with complete chromophore assignments. Two DARR spectra of *u*-[^{13}C,^{15}N]-PCB oat phyA3 as Pfr were acquired with the mixing times of 5 (red) and 50 (purple) ms. The 1D traces of the 2D spectra (along ω_1-, right, and ω_2-dimension, top) are shown with the assignment (see Figure 3 for PCB chromophore numbering). Asterisks indicate peaks arising from the natural abundance glycerol carbons. The indirectly-bonded ^{13}C–^{13}C correlations are indicated by the arrows and labeled in black and their corresponding off-diagonal counterparts shaded in gray. The one-bond correlations and their diagonal counterparts are marked × in black and gray, respectively.

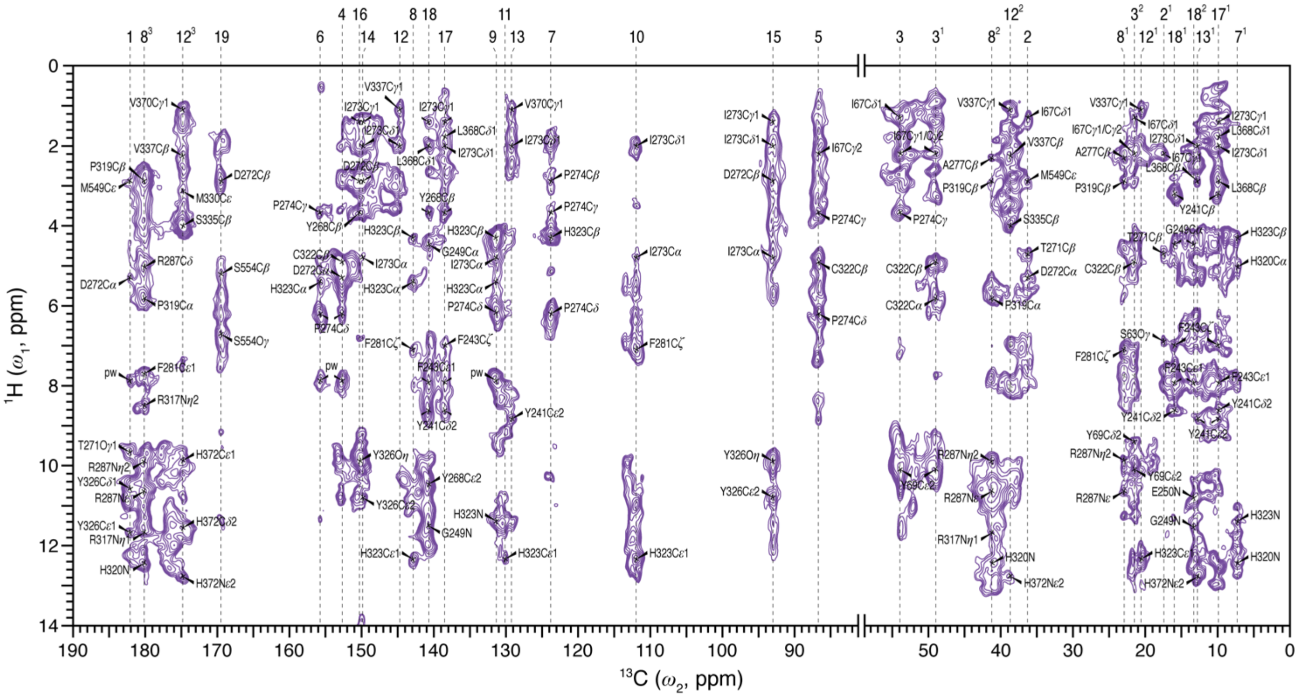

Supplementary Figure S2. Interfacial ^{1}H−^{13}C contacts between the PCB chromophore carbons and the surrounding residues (within the region of ~4.5 Å) obtained from *u*-[^{13}C,^{15}N]-PCB oat phyA3 as Pfr. The 2D MELODI−HETCOR spectrum was acquired with an LG−CP contact time of 2.3 ms. The resolved ^{1}H contacts of chromophore are solely due to its interactions with protons bound to the direct protein surrounding (summarized in Supplementary Tables S5, S6). Corresponding spectra of this protein at natural abundance and the intramolecular H^N contacts of the chromophore have been subtracted. The spectral region without resonances, 60–80 ppm in the ω_2-dimension is omitted and ^{13}C resonances of the chromophore are labeled above the vertical lines according to the 2D DARR spectra shown in Figure 1. The data were collected and processed as follows: 3072 scans with a recycle delay of 1.5 s for each t_1 point, and a total of 128 points combined in the ω_1-dimension. The t_1 data were recorded in an off-resonance manner and linear predicted by 32 points using 40 LP coefficient. A 90° shifted squared sine bell window function was applied, and zero-filled to 2048 points prior to Fourier transformation. The notation for ^{1}H assignment uses oat phyA3 numbering of residues (Supplementary Table S13), followed by the Greek letter referring to the proton identity.

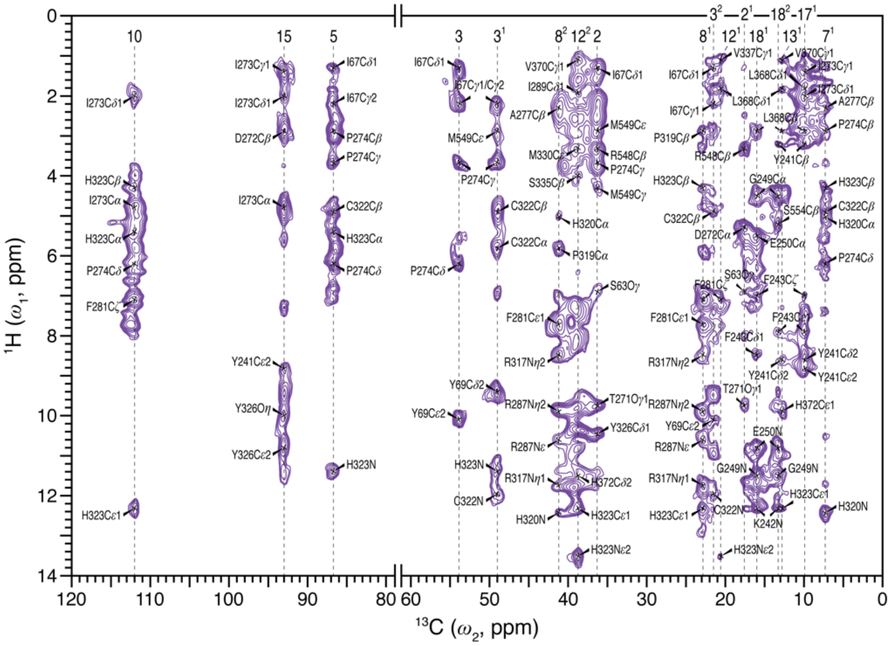

Supplementary Figure S3. Selective observation of interfacial 2.5–6.5 Å ^{1}H contacts of the chromophore non-quaternary carbons obtained from *u*-[^{13}C,^{15}N]-PCB oat phyA3 as Pfr. The SIDY data were collected with a ^{1}H spin diffusion mixing time of 800 μs and a CP contact time of 96 μs and with 1536 complex t_2 and 128 real t_1 points with 2664 scans. A recycle delay of 1.5 s was used. The resolved ^{1}H–^{13}C correlations arise solely from the interactions between non-quaternary chromophore carbons and the protons bound to amino acids nearby (summarized in Supplementary Tables S7, S8). Background signals were suppressed by subtracting the SIDY Pfr spectrum of this protein at natural abundance. The spectral region without resonances, 60–80 ppm in the ω_2-dimension is omitted. ^{13}C resonances of the chromophore are labeled above the vertical lines. The notation for ^{1}H assignment uses oat phyA3 numbering of residues (Supplementary Table S13), followed by the Greek letter referring to the proton identity.

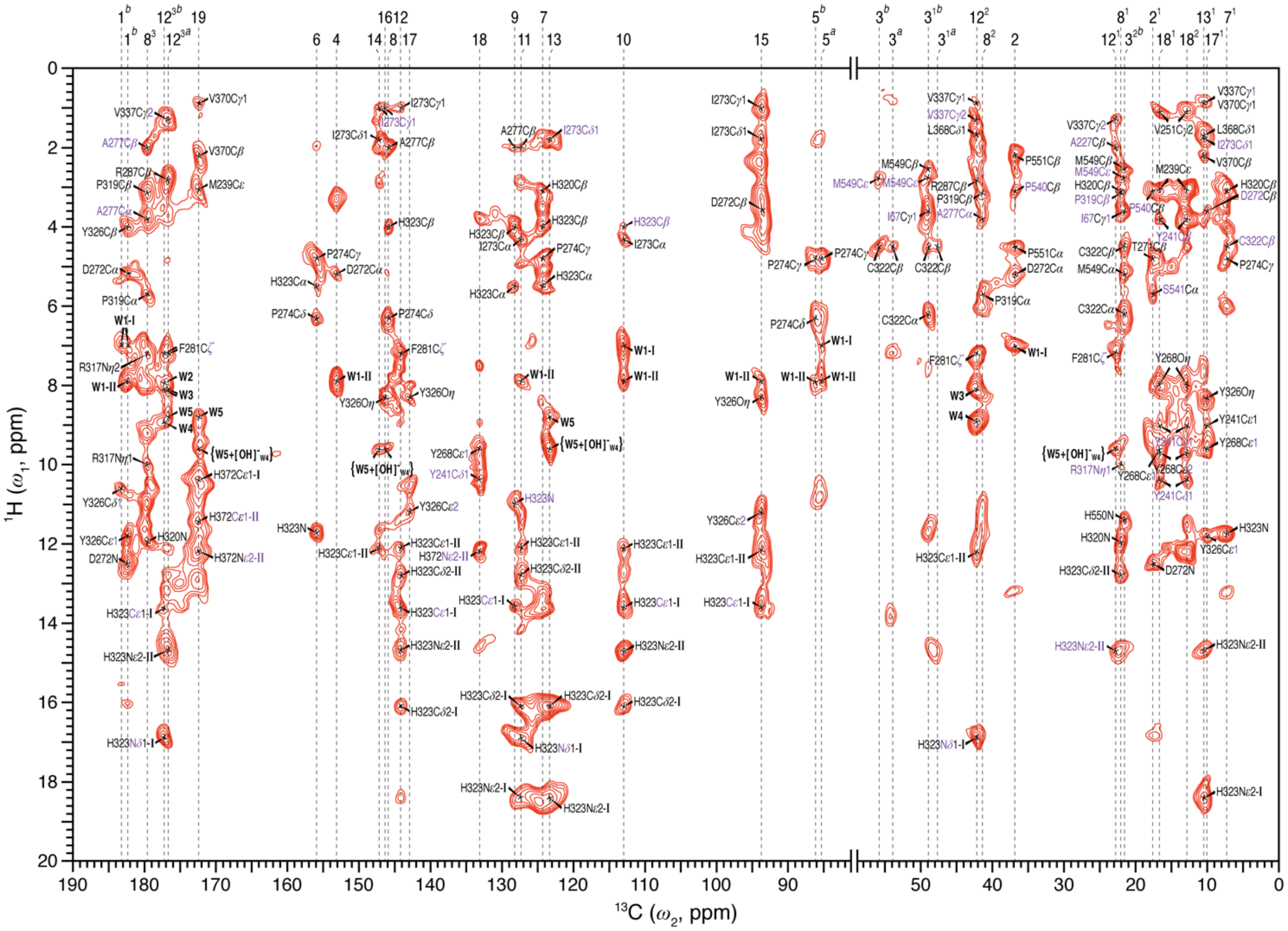

Supplementary Figure S4. Resonance reassignment of MELODI–HETCOR spectrum of oat phyA3 as Pr. Our previous tentative ^{1}H assignment for the heteronuclear correlation spectrum of the *u*-[^{13}C,^{15}N]-PCB adduct of oat phyA3 as Pr was based on a molecular model for the PΦB adduct of *Arabidopsis* phyA (Song et al., 2012). Here the optimized homology model of oat phyA3 PCB adducts was used to reassign the ^{1}H resonances. Model-derived changes on the initial assignment (annotations in black) are colored purple and tracked in Supplementary Table S10.

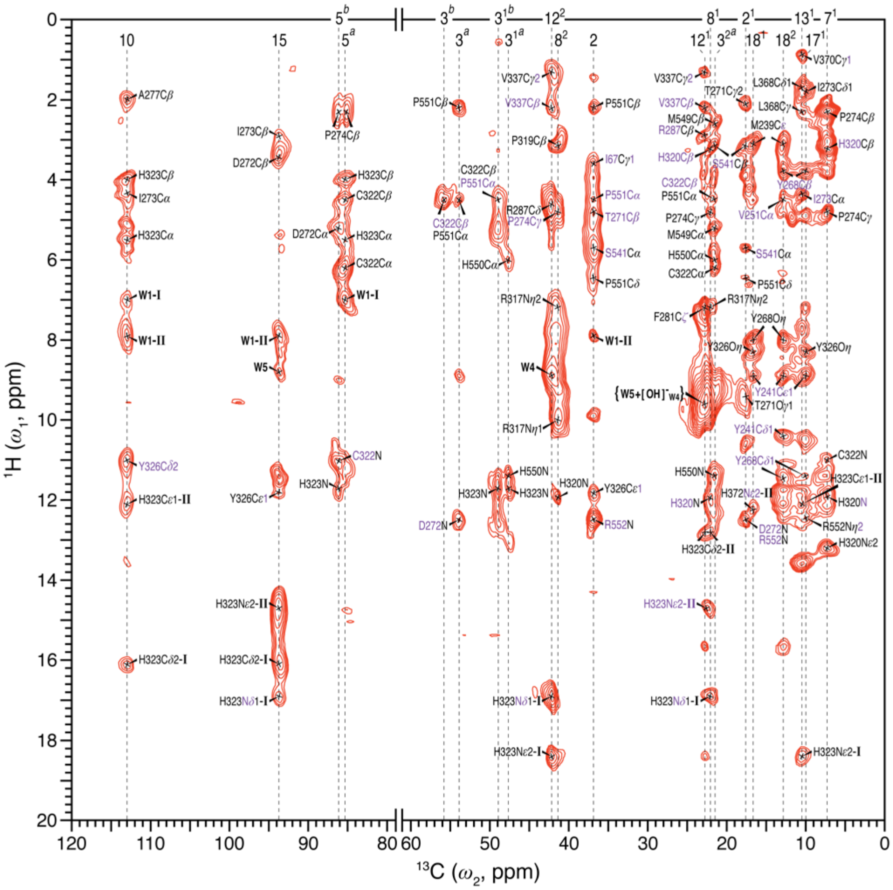

Supplementary Figure S5. Resonance reassignments of SIDY spectrum of oat phyA3 as Pr. Our previous tentative ^{1}H assignment for the SIDY correlation spectrum of the *u*-[^{13}C,^{15}N]-PCB adduct of oat phyA3 as Pr was based on a molecular model for the PΦB adduct of *Arabidopsis* phyA (Song et al., 2012). Here the optimized homology model of oat phyA3 PCB adducts was used to reassign the ^{1}H resonances. Model-derived changes on the initial assignment (annotations in black) are colored purple and tracked in Supplementary Table S11.

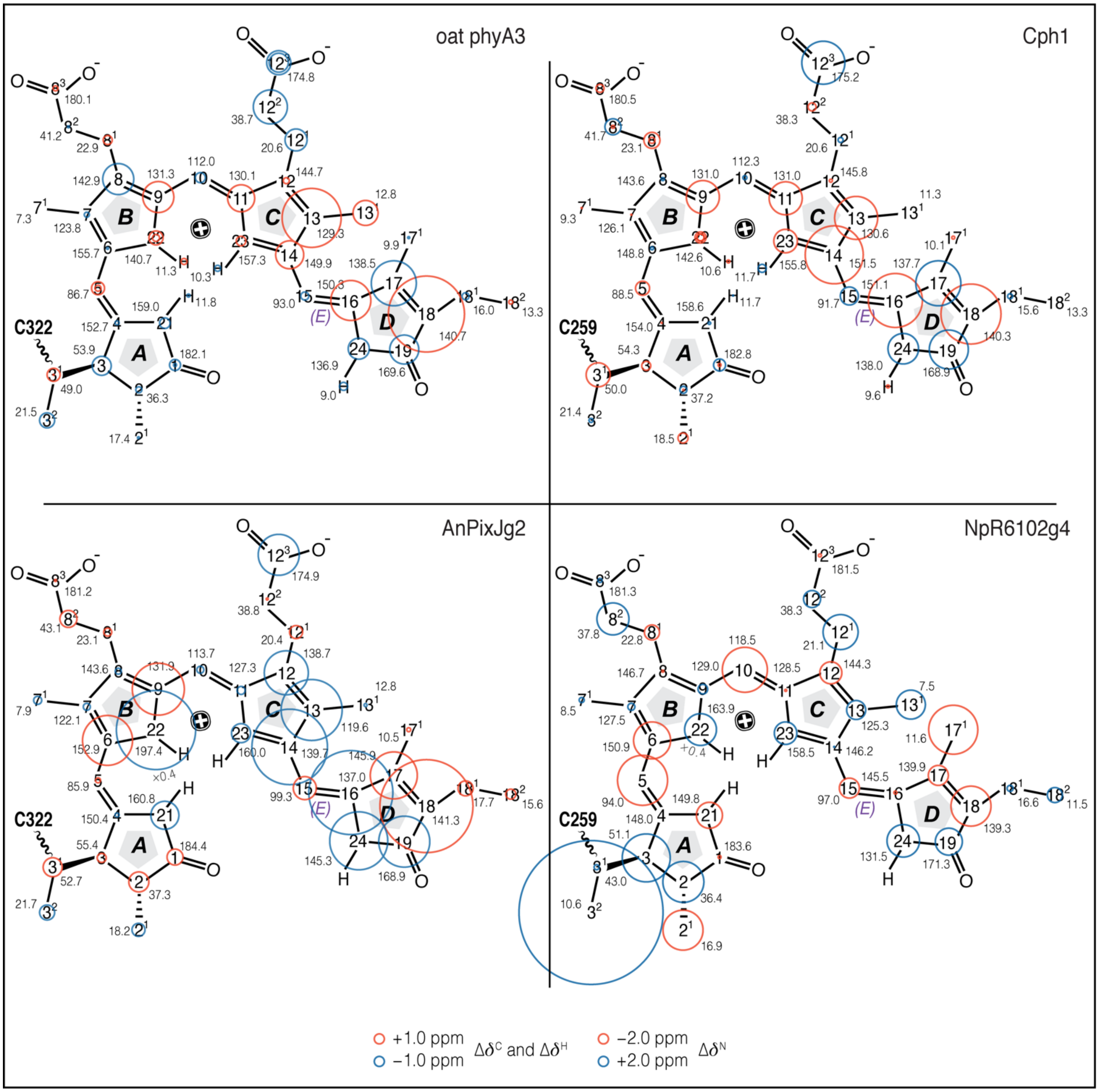

Supplementary Figure S6. State-related changes in ^{13}C and ^{15}N chemicals shifts of the PCB chromophore in phytochromes oat phyA3 and Cph1 and in two red/green CBCRs AnPixJg2 and NpR6102g4. Light-induced changes of ^{13}C and ^{15}N chemical shifts of the chromophore ($\Delta\delta^{C}$ and $\Delta\delta^{N}$) are represented as red and blue circles. In all cases, the 15*Z* dark state is taken as reference, and the size of the circles is proportional to the 15*E* photoproduct minus 15*Z* dark state difference. Carbon and nitrogen atoms showing signal doublings are labeled with two circles. δ^{C} and δ^{N} changes accompanying 15*Z*-to-15*E* isomerization in oat phyA3 are summarized in Supplementary Table S1. Data from the other three photosensors, Cph1, AnPixJg2, and NpR6102g4 are taken from Song et al., 2013, Song et al., 2015, and Rockwell et al., 2015, respectively.

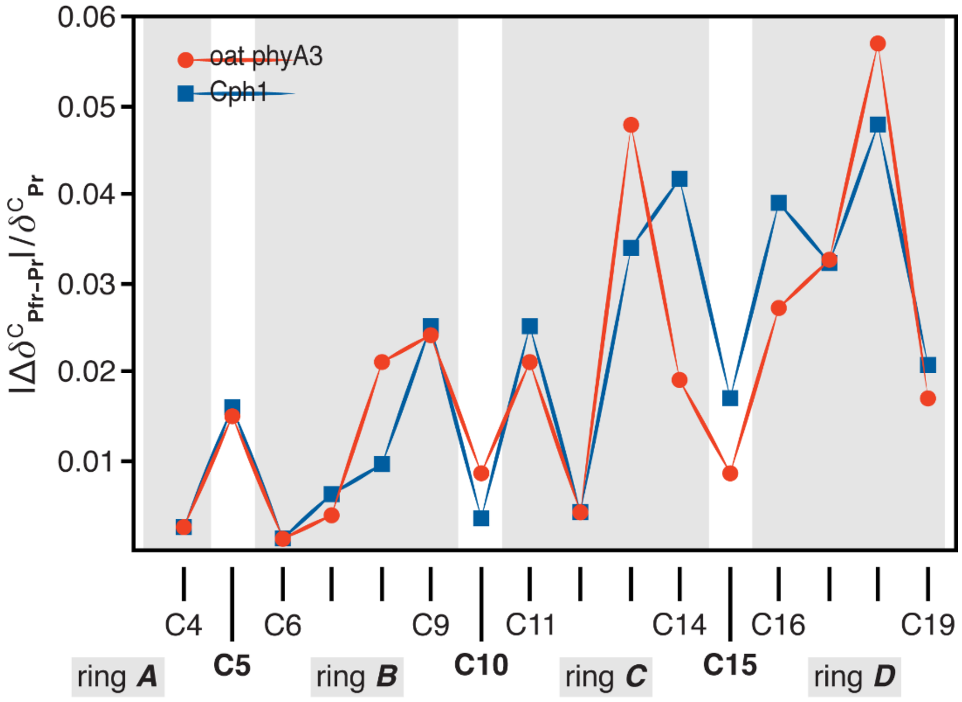

Supplementary Figure S7. State-related changes in π-conjugated ^{13}C chemical shifts for oat phyA3 and Cph1. The Pr→Pfr changes were calculated as $|\Delta\delta^{C}_{\mathbf{Pfr-Pr}}| / \delta^{C}_{\mathbf{Pr}}$ (see Rockwell et al., 2015) for each carbon atom in the π-chain.

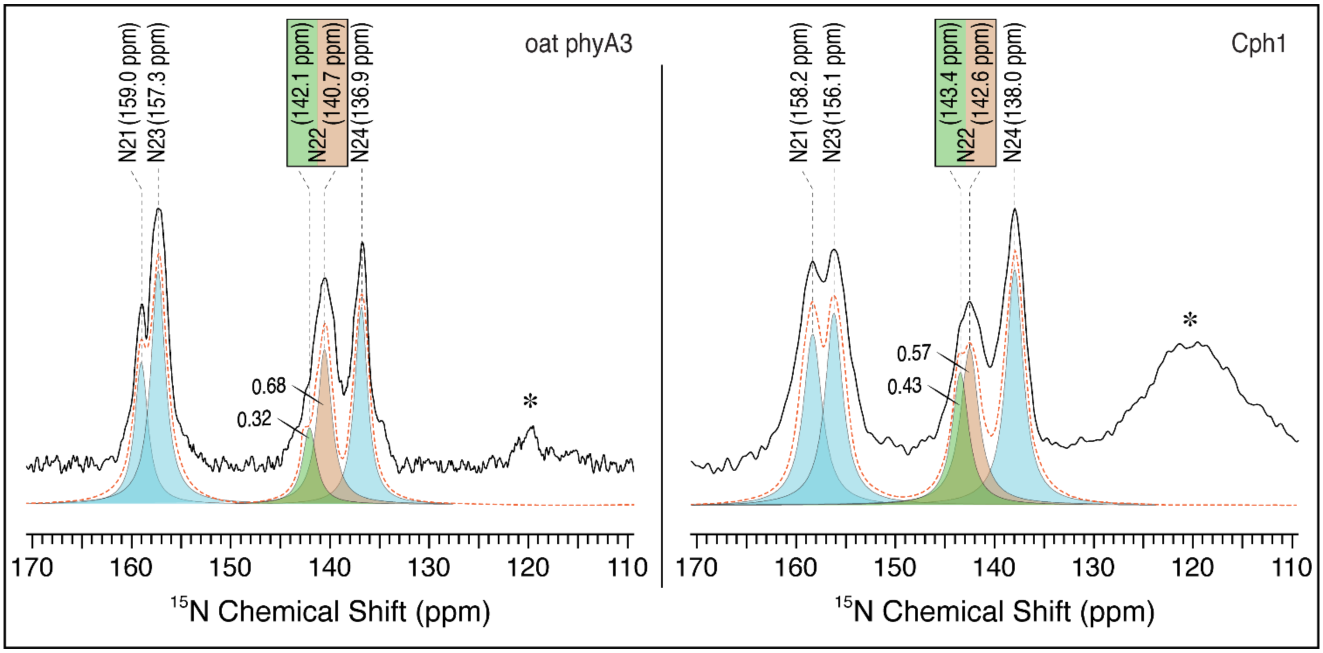

Supplementary Figure S8. ^{15}N Pfr signal splitting associated with the ***B***-ring nitrogen in oat phyA3 and Cph1. A Voigt function with a mixed Lorentzian–Gaussian ratio of 1:1 was applied to fit the experimental ^{15}N Pfr spectra of oat phyA3 (Figure 2B) and Cph1 (Rohmer et al., 2008). Fitting curves (dashed) are shown in red. Voigt deconvolution of both molecules revealed three single-component maxima (N21, N23, and N24, shaded cyan) and a peak maximum at ~141 ppm (N22, shaded green and orange, the relative areas are given). The two Pfr spectra are closely similar at the positions of all four maxima (≤1.5 ppm) and the signal splitting pattern associated with the ***B***-ring nitrogen.

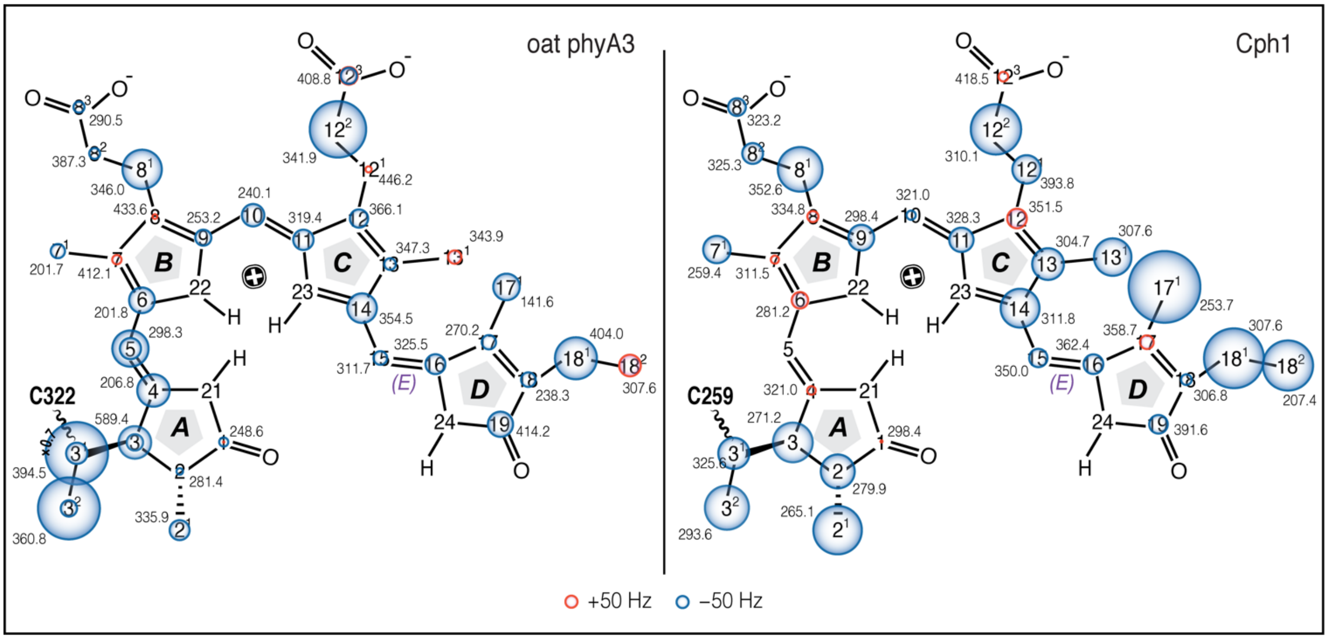

Supplementary Figure S9. State-related changes in ^{13}C resonance FWHM line-widths ($\nu_{1/2}$) of the chromophore in oat phyA3 and Cph1. The $\nu_{1/2}$ values of the Pfr resonances are given (black numbers) and the Pr → Pfr $\Delta\nu_{1/2}$ of the PCB carbon resonances are represented as red and blue circles for broader and narrower resonances in Pfr, respectively. The size of the shaded circles is proportional to the $\Delta\nu_{1/2}$ as Pfr minus Pr. Carbon atoms showing Pr signal doublings are labeled with two circles. $\Delta\nu_{1/2}$ for oat phyA3 and Cph1 (summarized in Supplementary Table S4) are from this study and Song et al., 2013, respectively.

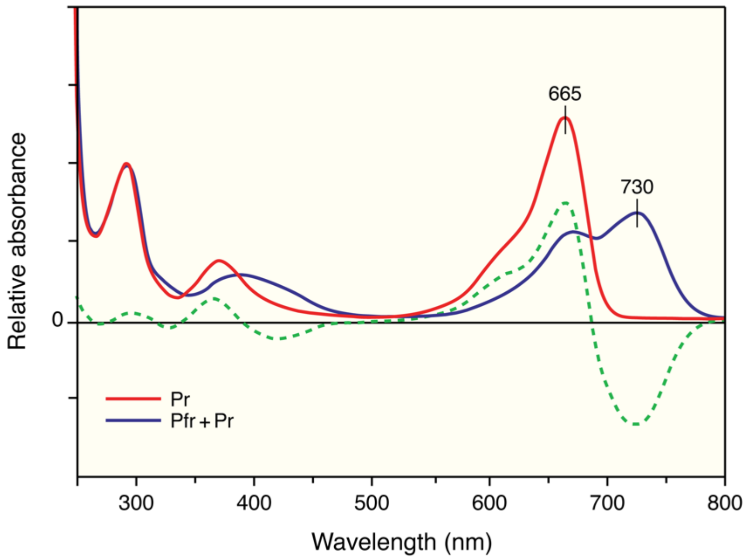

Supplementary Figure S10. UV-visible absorbance spectra of the oat phyA3 preparations used in this study. Pr (red), Pr/Pfr photoequilibrium mixture (blue) and calculated difference (green dashes) spectra were recorded following 60-sec irradiation with 725 ± 8 and 660 ± 8 nm light, respectively.

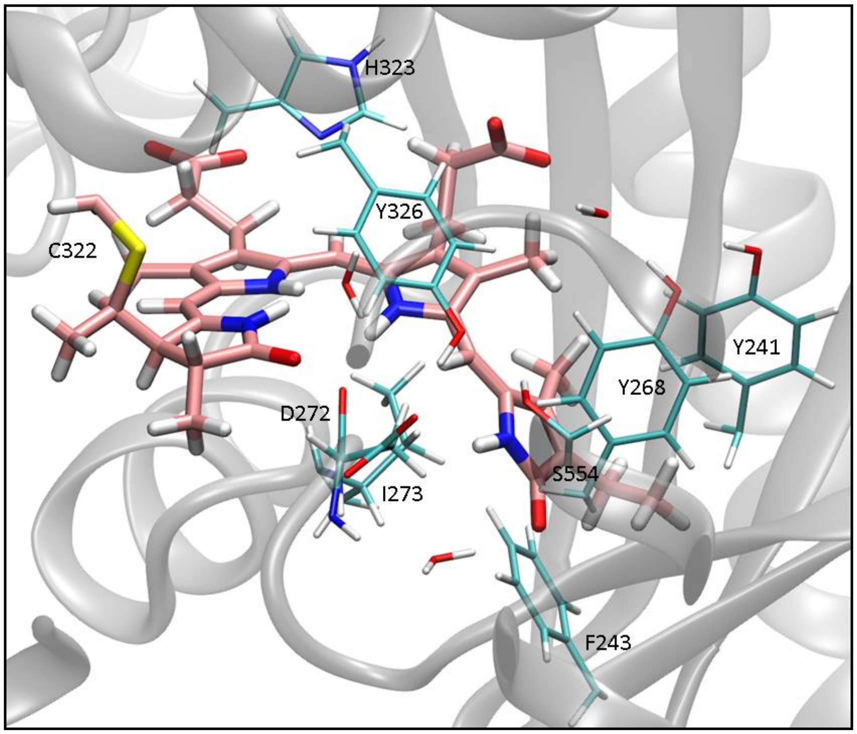

Supplementary Figure S11. Definition of the 198-atom QM region for QM/MM calculations of oat phyA3. The PCB chromophore (pink), D272 and I273, the side chains of C322, Y241, F243, Y268, H323, Y326 and S554 as well as three water molecules participating in hydrogen-bonding interactions with the chromophore are shown.

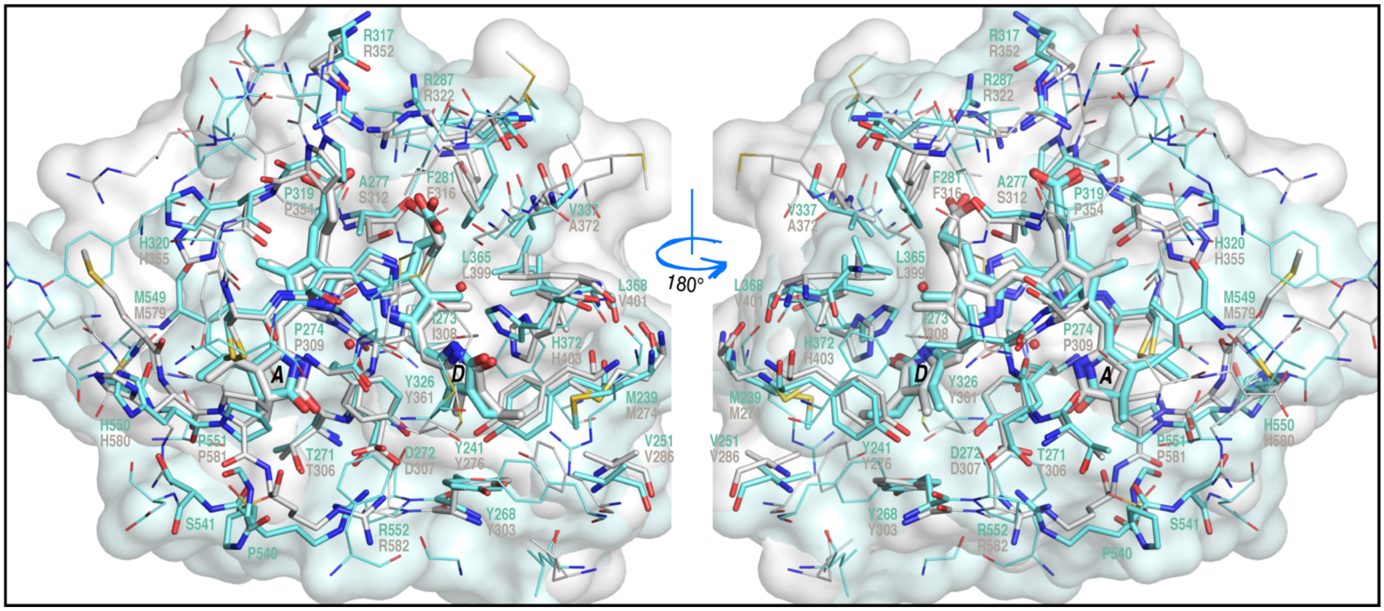

Supplementary Figure S12. Superimposition of the Pr chromophore pocket of oat phyA3 (cyan; PDB file deposited in Supplementary Material) and *Arabidopsis* phyB (gray; Burgie et al., 2014). The residues observed with MELODI–HETCOR and SIDY experiments on oat phyA3 (summarized in Supplementary Table S9) and the homologous residues in *Arabidopsis* phyB (see Supplementary Table S13) are shown in stick mode.

Supplementary Table S1. Overview of ^{13}C and ^{15}N chemical shifts of the *u*-[^{13}C,^{15}N]-PCB chromophore in *As*.phyA3 as Pr and Pfr. Published ^{13}C and ^{15}N data of *As*.phyA3 and Cph1 are listed for reference (Rohmer et al., 2008, 2010; Song et al., 2012). The ^{13}C and ^{15}N chemical shift differences ($\Delta\delta^{C}_{Pfr-Pr}$ and $\Delta\delta^{N}_{Pfr-Pr}$) during Pr-to-Pfr photoconversion (listed at the right-most column) were reported as Pfr (present work) minus Pr (Song et al., 2012) and illustrated in Figure 3A. The chromophore numbering is according to Figure 3A.

		Oat PhyA3–PCB		
	cofactor carbon	δ^{C}_{Pr} (ppm)	δ^{C}_{Pfr} (ppm)	$\Delta\delta^{C}_{Pfr-Pr}$ (ppm)
ring A	**1**	182.4[†](182.6[‡]) (1^{a})	182.1[§](182.5[‡])	**−0.3**
		183.2(n.d.) (1^{b})		**−1.1**
	**2**	36.9(36.8)	36.3(37.0)	**−0.6**
	**2^{1}**	17.6(17.7)	17.4(17.7)	**−0.2**
	**3**	53.9(53.9) (3^{a})	53.9(53.9)	**0.0**
		55.8(n.d.) (3^{b})		**−1.9**
	**3^{1}**	47.7(45.8) (3^{1a})	49.0(49.1)	**+1.3**
		48.9(47.6) (3^{1b})		**+0.1**
	**3^{2}**	23.0(23.0) (3^{2a})	21.5(21.3)	**−1.5**
		21.6(21.7) (3^{2b})		**−0.1**
	**4**	153.1(n.d.)	152.7(n.d.)	**−0.4**
A–B	**5**	85.4(n.d.) (5^{a})	86.7(87.1)	**+1.3**
		86.1(85.8) (5^{b})		**+0.6**
ring B	**6**	155.9(n.d.)	155.7(n.d.)	**−0.2**
	**7**	124.3(124.7)	123.8(124.6)	**−0.5**
	**7^{1}**	7.3(7.2)	7.3(7.5)	**0.0**
	**8**	146.0(146.4)	142.9(143.4)	**−3.1**
	**8^{1}**	22.0(21.8)	22.9(23.0)	**+0.9**
	**8^{2}**	41.4(41.5)	41.2(41.1)	**−0.2**
	**8^{3}**	179.6(179.9)	180.1(179.7)	**+0.5**
	**9**	128.2(127.7)	131.3(130.6)	**+3.1**
B–C	**10**	113.0(113.0)	112.0(111.9)	**−1.0**
ring C	**11**	127.4(127.7)	130.1(130.6)	**+2.7**
	**12**	144.1(146.4)	144.7(144.5)	**+0.6**
	**12^{1}**	22.8(21.8)	20.6(21.0)	**−2.2**
	**12^{2}**	42.1(41.5)	38.7(39.0)	**−3.4**
	**12^{3}**	176.7(n.d.) (12^{3a})	174.8(174.7)	**−1.9**
		177.2(177.5) (12^{3b})		**−2.4**
	**13**	123.4(123.7)	129.3(130.1)	**+5.9**
	**13^{1}**	10.3(10.0)	12.8(12.1)	**+2.5**
	**14**	147.1(146.6)	149.9(150.5)	**+2.8**
C–D	**15**	93.8(93.3)	93.0(92.3)	**−0.8**
ring D	**16**	146.3(146.6)	150.3(150.5)	**+4.0**
	**17**	143.0(142.6)	138.5(138.6)	**−4.5**
	**17^{1}**	10.1(10.3)	9.9(10.3)	**−0.2**
	**18**	133.1(133.3)	140.7(140.1)	**+7.6**
	**18^{1}**	16.7(16.6)	16.0(n.d.)	**−0.7**
	**18^{2}**	12.9(n.d.)	13.3(n.d.)	**+0.4**
	**19**	172.4(172.4)	169.5(169.6)	**−2.9**
	pyrrole nitrogen	δ^{N}_{Pr} (ppm)	δ^{N}_{Pfr} (ppm)	$\Delta\delta^{N}_{Pfr-Pr}$ (ppm)
ring A	**21**	156.9[†](158.1[‖])	159.0[§](158.2[‖])	**+2.1**
ring B	**22**	143.5[†](144.8)	140.7(142.6) (22^{a})	**−2.8**
			142.1(143.4) (22^{b})	**−1.4**
ring C	**23**	159.2(160.5)	157.3(156.1)	**−1.9**
ring D	**24**	132.7(131.9)	136.9(138.0)	**+4.2**

[†]Song et al., 2012 on oat phyA3 Pr; [‡]Rohmer et al., 2008 on oat phyA3 Pr and Pfr; [‖]Rohmer et al., 2010 on Cph1 Pr and Pfr; [§]present work;
n.d.: not determined; ^{15}N assignments of N22 and N23 as Pr were interchanged based on our recent DNP work on Cph1 (Stöppler et al., 2016).

Supplementary Table S2. Intramolecular ^{1}H–^{13}C correlations of the PCB chromophore (between $H^{N21–N24}$ and PCB carbon atoms). The data obtained from the ^{1}H–^{13}C MELODI–HETCOR experiments on *u*-[^{13}C,^{15}N]-PCB-*As*.phyA3 as Pr with an LG–CP contact time of 900 μs as well as in the Pfr state with the contact times of 500 and 900 μs, respectively. The Pr data are from Song et al. (2012). The distances of intramolecular ^{1}H–^{13}C contacts ("HM distance") as Pfr were extracted from the corresponding Pfr model. For the 2D ^{1}H–^{13}C MELODI–HETCOR experiments acquired with LG–CP contact times of 500 and 900 μs (displayed in Figure 2A, colored red and purple, respectively), the maximum detection limits for ^{1}H–^{13}C contacts are ~2.8 and ~3.5 Å, respectively, as experimentally determined by the MELODI–HETCOR spectra of *u*-[^{13}C]-labeled tyrosine with an LG–CP contact time varying from 300 μs to 1 ms and as reported previously (Yao et al., 2001; Li and Hong, 2011).

Oat PhyA3–PCB

	Pr				Pfr					
NHs ($H^{N21–N24}$)	δ^{H} (ppm)	cofactor carbon	δ^{C} (ppm)	HM distance (Å)	δ^{H} (ppm)	cofactor carbon	δ^{C} (ppm)	HM distance (Å)	LG–CP contact time 500 μs	900 μs
**H^{N21}**	**12.1**	1	182.4[a]/183.2[b]	2.0	**11.8**	1	182.1	2.1	f.r.†	f.r.
		2	36.9	3.3		-	-	-	-	-
		4	153.1	2.1		4	152.7	2.1	f.r.	f.r.
		5 [a]	85.4	2.7		5	86.7	2.8	f.r.	f.r.
		6	155.9	2.9		6	155.7	3.1	-	s.r.
		-	-	-		9	131.3	3.8	-	s.r.
**H^{N22}**	**10.7**	1 [a]	182.4	3.7	**11.3**	-	-	-	-	-
		-	-	-		4	152.7	3.2	f.r.	f.r.
		5 [b]	86.1	2.9		5	86.7	2.9	-	s.r.
		6	155.9	2.1		6	155.7	2.1	f.r.	f.r.
		-	-	-		7	123.8	3.3	-	s.r.
		8	146.0	3.3		-	-	-	-	-
		9	128.2	2.1		9	131.3	2.2	f.r.	f.r.
		10	113.0	3.0		10	112.0	2.9	s.r.	-
		11	127.4	3.4		11	130.1	3.2	-	s.r.
**H^{N23}**	**10.7**	9	128.2	3.2	**10.3**	9	131.3	3.1	s.r.	s.r.
		10	113.0	2.9		10	112.0	2.8	s.r.	f.r.
		11	127.4	2.1		11	130.1	2.2	f.r.	f.r.
		-	-	-		12	144.7	3.2	-	s.r.
		-	-	-		13	129.3	3.2	-	s.r.
		14	147.1	2.1		14	149.9	2.1	f.r.	f.r.
		15	93.8	2.7		15	93.0	2.7	f.r.	s.r.
**H^{N24}**	**9.8**	13	123.4	2.8	**9.0**	-	-	-	-	-
		13^{1}	10.3	2.1		-	-	-	-	-
		14	147.1	3.1		-	-	-	-	-
		15	93.8	2.9		15	93.0	2.8	f.r.	s.r.
		16	146.3	2.1		16	150.3	2.2	f.r.	s.r.
		17	143.0	3.2		-	-	-	-	-
		-	-	-		18	140.7	3.3	-	f.r.
		19	172.4	2.2		19	169.5	2.1	f.r.	f.r.

†f.r.: fully resolved; s.r.: small but resolved.

Supplementary Table S3. Experimental ^{15}N chemical shifts for the tetrapyrrole nitrogen atoms of the oat phyA3 PCB chromophore as Pfr compared with the computed values using QM/MM model in the corresponding Pfr state. The QM partition of the Pfr-state QM/MM model included 198 atoms (Supplementary Figure S11). The $\Delta\delta^{N}_{exp.-calc.}$ values were reported as the experimental values ($\Delta\delta^{N}_{exp.}$) minus the calculated ones ($\Delta\delta^{N}_{calc.}$).

	Oat PhyA3–PCB Pfr		
pyrrole nitrogen (N21–N24)	$\delta^{N}_{exp.}$ (ppm)	$\delta^{N}_{calc.}$ (ppm)	$\Delta\delta^{N}_{exp.-calc.}$ (ppm)
**N21**	159.0‡	165.3ǁ	**–6.3**
**N22**	147.0<br>142.1	147.0	**0.0**<br>**+1.4**
**N23**	157.3	154.1	**+3.2**
**N24**	136.9	138.0	**–1.1**

‡See Supplementary Table S1; ǁComputed ^{15}N Pfr chemical shifts using QM198/MM Pfr model.

Supplementary Table S4. FWHM line-widths of ^{13}C and ^{15}N resonances ($\nu_{1/2}$) of the PCB chromophore atoms in *u*-[^{13}C,^{15}N]-PCB-*As*.phyA3. $\nu_{1/2}$ values as Pr and Pfr were derived from Song et al. (2012) and the present data, respectively. The ^{13}C and ^{15}N experimental line-shapes are simulated by the Voigt function (convolution of a Lorentzian with a Gaussian at an equal ratio). The $\nu_{1/2}$ values (mean ± standard deviation) were extracted from the Voigt profiles (fitting spectra not shown). $\Delta\nu_{1/2}$ are reported as Pfr minus Pr and visualized in Figure 3B and Supplementary Figure 9.

Oat PhyA3–PCB

cofactor carbon		Pr δ^{C}_{Pr} (ppm)	Pr $\nu_{1/2}^{Pr}$ (FWHM, Hz)	Pfr δ^{C}_{Pfr} (ppm)	Pfr $\nu_{1/2}^{Pfr}$ (FWHM, Hz)	$\Delta\nu_{1/2}^{Pfr-Pr}$ (Hz)
ring A	1	182.4[†](182.6[‡]) (1[a])	286.2±23.9[¶]	182.1[§](182.5[‡])	248.6±15.4[§]	−37.6
		183.2(n.d.) (1[b])	243.8±34.1			+4.8
	2	36.9(36.8)	308.4±20.6	36.3(37.0)	281.4±25.7	−27.0
	2^{1}	17.6(17.7)	418.5±37.3	17.4(17.7)	335.9±28.0	−82.6
	3	53.9(53.9) (3[a])	724.2±38.5	53.9(53.9)	589.4±25.9	−134.8
		55.8(n.d.) (3[b])	657.6±35.0			−68.2
	3^{1}	47.7(45.8) (3^{1a})	482.7±21.2	49.0(49.1)	394.5±20.4	−88.2
		48.9(47.6) (3^{1b})	763.6±36.6			−369.1
	3^{2}	23.0(23.0) (3^{2a})	428.3±36.2	21.5(21.3)	360.8±21.0	−67.5
		21.6(21.7) (3^{2b})	617.0±50.3			−256.2
	4	153.1(n.d.)	341.2±76.6	152.7(n.d.)	206.8±33.1	−134.4
A–B	5	85.4(n.d.) (5[a])	448.3±36.6	86.7(87.1)	298.3±16.6	−150.0
		86.1(85.8) (5[b])	385.9±48.0			−87.6
ring B	6	155.9(n.d.)	308.0±45.4	155.7(n.d.)	201.8±29.9	−106.2
	7	124.3(124.7)	373.7±44.2	123.8(124.6)	412.1±11.7	+38.4
	7^{1}	7.3(7.2)	260.6±22.8	7.3(7.5)	201.7±19.6	−58.9
	8	146.0(146.4)	414.2±47.4	142.9(143.4)	433.6±59.3	+19.4
	8^{1}	22.0(21.8)	508.8±60.3	22.9(23.0)	346.0±37.2	−162.8
	8^{2}	41.4(41.5)	432.5±13.0	41.2(41.1)	387.3±59.0	−45.2
	8^{3}	179.6(179.9)	336.4±17.7	180.1(179.7)	290.5±10.8	−45.9
	9	128.2(127.7)	319.8±32.1	131.3(130.6)	253.2±24.6	−66.6
B–C	10	113.0(113.0)	332.9±15.4	112.0(111.9)	240.1±9.5	−92.8
ring C	11	127.4(127.7)	403.7±19.6	130.1(130.6)	319.4±44.1	−84.3
	12	144.1(146.4)	441.7±41.0	144.7(144.5)	366.1±34.5	−75.6
	12^{1}	22.8(21.8)	421.2±40.1	20.6(21.0)	446.2±58.7	+25.0
	12^{2}	42.1(41.5)	675.4±59.2	38.7(39.0)	441.9±41.0	−233.5
	12^{3}	176.7(n.d.) (12^{3a})	475.5±23.8	174.8(174.7)	408.8±34.1	−66.7
		177.2(177.5) (12^{3b})	335.6±27.4			+73.2
	13	123.4(123.7)	395.4±39.3	129.3(130.1)	347.3±43.8	−48.1
	13^{1}	10.3(10.0)	288.8±67.0	12.8(12.1)	343.9±39.7	+55.1
	14	147.1(146.6)	477.0±36.7	149.9(150.5)	354.5±44.7	−122.5
C–D	15	93.8(93.3)	368.5±17.2	93.0(92.3)	311.7±16.6	−56.8
ring D	16	146.3(146.6)	408.0±97.8	150.3(150.5)	325.5±45.6	−82.5
	17	143.0(142.6)	329.4±41.2	138.5(138.6)	270.2±10.3	−59.2
	17^{1}	10.1(10.3)	251.6±50.4	9.9(10.3)	141.6±6.5	−110.0
	18	133.1(133.3)	298.7±10.7	140.7(140.1)	238.3±31.9	−60.4
	18^{1}	16.7(16.6)	577.9±81.3	16.0(n.d.)	404.0±39.2	−173.9
	18^{2}	12.9(n.d.)	218.0±17.5	13.3(n.d.)	307.6±55.1	+89.6
	19	172.4(172.4)	518.5±22.1	169.5(169.6)	414.2±41.4	−104.3
**pyrrole nitrogen**		δ^{N}_{Pr} (ppm)	$\nu_{1/2}^{Pr}$ (FWHM, Hz)	δ^{N}_{Pfr} (ppm)	$\nu_{1/2}^{Pfr}$ (FWHM, Hz)	$\Delta\nu_{1/2}^{Pfr-Pr}$ (Hz)
ring A	21	156.9[†](158.1[‡])	178.3±14.8[¶]	159.0[§](158.2[‡])	148.7±16.7[§]	−29.6
ring B	22	143.5(144.8)	288.2±16.4	140.7(142.6) (22[a])	161.4±11.8	−126.8
				142.1(143.4) (22[b])	203.0±13.2	−85.2
ring C	23	159.2(160.5)	243.1±20.7	157.3(156.1)	181.6±19.5	−61.5
ring D	24	132.7(131.9)	239.4±15.9	136.9(138.0)	154.1±9.9	−85.3

[†]Song et al., 2012; [‡]Rohmer et al., 2008; [¶]based on Song et al., 2012; [§]present work; n.d.: not determined; ^{15}N Pr assignments of N22 and N23 were interchanged (Stöppler et al., 2016).

Supplementary Table S5. Overview of the interfacial ^{1}H contacts of the chromophore between the PCB carbon atoms and neighboring protons of the protein residues ($^{1}H^{residue}$–$^{13}C^{PCB}$). The MELODI–HETCOR data were obtained from *u*-[^{13}C,^{15}N]-PCB-*As*.phyA3 with an LG–CP contact time of 2.3 ms (effective maximum transfer range of ~5.0 Å) as both Pr and Pfr. Pr data are from Song et al. (2012) and the reassigned Pr resonances were taken from Supplementary Table S10. The ^{1}H–^{13}C distances of interfacial correlations ("HM distance") in both Pr and Pfr states were extracted from the corresponding Pr and Pfr structural models for *As*.phyA3–PCB. ^{1}H NMR chemical shift changes ($\Delta\delta^{H}_{Pfr-Pr}$) of the PCB contacts during Pr-to-Pfr photoconversion are listed at the right-most column as Pfr minus Pr.

Oat PhyA3–PCB

	Pr				Pfr				
cofactor carbon	δ^{C}_{Pr} (ppm)	^{1}H contact (residue)	δ^{H}_{Pr} (ppm)	HM distance (Å)	δ^{C}_{Pfr} (ppm)	^{1}H contact (residue)	δ^{H}_{Pfr} (ppm)	HM distance (Å)	$\Delta\delta^{H}_{Pfr-Pr}$ (ppm)
**1**	**182.4[a]/183.2[b]**	Asp-272 Cα[a]	5.2	3.3	**182.1**	Asp-272 Cα	5.3	3.0	+0.1
		Asp-272 N[a]	12.5	5.8		-	-	-	-
		-	-	-		Met-549 Cε	2.9	3.7	-
		-	-	-		Thr-271 Oγ1	9.7	4.5	-
		Tyr-326 Cβ[a]	4.0	4.5		-	-	-	-
		Tyr-326 Cδ1[b]	10.6	3.5		Tyr-326 Cδ1	10.5	3.8	−0.1
		Tyr-326 Cε1[a]	11.8	4.2		Tyr-326 Cε1	11.7	3.5	−0.1
		W1-**I**	7.0	-		-	-	-	-
		W1-**II**[a]	7.9	3.6		pw	7.8	3.6	−0.1
**2**	**36.9**	Asp-272 Cα	5.2	4.2	**36.3**	Asp-272 Cα	5.3	3.8	+0.1
		-	-	-		Ile-67 Cδ1	1.3	3.9	-
		-	-	-		Met-549 Cε	2.9	4.1	-
		Pro-540 Cβ	3.1	5.3		-	-	-	-
		Pro-551 Cα	4.5	3.4		-	-	-	-
		Pro-551 Cβ	2.2	3.6		-	-	-	-
		-	-	-		Thr-271 Cβ	4.7	4.9	-
		W1-**I**	7.0	-		-	-	-	-
**2^{1}**	**17.6**	Asp-272 N	12.5	5.4	**17.4**	-	-	-	-
		-	-	-		Ile-67 Cγ1	2.2	4.1	-
		Pro-540 Cβ	3.1	3.9		-	-	-	-
		Ser-541 Cα	5.7	4.9		-	-	-	-
		-	-	-		Ser-63 Oγ	6.9	3.9	-
		Thr-271 Cβ	4.8	3.6		Thr-271 Cβ	4.7	3.5	−0.1
**3**	**53.9[a]/55.8[b]**	Cys-322 Cβ	4.5	3.8	**53.9**	-	-	-	-
		-	-	-		Ile-67 Cγ1	2.2	4.4	-
		-	-	-		Ile-67 Cγ2	2.2	4.1	-
		-	-	-		Ile-67 Cδ1	1.3	3.5	-
		Met-549 Cε[b]	2.8	5.0		-	-	-	-
		-	-	-		Pro-274 Cγ	3.7	3.7	-
		-	-	-		Tyr-69 Cε2	10.1	5.0	-
**3^{1}**	**47.7[a]/48.9[b]**	Cys-322 Cα[b]	6.2	4.7	**49.0**	Cys-322 Cα	5.8	4.6	−0.4
		Cys-322 Cβ	4.5	3.0		Cys-322 Cβ	4.9	2.8	+0.4
		Ile-67 Cγ1[b]	3.6	3.9		Ile-67 Cγ1	2.2	4.4	−1.4
		-	-	-		Ile-67 Cγ2	2.2	4.1	-
		Met-549 Cβ[b]	2.6	4.4		-	-	-	-
		Met-549 Cε[b]	2.8	4.1		-	-	-	-
		-	-	-		Tyr-69 Cε2	10.1	4.1	-
**3^{2}**	**23.0[a]/21.6[b]**	Cys-322 Cα[b]	6.2	5.4	**21.5**	-	-	-	-
		Cys-322 Cβ[b]	4.5	3.8		Cys-322 Cβ	4.9	3.4	+0.4
		His-550 N[b]	11.4	4.7		-	-	-	-
		Ile-67 Cγ1[b]	3.6	3.9		Ile-67 Cγ1	2.2	3.3	−1.4
		-	-	-		Ile-67 Cγ2	2.2	3.5	-
		-	-	-		Ile-67 Cδ1	1.3	4.1	-
		Met-549 Cα[b]	5.2	5.1		-	-	-	-
		Met-549 Cβ[b]	2.6	3.2		-	-	-	-
		Met-549 Cε[b]	2.8	3.2		-	-	-	-
		-	-	-		Tyr-69 Cδ2	9.4	4.2	-
		-	-	-		Tyr-69 Cε2	10.1	2.8	-

Oat PhyA3–PCB

cofactor carbon	Pr				Pfr				
	δ^{C}_{Pr} (ppm)	^{1}H contact (residue)	δ^{H}_{Pr} (ppm)	HM distance (Å)	δ^{C}_{Pfr} (ppm)	^{1}H contact (residue)	δ^{H}_{Pfr} (ppm)	HM distance (Å)	$\Delta\delta^{H}_{Pfr-Pr}$ (ppm)
**4**	**153.1**	Asp-272 Cα	5.2	4.5	**152.7**	Asp-272 Cα	5.3	4.0	+0.1
		-	-	-		Cys-322 Cβ	4.9	3.9	-
		-	-	-		Pro-274 Cδ	6.2	3.5	-
		W1-**II**	7.9	3.8		pw	7.8	3.7	−0.1
**5**	**85.4[a]/86.1[b]**	-	-	-	**86.7**	Cys-322 Cβ	4.9	3.7	-
		-	-	-		Ile-67 Cγ2	2.2	4.8	-
		Pro-274 Cγ	4.8	3.5		Pro-274 Cγ	3.7	3.2	−1.1
		Pro-274 Cδ[b]	6.3	3.7		Pro-274 Cδ	6.2	3.1	−0.1
		W1-**I**[a]	7.0	-		-	-	-	-
		W1-**II**	7.9	4.2		-	-	-	-
**6**	**155.9**	His-323 Cα	5.5	4.3	**155.7**	His-323 Cα	5.4	4.5	−0.1
		-	-	-		-	-	-	-
		His-323 N	11.7	4.2		-	-	-	-
		Pro-274 Cγ	4.8	3.0		Pro-274 Cγ	3.7	3.5	−1.1
		Pro-274 Cδ	6.3	3.4		Pro-274 Cδ	6.2	3.1	−0.1
		-	-	-		pw	7.8	3.8	-
**7**	**124.3**	His-320 Cβ	3.2	3.6	**123.8**	-	-	-	-
		His-323 Cα	5.5	4.9		-	-	-	-
		His-323 Cβ	4.0	3.1		His-323 Cβ	4.3	3.2	+0.3
		-	-	-		Pro-274 Cβ	2.9	5.2	-
		Pro-274 Cγ	4.8	3.0		Pro-274 Cγ	3.7	3.5	−1.1
		-	-	-		Pro-274 Cδ	6.2	3.4	-
**7^1**	**7.3**	Cys-322 Cβ	4.5	3.6	**7.3**	-	-	-	-
		-	-	-		His-320 Cα	5.0	4.8	-
		His-320 Cβ	3.2	2.8		-	-	-	-
		-	-	-		His-320 N	12.4	4.7	-
		-	-	-		His-323 Cβ	4.3	3.9	-
		His-323 N	11.7	4.3		His-323 N	11.4	4.9	−0.3
		Pro-274 Cγ	4.8	3.1		-	-	-	-
**8**	**146.0**	Ala-277 Cβ	2.0	4.1	**142.9**	-	-	-	-
		-	-	-		His-323 Cα	5.4	5.1	-
		His-323 Cβ	4.0	3.9		His-323 Cβ	4.3	3.2	+0.3
		-	-	-		His-323 Cε1	12.3	5.0	-
		-	-	-		Phe-281 Cζ	7.1	4.6	-
		Pro-274 Cδ	6.3	4.8		-	-	-	-
**8^1**	**22.0**	-	-	-	**22.9**	Ala-277 Cβ	2.3	3.8	-
		-	-	-		Arg-287 Nε	10.6	4.2	-
		-	-	-		Arg-287 Nη2	9.9	4.9	-
		Arg-317 Nη1	10.0	4.4		-	-	-	-
		His-320 Cβ	3.2	3.4		-	-	-	-
		His-320 N	12.0	3.1		-	-	-	-
		His-323 Cδ2-**II**	12.8	4.9		-	-	-	-
		-	-	-		Phe-281 Cζ	7.1	3.9	-
		Pro-319 Cβ	3.2	3.7		Pro-319 Cβ	2.9	4.7	−0.3
**8^2**	**41.4**	Ala-277 Cα	3.8	4.5	**41.2**	-	-	-	-
		-	-	-		Ala-277 Cβ	2.3	4.5	-
		-	-	-		Arg-287 Nε	10.6	3.9	-
		-	-	-		Arg-287 Nη2	9.9	3.9	-
		-	-	-		Arg-317 Nη1	11.7	4.0	-
		-	-	-		His-320 N	12.4	3.8	-
		Pro-319 Cα	5.7	5.0		Pro-319 Cα	5.8	4.2	+0.1
		Pro-319 Cβ	3.2	4.6		Pro-319 Cβ	2.9	3.8	−0.3

Oat PhyA3–PCB

	Pr				Pfr				
cofactor carbon	δ^{C}_{Pr} (ppm)	^{1}H contact (residue)	δ^{H}_{Pr} (ppm)	HM distance (Å)	δ^{C}_{Pfr} (ppm)	^{1}H contact (residue)	δ^{H}_{Pfr} (ppm)	HM distance (Å)	$\Delta\delta^{H}_{Pfr-Pr}$ (ppm)
**8^3**	**179.6**	Ala-277 Cα	3.8	4.5	**180.1**	-	-	-	-
		Ala-277 Cβ	2.0	3.8		-	-	-	-
		-	-	-		Arg-287 Cδ	5.0	4.6	-
		-	-	-		Arg-287 Nε	10.6	2.6	-
		Arg-287 Nη1	9.2	7.4		-	-	-	-
		-	-	-		Arg-287 Nη2	9.9	2.4	-
		Arg-317 Nη1	10.0	2.4		Arg-317 Nη1	11.7	3.1	+1.7
		Arg-317 Nη2	7.2	2.5		Arg-317 Nη2	8.5	4.5	+1.3
		His-320 N	12.0	3.5		His-320 N	12.4	4.0	+0.4
		-	-	-		Phe-281 Cε1	7.7	4.5	-
		Pro-319 Cα	5.7	3.9		Pro-319 Cα	5.8	3.5	+0.1
		Pro-319 Cβ	3.2	4.4		Pro-319 Cβ	2.9	3.1	−0.3
**9**	**128.2**	Ala-277 Cβ	2.0	4.7	**131.3**	-	-	-	-
		His-323 Cα	5.5	4.3		His-323 Cα	5.4	4.8	−0.1
		His-323 Cβ	4.0	2.5		His-323 Cβ	4.3	3.5	+0.3
		His-323 Cε1-**I**	13.6	-		-	-	-	-
		His-323 Nδ1-**I**	16.9	-		-	-	-	-
		His-323 N	11.0	4.6		His-323 N	11.4	5.8	+0.4
		-	-	-		Ile-273 Cα	4.8	4.2	-
		-	-	-		Pro-274 Cδ	6.2	3.7	-
		-	-	-		pw	7.8	3.9	-
**10**	**113.0**	His-323 Cβ	4.0	3.1	**112.0**	-	-	-	-
		His-323 Cδ2-**I**	16.1	-		-	-	-	-
		His-323 Cε1-**I**	13.6	-		-	-	-	-
		His-323 Cε1-**II**	12.1	4.0		His-323 Cε1	12.3	4.3	+0.2
		His-323 Nε2-**II**	14.7	4.4		-	-	-	-
		Ile-273 Cα	4.4	4.4		Ile-273 Cα	4.8	4.5	+0.2
		-	-	-		Ile-273 Cδ1	2.0	3.2	-
		-	-	-		Phe-281 Cζ	7.1	4.5	-
		W1-**I**	7.0	-		-	-	-	-
		W1-**II**	7.9	4.2		-	-	-	-
**11**	**127.4**	Ala-277 Cβ	2.0	4.8	**130.1**	-	-	-	-
		His-323 Cδ2-**I**	16.1	-		-	-	-	-
		His-323 Cδ2-**II**	12.8	4.9		-	-	-	-
		His-323 Cε1-**II**	12.1	3.2		His-323 Cε1	12.3	4.2	+0.2
		His-323 Nδ1-**I**	16.9	-		-	-	-	-
		His-323 Nε2-**I**	18.4	-		-	-	-	-
		Ile-273 Cα	4.4	4.0		-	-	-	-
		W1-**II**	7.9	3.9		-	-	-	-
**12**	**144.1**	His-323 Cδ2-**I**	16.1	-	**144.7**	-	-	-	-
		His-323 Cδ2-**II**	12.8	5.1		-	-	-	-
		His-323 Cε1-**I**	13.6	-		-	-	-	-
		His-323 Cε1-**II**	12.1	3.2		-	-	-	-
		His-323 Nε2-**II**	14.7	3.7		-	-	-	-
		Ile-273 Cγ1	1.0	3.8		-	-	-	-
		-	-	-		Ile-273 Cδ1	2.0	3.0	-
		Phe-281 Cζ	7.2	4.2		-	-	-	-
		-	-	-		Val-337 Cγ1	1.1	4.9	-
**12^1**	**22.8**	Ala-277 Cβ	2.0	4.3	**20.6**	-	-	-	-
		-	-	-		His-323 Cε1	12.3	3.9	-
		His-323 Nε2-**II**	14.7	3.7		-	-	-	-
		Phe-281 Cζ	7.2	3.2		-	-	-	-
		-	-	-		Val-337 Cγ1	1.1	3.4	-
		Val-337 Cγ2	1.3	4.1		-	-	-	-
		{W5+[OH]$^{-}_{W4}$} (**Pr-I**)	9.6	-		-	-	-	-

Oat PhyA3–PCB

	Pr				Pfr				
cofactor carbon	δ^{C}_{Pr} (ppm)	^{1}H contact (residue)	δ^{H}_{Pr} (ppm)	HM distance (Å)	δ^{C}_{Pfr} (ppm)	^{1}H contact (residue)	δ^{H}_{Pfr} (ppm)	HM distance (Å)	$\Delta\delta^{H}_{Pfr-Pr}$ (ppm)
**12^{2}**	**42.1**	Arg-287 Cβ	2.8	4.7	**38.7**	-	-	-	-
		His-323 Cε1-**II**	12.1	4.2		-	-	-	-
		His-323 Nδ1-**I**	16.9	-		-	-	-	-
		-	-	-		His-372 Nε2	12.8	3.8	-
		Leu-368 Cδ1	1.7	4.9		-	-	-	-
		Phe-281 Cζ	7.2	3.1		-	-	-	-
		-	-	-		Ser-335 Cβ	4.0	4.6	-
		-	-	-		Val-337 Cβ	2.3	4.0	-
		Val-337 Cγ1	0.9	4.0		Val-337 Cγ1	1.1	3.6	**+0.2**
		Val-337 Cγ2	1.3	2.7		-	-	-	-
		W3	8.1	4.4		-	-	-	-
		W4 (**Pr-II**)	8.9	4.4		-	-	-	-
**12^{3}**	**176.7[a]/177.2[b]**	Arg-287 Cβ[a]	2.8	4.1	**174.8**	-	-	-	-
		His-323 Cε1-**I**[b]	13.6	-		-	-	-	-
		His-323 Nδ1-**I**[b]	16.9	-		-	-	-	-
		His-323 Nε2-**II**[a]	14.7	2.4		-	-	-	-
		-	-	-		His-372 Cδ2	11.5	4.0	-
		-	-	-		His-372 Cε1	9.9	4.3	-
		-	-	-		His-372 Nε2	12.8	2.3	-
		-	-	-		Met-330 Cε	3.3	4.6	-
		Phe-281 Cζ	7.2	3.2		-	-	-	-
		-	-	-		Ser-335 Cβ	4.0	4.1	-
		-	-	-		Val-337 Cβ	2.3	4.1	-
		Val-337 Cγ2[a]	1.3	3.4		-	-	-	-
		-	-	-		Val-370 Cγ1	1.1	3.9	-
		W2[b]	8.0	3.3		-	-	-	-
		W3	8.1	3.3		-	-	-	-
		W4 (**Pr-II**)[b]	8.9	3.9		-	-	-	-
		W5 (**Pr-II**)[a]	8.8	-		-	-	-	-
**13**	**123.4**	His-323 Cδ2-**I**	16.1	-	**129.3**	-	-	-	-
		His-323 Nε2-**I**	18.4	-		-	-	-	-
		Ile-273 Cδ1	1.8	4.2		Ile-273 Cδ1	2.0	3.1	**+0.2**
		-	-	-		Tyr-241 Cε2	8.8	5.0	-
		-	-	-		Val-370 Cγ1	1.1	4.2	-
		W5 (**Pr-II**)	8.8	-		-	-	-	-
		$\{W5+[OH]^{-}_{W4}\}$ (**Pr-I**)	9.6	-		-	-	-	-
**13^{1}**	**10.3**	His-323 Nε2-**I**	18.4	-	**12.8**	-	-	-	-
		His-323 Nε2-**II**	14.7	5.0		-	-	-	-
		-	-	-		His-372 Nε2	12.8	3.9	-
		Ile-273 Cδ1	1.8	3.6		Ile-273 Cδ1	2.0	4.2	**+0.2**
		-	-	-		Leu-368 Cβ	2.9	4.5	-
		Leu-368 Cδ1	1.7	3.2		-	-	-	-
		-	-	-		Tyr-241 Cε2	8.8	3.6	-
		Val-337 Cγ1	0.9	4.2		-	-	-	-
		Val-370 Cβ	2.2	4.4		-	-	-	-
		Val-370 Cγ1	0.9	4.8		-	-	-	-
**14**	**147.1**	-	-	-	**149.9**	Asp-272 Cβ	2.9	4.7	-
		His-323 Cε1-**II**	12.1	3.0		-	-	-	-
		-	-	-		Ile-273 Cα	4.8	4.7	-
		Ile-273 Cγ1	1.0	2.6		Ile-273 Cγ1	1.4	3.2	**+0.4**
		Ile-273 Cδ1	1.8	4.2		Ile-273 Cδ1	2.0	2.9	**+0.2**
		-	-	-		Tyr-326 Cε2	10.8	4.5	-
		$\{W5+[OH]^{-}_{W4}\}$ (**Pr-I**)	9.6	-		-	-	-	-

	Oat PhyA3–PCB								
	Pr				Pfr				
cofactor carbon	δ^{C}_{Pr} (ppm)	¹H contact (residue)	δ^{H}_{Pr} (ppm)	HM distance (Å)	δ^{C}_{Pfr} (ppm)	¹H contact (residue)	δ^{H}_{Pfr} (ppm)	HM distance (Å)	$\Delta\delta^{H}_{Pfr-Pr}$ (ppm)
**15**	**93.8**	Asp-272 Cβ	3.5	3.6	**93.0**	Asp-272 Cβ	2.9	3.8	−0.6
		His-323 Cε1-**I**	13.6	-		-	-	-	-
		His-323 Cε1-**II**	12.1	3.7		-	-	-	-
		-	-	-		Ile-273 Cα	4.8	4.7	-
		Ile-273 Cγ1	1.0	2.6		Ile-273 Cγ1	1.4	2.8	+0.4
		Ile-273 Cδ1	1.8	3.6		Ile-273 Cδ1	2.0	3.5	+0.2
		Tyr-326 Cε2	11.2	4.3		Tyr-326 Cε2	10.8	4.3	−0.4
		Tyr-326 Oη	8.3	4.9		Tyr-326 Oη	10.0	4.6	+1.7
		W1-**II**	7.9	3.7		-	-	-	-
**16**	**146.3**	-	-	-	**150.3**	Asp-272 Cβ	2.9	4.3	-
		-	-	-		-	-	-	-
		Ile-273 Cγ1	1.0	3.6		Ile-273 Cγ1	1.4	2.8	+0.4
		-	-	-		Tyr-268 Cβ	3.7	4.1	-
		Tyr-326 Oη	8.3	4.6		Tyr-326 Oη	10.0	4.7	+1.7
		{W5+$[OH]^{-}_{W4}$}(**Pr-I**)	9.6	-		-	-	-	-
**17**	**143.0**	-	-	-	**138.5**	Ile-273 Cγ1	1.4	3.3	-
		-	-	-		Ile-273 Cδ1	2.0	3.9	-
		-	-	-		Leu-368 Cδ1	1.8	3.6	-
		-	-	-		Phe-243 Cε1	7.9	4.6	-
		-	-	-		Phe-243 Cζ	7.0	4.2	-
		-	-	-		Tyr-241 Cδ2	8.6	3.3	-
		-	-	-		Tyr-268 Cβ	3.7	4.5	-
		Tyr-326 Cε2	11.2	4.1		-	-	-	-
		Tyr-326 Oη	8.3	4.3		-	-	-	-
**17^1**	**10.1**	Asp-272 Cβ	3.5	2.9	**9.9**	-	-	-	-
		-	-	-		Ile-273 Cγ1	1.4	3.7	-
		-	-	-		Ile-273 Cδ1	2.0	3.5	-
		-	-	-		Leu-368 Cβ	2.9	3.3	-
		-	-	-		Leu-368 Cδ1	1.8	2.8	-
		-	-	-		Phe-243 Cε1	7.9	5.3	-
		-	-	-		Phe-243 Cζ	7.0	4.2	-
		-	-	-		Tyr-241 Cβ	3.2	4.3	-
		-	-	-		Tyr-241 Cδ2	8.6	2.8	-
		Tyr-241 Cε1	9.0	5.0		-	-	-	-
		-	-	-		Tyr-241 Cε2	8.8	4.4	-
		Tyr-268 Cε1	9.6	5.1		-	-	-	-
		Tyr-326 Cε1	11.8	5.0		-	-	-	-
		Tyr-326 Oη	8.3	4.0		-	-	-	-
**18**	**133.1**	-	-	-	**140.7**	Gly-249 Cα	4.5	4.3	-
		-	-	-		Gly-249 N	11.7	5.7	-
		His-372 Nε2 (**Pr-II**)	12.2	4.7		-	-	-	-
		-	-	-		Ile-273 Cγ1	1.4	4.5	-
		-	-	-		Leu-368 Cδ1	1.8	4.1	-
		-	-	-		Phe-243 Cε1	7.9	3.8	-
		Tyr-241 Cδ1	10.4	5.6		-	-	-	-
		-	-	-		Tyr-241 Cδ2	8.6	3.6	-
		-	-	-		Tyr-268 Cβ	3.7	3.8	-
		-	-	-		Tyr-268 Cε2	10.5	4.8	-
		Tyr-268 Cε1	9.6	5.7		-	-	-	-

Oat PhyA3–PCB

	Pr				Pfr				
cofactor carbon	δ^{C}_{Pr} (ppm)	^{1}H contact (residue)	δ^{H}_{Pr} (ppm)	HM distance (Å)	δ^{C}_{Pfr} (ppm)	^{1}H contact (residue)	δ^{H}_{Pfr} (ppm)	HM distance (Å)	$\Delta\delta^{H}_{Pfr-Pr}$ (ppm)
**18^{1}**	**16.7**	-	-	-	**16.0**	Gly-249 Cα	4.5	3.4	-
		-	-	-		Phe-243 Cε1	7.9	3.7	-
		-	-	-		Phe-243 Cζ	7.0	4.0	-
		Met-239 Cε	3.1	3.8		-	-	-	-
		Tyr-241 Cβ	3.8	4.3		Tyr-241 Cβ	3.2	3.2	**–0.6**
		Tyr-241 Cδ1	10.4	4.3		-	-	-	-
		-	-	-		Tyr-241 Cδ2	8.6	3.4	-
		Tyr-241 Cε1	9.0	4.5		-	-	-	-
		Tyr-268 Cε1	9.6	5.3		-	-	-	-
		Tyr-268 Cε2	9.7	4.3		-	-	-	-
		Tyr-268 Oη	8.0	5.1		-	-	-	-
		Val-251 Cγ2	1.1	4.2		-	-	-	-
**18^{2}**	**12.9**	-	-	-	**13.3**	Glu-250 N	10.8	3.9	-
		-	-	-		Gly-249 Cα	4.5	2.8	-
		-	-	-		Gly-249 N	11.7	4.7	-
		-	-	-		Phe-243 Cε1	7.9	4.7	-
		Met-239 Cε	3.1	3.5		-	-	-	-
		Tyr-241 Cβ	3.8	2.8		-	-	-	-
		Tyr-241 Cδ1	10.4	3.0		-	-	-	-
		Tyr-241 Cε1	9.0	3.9		-	-	-	-
		Tyr-268 Cε2	9.7	4.7		-	-	-	-
		Tyr-268 Oη	8.0	5.9		-	-	-	-
		Val-251 Cγ2	1.1	3.5		-	-	-	-
**19**	**172.4**	-	-	-	**169.5**	Asp-272 Cβ	2.9	4.5	-
		His-372 Cε1 (**Pr-I**)	10.4	-		-	-	-	-
		His-372 Cε1 (**Pr-II**)	11.4	4.8		-	-	-	-
		His-372 Nε2 (**Pr-II**)	12.2	3.3		-	-	-	-
		Met-239 Cε	3.1	5.4		-	-	-	-
		-	-	-		Ser-554 Cβ	5.2	4.9	-
		-	-	-		Ser-554 Oγ	6.8	5.3	-
		Val-370 Cβ	2.2	5.0		-	-	-	-
		Val-370 Cγ1	0.9	2.8		-	-	-	-
		W5 (**Pr-II**)	8.8	-		-	-	-	-
		{W5+$[OH]^{-}_{W4}$} (**Pr-I**)	9.6	-		-	-	-	-

Supplementary Table S6. The intermolecular ^{13}C chromophore contacts of the protein residues (cut-off distance ~5.0 Å). The table corresponds to the intermolecular $^{1}H^{residue}$–$^{13}C^{PCB}$ contacts summarized in Supplementary Table S5. ^{1}H NMR chemical shift changes ($\Delta\delta^{H}_{Pfr-Pr}$) of the observed protein residues during Pr-to-Pfr photoconversion are listed at the right-most column. The nearest chromophore carbon contacts of a proton attached to the protein residues ($^{1}H^{residue}$) are labeled in green (see also Supplementary Table S9).

Oat PhyA3–PCB

		Pr				Pfr				
^{1}H contact (residue)		δ^{H}_{Pr} (ppm)	cofactor carbon	δ^{C}_{Pr} (ppm)	HM distance (Å)	δ^{H}_{Pfr} (ppm)	cofactor carbon	δ^{C}_{Pfr} (ppm)	HM distance (Å)	$\Delta\delta^{H}_{Pfr-Pr}$ (ppm)
**Ala-277**	Cα	3.8	8^2	41.4	4.5	-	-	-	-	-
		3.8	8^3	179.6	4.5	-	-	-	-	
	Cβ	2.0	8	146.0	4.1	-	-	-	-	**+0.3**
		-	-	-	-	2.3	8^1	22.9	3.8	
		-	-	-	-	2.3	8^2	41.2	4.5	
		2.0	8^3	179.6	3.8	-	-	-	-	
		2.0	9	128.2	4.7	-	-	-	-	
		2.0	11	127.4	4.8	-	-	-	-	
		2.0	12^1	22.8	4.3	-	-	-	-	
**Arg-287**	Cβ	2.8	12^2	42.1	4.7	-	-	-	-	-
		2.8	12^{3a}	176.7	4.1	-	-	-	-	
	Cδ	-	-	-	-	5.0	8^3	180.1	4.6	-
	Nε	-	-	-	-	10.6	8^1	22.9	4.2	-
		-	-	-	-	10.6	8^2	41.2	3.9	
		-	-	-	-	10.6	8^3	180.1	2.6	
	Nη1	9.2	8^3	179.6	7.4	-	-	-	-	
	Nη2	-	-	-	-	9.9	8^1	22.9	4.9	-
		-	-	-	-	9.9	8^2	41.2	3.9	
		-	-	-	-	9.9	8^3	180.1	2.4	
**Arg-317**	Nη1	10.0	8^1	22.0	4.4	-	-	-	-	**+1.7**
		-	-	-	-	11.7	8^2	41.2	4.0	
		10.0	8^3	179.6	2.4	11.7	8^3	180.1	3.1	
	Nη2	7.2	8^3	179.6	2.5	8.5	8^3	180.1	4.5	**+1.3**
**Asp-272**	Cα	5.2	1^{a}	182.4	3.3	5.3	1^1	182.1	3.0	**+0.1**
		5.2	2	36.9	4.2	5.3	2	36.3	3.8	
		5.2	4	153.1	4.5	5.3	4	152.7	4.0	
	Cβ	-	-	-	-	2.9	14	149.9	4.7	**−0.6**
		3.5	15	93.8	3.6	2.9	15^1	93.0	3.8	
		-	-	-	-	2.9	16	150.3	4.3	
		3.5	17^1	10.1	2.9	-	-	-	-	
		-	-	-	-	2.9	19	169.5	4.5	
	N	12.5	1^{a}	182.4	5.8	-	-	-	-	-
		12.5	2^1	17.6	5.4	-	-	-	-	
**Cys-322**	Cα	6.2	3^{1b}	48.9	4.7	5.8	3^1	49.0	4.6	**−0.4**
		6.2	3^{2b}	21.6	5.4	-	-	-	-	
	Cβ	4.5	3	53.9/55.8	3.8	-	-	-	-	**+0.4**
		4.5	3^1	47.7/48.9	3.0	4.9	3^1	49.0	2.8	
		4.5	3^{2b}	21.6	3.8	4.9	3^2	21.5	3.4	
		-	-	-	-	4.9	4	152.7	3.9	
		-	-	-	-	4.9	5	86.7	3.7	
		4.5	7^1	7.3	3.6	-	-	-	-	
**Glu-250**	N	-	-	-	-	10.8	18^2	13.3	3.9	-
**Gly-249**	Cα	-	-	-	-	4.5	18	140.7	4.3	-
		-	-	-	-	4.5	18^1	16.0	3.4	
		-	-	-	-	4.5	18^2	13.3	2.8	
	N	-	-	-	-	11.7	18	140.7	5.7	-
		-	-	-	-	11.7	18^2	13.3	4.7	
**His-320***	Cα	-	-	-	-	5.0	7^1	7.3	4.8	-

*Continued on following page.

Oat PhyA3–PCB

^{1}H contact (residue)		Pr: δ^H_{Pr} (ppm)	Pr: cofactor carbon	Pr: δ^C_{Pr} (ppm)	Pr: HM distance (Å)	Pfr: δ^H_{Pfr} (ppm)	Pfr: cofactor carbon	Pfr: δ^C_{Pfr} (ppm)	Pfr: HM distance (Å)	$\Delta\delta^H_{Pfr-Pr}$ (ppm)
**His-320**	Cβ	3.2	7	124.3	3.6	-	-	-	-	-
		3.2	7^1	7.3	2.8	-	-	-	-	
		3.2	8^1	22.0	3.4	-	-	-	-	
	N	-	-	-	-	12.4	7^1	7.3	4.7	+0.4
		12.0	8^1	22.0	3.1	-	-	-	-	
		-	-	-	-	12.4	8^2	41.2	3.8	
		12.0	8^3	179.6	3.5	12.4	8^3	180.1	4.0	
**His-323**	Cα	5.5	6	155.9	4.3	5.4	6^1	155.7	4.5	−0.1
		5.5	7	124.3	4.9	-	-	-	-	
		-	-	-	-	5.4	8	142.9	5.1	
		5.5	9	128.2	4.3	5.4	9	131.3	4.8	
	Cβ	4.0	7	124.3	3.1	4.3	7	123.8	3.2	+0.3
		-	-	-	-	4.3	7^1	7.3	3.9	
		4.0	8	146.0	3.9	4.3	8^1	142.9	3.2	
		4.0	9	128.2	2.5	4.3	9	131.3	3.5	
		4.0	10	113.0	3.1	-	-	-	-	
	Cδ2-**I**	16.1	10	113.0	-	-	-	-	-	-
		16.1	11	127.4	-	-	-	-	-	
		16.1	12	144.1	-	-	-	-	-	
		16.1	13	123.4	-	-	-	-	-	
	Cδ2-**II**	12.8	8^1	22.0	4.9	-	-	-	-	-
		12.8	11	127.4	4.9	-	-	-	-	
		12.8	12	144.1	5.1	-	-	-	-	
	Cε1-**I**	13.6	9	128.2	-	-	-	-	-	-
		13.6	10	113.0	-	-	-	-	-	
		13.6	12	144.1	-	-	-	-	-	
		13.6	12^{3b}	177.2	-	-	-	-	-	
		13.6	15	93.8	-	-	-	-	-	
	Cε1-**II**	-	-	-	-	12.3	8	142.9	5.0	+0.2
		12.1	10	113.0	4.0	12.3	10	112.0	4.3	
		12.1	11	127.4	3.2	12.3	11	130.1	4.2	
		12.1	12	144.1	3.2	-	-	-	-	
		-	-	-	-	12.3	12^1	20.6	3.9	
		12.1	12^2	42.1	4.2	-	-	-	-	
		12.1	14	147.1	3.0	-	-	-	-	
		12.1	15	93.8	3.7	-	-	-	-	
	N	11.7	6	155.9	4.2	-	-	-	-	−0.3
		11.7	7^1	7.3	4.3	11.4	7^1	7.3	4.9	
		11.7	9	128.2	4.6	11.4	9	131.3	5.8	
	Nδ1-**I**	16.9	11	127.4	-	-	-	-	-	-
		16.9	12^2	42.1	-	-	-	-	-	
		16.9	12^{3b}	177.2	-	-	-	-	-	
	Nε2-**I**	18.4	11	127.4	-	-	-	-	-	-
		18.4	13	123.4	-	-	-	-	-	
		18.4	13^1	10.3	-	-	-	-	-	
	Nε2-**II**	14.7	10	113.0	4.4	-	-	-	-	
		14.7	12	144.1	3.7	-	-	-	-	
		14.7	12^1	22.8	3.7	-	-	-	-	
		14.7	12^{3a}	176.7	2.4	-	-	-	-	
		14.7	13^1	10.3	5.0	-	-	-	-	
**His-372***	Cδ2	-	-	-	-	11.5	12^3	174.8	4.0	-
	Cε1-**I**	10.4	19	172.4	-	-	-	-	-	-
	Cε1-**II**	-	-	-	-	9.9	12^3	174.8	4.3	−1.5
		11.4	19	172.4	4.8	-	-	-	-	

*Continued on following page.

Oat PhyA3–PCB

[1]H contact (residue)		Pr				Pfr				
		δ^{H}_{Pr} (ppm)	cofactor carbon	δ^{C}_{Pr} (ppm)	HM distance (Å)	δ^{H}_{Pfr} (ppm)	cofactor carbon	δ^{C}_{Pfr} (ppm)	HM distance (Å)	$\Delta\delta^{H}_{Pfr-Pr}$ (ppm)
**His-372**	Nε2-**II**	-	-	-	-	12.8	12^2	38.7	3.8	+0.6
		-	-	-	-	12.8	12^3	174.8	2.3	
		-	-	-	-	12.8	13^1	12.8	3.9	
		12.2	18	133.1	4.7	-	-	-	-	
		12.2	19	172.4	3.3	-	-	-	-	
**His-550**	N	11.4	3^{2b}	21.6	4.7	-	-	-	-	-
**Ile-273**	Cα	-	-	-	-	4.8	9^1	131.3	4.2	+0.5
		4.3	10	113.0	4.4	4.8	10	112.0	4.5	
		4.3	11	127.4	4.0	-	-	-	-	
		-	-	-	-	4.8	14	149.9	4.7	
		-	-	-	-	4.8	15	93.0	4.7	
	Cγ1	1.0	12	144.1	3.8	-	-	-	-	+0.4
		1.0	14	147.1	2.6	1.4	14	149.9	3.2	
		1.0	15	93.8	2.6	1.4	15^1	93.0	2.8	
		1.0	16	146.3	3.6	1.4	16	150.3	2.8	
		-	-	-	-	1.4	17	138.5	3.3	
		-	-	-	-	1.4	17^1	9.9	3.7	
		-	-	-	-	1.4	18	140.7	4.5	
	Cδ1	-	-	-	-	2.0	10	112.0	3.2	+0.2
		-	-	-	-	2.0	12	144.7	3.0	
		1.8	13	123.4	4.2	2.0	13	129.3	3.1	
		1.8	13^1	10.3	3.6	2.0	13^1	12.8	4.2	
		1.8	14	147.1	4.2	2.0	14^1	149.9	2.9	
		1.8	15	93.8	3.6	2.0	15	93.0	3.5	
		-	-	-	-	2.0	17	138.5	3.9	
		-	-	-	-	2.0	17^1	9.9	3.5	
**Ile-67**	Cγ1	-	-	-	-	2.2	2^1	17.4	4.1	−1.4
		-	-	-	-	2.2	3	53.9	4.4	
		3.6	3^{1b}	48.9	3.9	2.2	3^1	49.0	4.4	
		3.6	3^{2b}	21.6	3.9	2.2	3^2	21.5	3.3	
	Cγ2	-	-	-	-	2.2	3	53.9	4.1	-
		-	-	-	-	2.2	3^1	49.0	4.1	
		-	-	-	-	2.2	3^2	21.5	3.5	
		-	-	-	-	2.2	5	86.7	4.8	
	Cδ1	-	-	-	-	1.3	2	36.3	3.9	-
		-	-	-	-	1.3	3^1	53.9	3.5	
		-	-	-	-	1.3	3^2	21.5	4.1	
**Leu-368**	Cβ	-	-	-	-	2.9	13^1	12.8	4.5	-
		-	-	-	-	2.9	17^1	9.9	3.3	
	Cδ1	1.7	12^2	42.1	4.9	-	-	-	-	+0.1
		1.7	13^1	10.3	3.2	-	-	-	-	
		-	-	-	-	1.8	17	138.5	3.6	
		-	-	-	-	1.8	17^1	9.9	2.8	
		-	-	-	-	1.8	18	140.7	4.1	
**Met-239**	Cε	3.1	18^1	16.7	3.8	-	-	-	-	-
		3.1	18^2	12.9	3.5	-	-	-	-	
		3.1	19	172.4	5.4	-	-	-	-	
**Met-330**	Cε	-	-	-	-	3.3	12^3	174.8	4.6	-
**Met-549***	Cα	5.2	3^{2b}	21.6	5.1	-	-	-	-	-
	Cβ	2.6	3^{1b}	48.9	4.4	-	-	-	-	-
		2.6	3^{2b}	21.6	3.2	-	-	-	-	

*Continued on following page.

		Oat PhyA3–PCB								
		Pr				Pfr				
^{1}H contact (residue)		δ^{H}_{Pr} (ppm)	cofactor carbon	δ^{C}_{Pr} (ppm)	HM distance (Å)	δ^{H}_{Pfr} (ppm)	cofactor carbon	δ^{C}_{Pfr} (ppm)	HM distance (Å)	$\Delta\delta^{H}_{Pfr-Pr}$ (ppm)
**Met-549**	Cε	-	-	-	-	2.9	1^{1}	182.1	3.7	+0.1
		-	-	-	-	2.9	2	36.3	4.1	
		2.8	3^{b}	55.8	5.0	-	-	-	-	
		2.8	3^{1b}	48.9	4.1	-	-	-	-	
		2.8	3^{2b}	21.6	3.2	-	-	-	-	
**Phe-243**	Cε1	-	-	-	-	7.9	17	138.5	4.6	-
		-	-	-	-	7.9	17^{1}	9.9	5.3	
		-	-	-	-	7.9	18	140.7	3.8	
		-	-	-	-	7.9	18^{1}	16.0	3.7	
		-	-	-	-	7.9	18^{2}	13.3	4.7	
	Cζ	-	-	-	-	7.0	17	138.5	4.2	-
		-	-	-	-	7.0	17^{1}	9.9	4.2	
		-	-	-	-	7.0	18^{1}	16.0	4.0	
**Phe-281**	Cε1	-	-	-	-	7.7	8^{3}	180.1	4.5	-
	Cζ	-	-	-	-	7.1	8	142.9	4.6	−0.1
		-	-	-	-	7.1	8^{1}	22.9	3.9	
		-	-	-	-	7.1	10	112.0	4.5	
		7.2	12	144.1	4.2	-	-	-	-	
		7.2	12^{1}	22.8	3.2	-	-	-	-	
		7.2	12^{2}	42.1	3.1	-	-	-	-	
		7.2	12^{3}	176.7/177.2	3.2	-	-	-	-	
**Pro-274**	Cβ	-	-	-	-	2.9	7	123.8	5.2	-
	Cγ	-	-	-	-	3.7	3	53.9	3.7	−1.1
		4.8	5	85.4/86.1	3.5	3.7	5^{1}	86.7	3.2	
		4.8	6	155.9	3.0	3.7	6	155.7	3.5	
		4.8	7	124.3	3.0	3.7	7	123.8	3.5	
		4.8	7^{1}	7.3	3.1	-	-	-	-	
	Cδ	-	-	-	-	6.2	4	152.7	3.5	−0.1
		6.3	5^{b}	86.1	3.7	6.2	5	86.7	3.1	
		6.3	6	155.9	3.4	6.2	6^{1}	155.7	3.1	
		-	-	-	-	6.2	7	123.8	3.4	
		6.3	8	146.0	4.8	-	-	-	-	
		-	-	-	-	6.2	9	131.3	3.7	
**Pro-319**	Cα	5.7	8^{2}	41.4	5.0	5.8	8^{2}	41.2	4.2	+0.1
		5.7	8^{3}	179.6	3.9	5.8	8^{3}	180.1	3.5	
	Cβ	3.2	8^{1}	22.0	3.7	2.9	8^{1}	22.9	4.7	−0.3
		3.2	8^{2}	41.4	4.6	2.9	8^{2}	41.2	3.8	
		3.2	8^{3}	179.6	4.4	2.9	8^{3}	180.1	3.1	
**Pro-540**	Cβ	3.1	2	36.9	5.3	-	-	-	-	-
		3.1	2^{1}	17.6	3.9	-	-	-	-	
**Pro-551**	Cα	4.5	2	36.9	3.4	-	-	-	-	-
	Cβ	2.2	2	36.9	3.6	-	-	-	-	-
**Ser-335**	Cβ	-	-	-	-	4.0	12^{2}	38.7	4.6	-
		-	-	-	-	4.0	12^{3}	174.8	4.1	
**Ser-541**	Cα	5.7	2^{1}	17.6	4.9	-	-	-	-	-
**Ser-554**	Cβ	-	-	-	-	5.2	19	169.5	4.9	-
	Oγ	-	-	-	-	6.8	19	169.5	5.3	-
**Ser-63**	Oγ	-	-	-	-	6.9	2^{1}	17.4	3.9	-
**Thr-271**	Cβ	-	-	-	-	4.7	2	36.3	4.9	−0.1
		4.8	2^{1}	17.6	3.6	4.7	2^{1}	17.4	3.5	
	Oγ1	-	-	-	-	9.7	1	182.1	4.5	-

Oat PhyA3–PCB

^{1}H contact (residue)		Pr δ^H_{Pr} (ppm)	cofactor carbon	δ^C_{Pr} (ppm)	HM distance (Å)	Pfr δ^H_{Pfr} (ppm)	cofactor carbon	δ^C_{Pfr} (ppm)	HM distance (Å)	$\Delta\delta^H_{Pfr-Pr}$ (ppm)
**Tyr-241**	Cβ	-	-	-	-	3.2	17^1	9.9	4.3	**−0.6**
		3.8	18^1	16.7	4.3	3.2	18^1	16.0	3.2	
		3.8	18^2	12.9	2.8	-	-	-	-	
	Cδ1	10.4	18	133.1	5.6	-	-	-	-	-
		10.4	18^1	16.7	4.3	-	-	-	-	
		10.4	18^2	12.9	3.0	-	-	-	-	
	Cδ2	-	-	-	-	8.6	17	138.5	3.3	-
		-	-	-	-	8.6	17^1	9.9	2.8	
		-	-	-	-	8.6	18	140.7	3.6	
		-	-	-	-	8.6	18^1	16.0	3.4	
	Cε1	9.0	17^1	10.1	5.0	-	-	-	-	
		9.0	18^1	16.7	4.5	-	-	-	-	
		9.0	18^2	12.9	3.9	-	-	-	-	
	Cε2	-	-	-	-	8.8	13	129.3	5.0	-
		-	-	-	-	8.8	13^1	12.8	3.6	
		-	-	-	-	8.8	17^1	9.9	4.4	
**Tyr-268**	Cβ	-	-	-	-	3.7	16	150.3	4.1	-
		-	-	-	-	3.7	17	138.5	4.5	
		-	-	-	-	3.7	18	140.7	3.8	
	Cε1	9.6	17^1	10.1	5.1	-	-	-	-	-
		9.6	18	133.1	5.7	-	-	-	-	
		9.6	18^1	16.7	5.3	-	-	-	-	
	Cε2	9.7	18^1	16.7	4.3	10.5	18	140.7	4.8	**+0.8**
		9.7	18^2	12.9	4.7	-	-	-	-	
	Oη	8.0	18^1	16.7	5.1	-	-	-	-	-
		8.0	18^2	12.9	5.9	-	-	-	-	
**Tyr-326**	Cβ	4.0	1 [a]	182.4	4.5	-	-	-	-	-
	Cδ1	10.6	1 [b]	183.2	3.5	10.5	1	182.1	3.8	**−0.1**
	Cε1	11.8	1 [a]	182.4	4.2	11.7	1	182.1	3.5	**−0.1**
		11.8	17^1	10.1	5.0	-	-	-	-	
	Cε2					10.8	14	149.9	4.5	**−0.4**
		11.2	15	93.8	4.3	10.8	15	93.0	4.3	
		11.2	17	143.0	4.1	-	-	-	-	
	Oη	8.3	15	93.8	4.9	10.0	15	93.0	4.6	**+1.7**
		8.3	16	146.3	4.6	10.0	16	150.3	4.7	
		8.3	17	143.0	4.3	-	-	-	-	
		8.3	17^1	10.1	4.0	-	-	-	-	
**Tyr-69**	Cδ2	-	-	-	-	9.4	3^2	21.5	4.2	-
	Cε2	-	-	-	-	10.1	3	53.9	5.0	-
		-	-	-	-	10.1	3^1	49.0	4.1	
		-	-	-	-	10.1	3^2	21.5	2.8	
**Val-251**	Cγ2	1.1	18^1	16.7	4.2	-	-	-	-	-
		1.1	18^2	12.9	3.5	-	-	-	-	
**Val-337***	Cβ	-	-	-	-	2.3	12^2	38.7	4.0	-
		-	-	-	-	2.3	12^3	174.8	4.1	
	Cγ1	-	-	-	-	1.1	12	144.7	4.9	**+0.2**
		-	-	-	-	1.1	12^1	20.6	3.4	
		0.9	12^2	42.1	4.0	1.1	12^2	38.7	3.6	
		0.9	13^1	10.3	4.2	-	-	-	-	

*Continued on following page.

Oat PhyA3–PCB

^{1}H contact (residue)		Pr δ^{H}_{Pr} (ppm)	Pr cofactor carbon	Pr δ^{C}_{Pr} (ppm)	Pr HM distance (Å)	Pfr δ^{H}_{Pfr} (ppm)	Pfr cofactor carbon	Pfr δ^{C}_{Pfr} (ppm)	Pfr HM distance (Å)	$\Delta\delta^{H}_{Pfr-Pr}$ (ppm)
**Val-337**	Cγ2	1.3	12^{1}	22.8	4.1	-	-	-	-	-
		1.3	12^{2}	42.1	2.7	-	-	-	-	
		1.3	12^{3a}	176.7	3.4	-	-	-	-	
**Val-370**	Cβ	2.2	13^{1}	10.3	4.4	-	-	-	-	-
		2.2	19	172.4	5.0	-	-	-	-	
	Cγ1	-	-	-	-	1.1	12^{3}	174.8	3.9	**+0.2**
		-	-	-	-	1.1	13	129.3	4.2	
		0.9	13^{1}	10.3	4.8	-	-	-	-	
		0.9	19	172.4	2.8	-	-	-	-	
**W1**	W1-**I**	7.0	1	182.4/183.2	-	-	-	-	-	
		7.0	2	36.9	-	-	-	-	-	
		7.0	5^{a}	85.4	-	-	-	-	-	
		7.0	10	113.0	-	-	-	-	-	
	W1-**II** (pw)	7.9	1^{a}	182.4	3.6	7.8	1^{1}	182.1	3.6	**−0.1**
		7.9	4	153.1	3.8	7.8	4	152.7	3.7	
		7.9	5	85.4/86.1	4.2	-	-	-	-	
		-	-	-	-	7.8	6	155.7	3.8	
		-	-	-	-	7.8	9	131.3	3.9	
		7.9	10	113.0	4.2	-	-	-	-	
		7.9	11	127.4	3.9	-	-	-	-	
		7.9	15	93.8	3.7	-	-	-	-	
**W2**		8.0	12^{3b}	177.2	3.3	-	-	-	-	-
**W3**		8.1	12^{2}	42.1	4.4	-	-	-	-	-
		8.1	12^{3}	176.7/177.2	3.3	-	-	-	-	
**W4 (Pr-II)**		8.9	12^{2}	42.1	4.4	-	-	-	-	-
		8.9	12^{3b}	177.2	3.9	-	-	-	-	
**W5 (Pr-II)**		8.8	12^{3a}	176.7	-	-	-	-	-	-
		8.8	13	123.4	-	-	-	-	-	
		8.8	19	172.4	-	-	-	-	-	
**{W5+[OH]$^{-}_{W4}$} (Pr-I)**		9.6	12^{1}	22.8	-	-	-	-	-	-
		9.6	13	123.4	-	-	-	-	-	
		9.6	14	147.1	-	-	-	-	-	
		9.6	16	146.3	-	-	-	-	-	
		9.6	19	172.4	-	-	-	-	-	

Supplementary Table S7. Selective observation of interfacial 2.5–6.5 Å ^{1}H contacts of the non-quaternary PCB carbon atoms. The SIDY data were obtained from *u*-[^{13}C,^{15}N]-PCB-*As*.phyA3 with a ^{1}H spin-diffusion mixing time of 800 μs and a CP contact time of 96 μs as both Pr and Pfr. The original Pr data are from Song et al. (2012) and the reassigned Pr resonances listed here were taken from Supplementary Table S11. The $^{1}H^{residue}–^{13}C^{PCB}$ distances ("HM distance") of Pfr contacts were extracted from the corresponding Pfr model for the *As*.phyA3–PCB. ^{1}H NMR chemical shift differences ($\Delta\delta^{H}_{Pfr-Pr}$) of the chromophore contacts during Pr-to-Pfr photoconversion are listed at the right-most column as Pfr minus Pr.

Oat PhyA3–PCB

	Pr				Pfr				
cofactor carbon	δ^{C}_{Pr} (ppm)	^{1}H contact (residue)	δ^{H}_{Pr} (ppm)	HM distance (Å)	δ^{C}_{Pfr} (ppm)	^{1}H contact (residue)	δ^{H}_{Pfr} (ppm)	HM distance (Å)	$\Delta\delta^{H}_{Pfr-Pr}$ (ppm)
**2**	**36.9**	-	-	-	**36.3**	Arg-548 Cβ	3.3	5.8	-
		Arg-552 N	12.5	5.0		-	-	-	-
		Ile-67 Cγ1	3.6	5.1		-	-	-	-
		-	-	-		Ile-67 Cδ1	1.3	3.9	-
		-	-	-		Met-549 Cγ	4.3	5.3	-
		-	-	-		Met-549 Cε	2.9	4.1	-
		-	-	-		Pro-274 Cγ	3.7	4.7	-
		Pro-551 Cα	4.5	3.4		-	-	-	-
		Pro-551 Cβ	2.2	3.6		-	-	-	-
		Pro-551 Cδ	6.4	4.9		-	-	-	-
		-	-	-		Ser-63 Oγ	6.9	5.4	-
		Ser-541 Cα	5.7	4.9		-	-	-	-
		Thr-271 Cβ	4.8	5.0		-	-	-	-
		-	-	-		Thr-271 Oγ1	9.7	5.4	-
		-	-	-		Tyr-326 Cδ1	10.5	4.9	-
		Tyr-326 Cε1	11.8	5.5		-	-	-	-
		W1-**II**	7.9	5.0		-	-	-	-
**2[1]**	**17.6**	-	-	-	**17.4**	Arg-548 Cβ	3.3	5.4	-
		Arg-552 N	12.5	4.9		-	-	-	-
		-	-	-		Asp-272 Cα	5.3	3.6	-
		Asp-272 N	12.5	5.4		-	-	-	-
		Pro-551 Cδ	6.4	6.2		-	-	-	-
		-	-	-		Ser-63 Oγ	6.9	3.9	-
		Ser-541 Cα	5.7	4.4		-	-	-	-
		Ser-541 Cβ	3.2	4.9		-	-	-	-
		Thr-271 Cγ2	2.1	5.7		-	-	-	-
		Thr-271 Oγ1	9.4	5.9		Thr-271 Oγ1	9.7	4.4	+0.3
**3**	**53.9[a]/55.8[b]**	Asp-272 N[a]	12.5	6.1	**53.9**	-	-	-	-
		Cys-322 Cβ	4.5	3.8		-	-	-	-
		-	-	-		Ile-67 Cγ1	2.2	4.4	-
		-	-	-		Ile-67 Cγ2	2.2	4.1	-
		-	-	-		Ile-67 Cδ1	1.3	3.5	-
		-	-	-		Pro-274 Cγ	3.7	3.7	-
		-	-	-		Pro-274 Cδ	6.2	5.1	-
		Pro-551 Cα	4.5	4.6		-	-	-	-
		Pro-551 Cβ[a]	2.2	4.6		-	-	-	-
		-	-	-		Tyr-69 Cε2	10.1	5.0	-
**3[1]**	**47.7[a]/48.9[b]**	-	-	-	**49.0**	Cys-322 Cα	5.8	4.6	-
		Cys-322 Cβ[b]	4.5	3.0		Cys-322 Cβ	4.9	2.8	+0.4
		-	-	-		Cys-322 N	12.0	5.0	-
		His-323 N	11.7	5.6		His-323 N	11.4	5.3	−0.3
		His-550 Cα[a]	6.0	4.8		-	-	-	-
		His-550 N[a]	11.4	5.9		-	-	-	-
		-	-	-		Ile-67 Cγ1	2.2	4.4	-
		-	-	-		Ile-67 Cγ2	2.2	4.1	-
		-	-	-		Met-549 Cε	2.9	4.8	-
		-	-	-		Pro-274 Cγ	3.7	5.2	-
		Pro-551 Cα[b]	4.5	4.7		-	-	-	-
		-	-	-		Tyr-69 Cδ2	9.4	4.7	-

Oat PhyA3–PCB

	Pr				Pfr				
cofactor carbon	δ^{C}_{Pr} (ppm)	^{1}H contact (residue)	δ^{H}_{Pr} (ppm)	HM distance (Å)	δ^{C}_{Pfr} (ppm)	^{1}H contact (residue)	δ^{H}_{Pfr} (ppm)	HM distance (Å)	$\Delta\delta^{H}_{Pfr-Pr}$ (ppm)
**3^2**	**23.0[a]/21.6[b]**	Cys-322 $C\alpha$[b]	6.2	5.4	**21.5**	-	-	-	-
		Cys-322 $C\beta$[b]	4.5	3.8		Cys-322 $C\beta$	4.9	3.4	+0.4
		-	-	-		Cys-322 N	12.0	5.6	-
		His-550 $C\alpha$[b]	6.0	4.3		-	-	-	-
		His-550 N[b]	11.4	4.7		-	-	-	-
		-	-	-		Ile-67 $C\gamma1$	2.2	3.3	-
		-	-	-		Ile-67 $C\delta1$	1.3	4.1	-
		Met-549 $C\alpha$[b]	5.2	5.1		-	-	-	-
		Met-549 $C\beta$[b]	2.6	3.2		-	-	-	-
		Pro-551 $C\alpha$[b]	4.5	4.5		-	-	-	-
		Ser-541 $C\beta$[b]	3.2	5.3		-	-	-	-
		-	-	-		Tyr-69 $C\varepsilon2$	10.1	2.8	-
**5**	**85.4[a]/86.1[b]**	Asp-272 $C\alpha$[b]	5.2	5.4	**86.7**	-	-	-	-
		Cys-322 $C\alpha$[a]	6.2	5.8		-	-	-	-
		Cys-322 $C\beta$[a]	4.5	3.0		Cys-322 $C\beta$	4.9	3.7	+0.4
		Cys-322 N[b]	11.0	5.6		-	-	-	-
		His-323 $C\alpha$[a]	5.5	4.8		His-323 $C\alpha$	5.4	4.8	−0.1
		His-323 $C\beta$[a]	4.0	4.4		-	-	-	-
		His-323 N[b]	11.7	4.6		His-323 N	11.4	4.9	−0.3
		-	-	-		Ile-67 $C\gamma2$	2.2	4.8	-
		-	-	-		Ile-67 $C\delta1$	1.3	4.9	-
		Pro-274 $C\beta$	2.3	5.7		Pro-274 $C\beta$	2.9	5.5	+0.6
		-	-	-		Pro-274 $C\gamma$	3.7	3.2	-
		-	-	-		Pro-274 $C\delta$	6.2	3.1	-
		W1-**I**[a]	7.0	-		-	-	-	-
**7^1**	**7.3**	-	-	-	**7.3**	Ala-277 $C\beta$	2.3	3.9	-
		-	-	-		Cys-322 $C\beta$	4.9	4.5	-
		Cys-322 N	11.0	4.9		-	-	-	-
		-	-	-		His-320 $C\alpha$	5.0	4.8	-
		His-320 $C\beta$	3.2	2.8		-	-	-	-
		His-320 N	12.0	4.8		His-320 N	12.4	4.7	+0.4
		His-320 $N\varepsilon2$	13.2	5.9		-	-	-	-
		-	-	-		His-323 $C\beta$	4.3	3.9	-
		Pro-274 $C\beta$	2.3	5.2		Pro-274 $C\beta$	2.9	4.7	+0.6
		Pro-274 $C\gamma$	4.8	3.1		-	-	-	-
		-	-	-		Pro-274 $C\delta$	6.2	3.8	-
**8^1**	**22.0**	-	-	-	**22.9**	Arg-287 $N\varepsilon$	10.6	4.2	-
		-	-	-		Arg-287 $N\eta2$	9.9	4.9	-
		-	-	-		Arg-317 $N\eta1$	11.7	5.4	-
		Arg-317 $N\eta2$	7.2	4.9		Arg-317 $N\eta2$	8.5	5.7	+1.3
		His-320 $C\beta$	3.2	3.4		-	-	-	-
		His-320 N	12.0	3.1		-	-	-	-
		-	-	-		His-323 $C\beta$	4.3	3.8	-
		-	-	-		His-323 $C\varepsilon1$	12.3	6.1	-
		His-323 $C\delta2$-**II**	12.8	4.9		-	-	-	-
		His-323 $N\delta1$-**I**	16.9	-		-	-	-	-
		-	-	-		Phe-281 $C\varepsilon1$	7.7	4.8	-
		-	-	-		Phe-281 $C\zeta$	7.1	3.9	-
		Pro-274 $C\gamma$	4.8	4.2		-	-	-	-
		-	-	-		Pro-319 $C\beta$	2.9	4.7	-

Oat PhyA3–PCB

	Pr				Pfr				
cofactor carbon	δ^{C}_{Pr} (ppm)	^{1}H contact (residue)	δ^{H}_{Pr} (ppm)	HM distance (Å)	δ^{C}_{Pfr} (ppm)	^{1}H contact (residue)	δ^{H}_{Pfr} (ppm)	HM distance (Å)	$\Delta\delta^{H}_{Pfr-Pr}$ (ppm)
**8^2**	**41.4**	-	-	-	**41.2**	Ala-277 Cβ	2.3	4.5	-
		-	-	-		Arg-287 Nε	10.6	3.9	-
		-	-	-		Arg-287 Nη2	9.9	3.9	-
		Arg-317 Nη1	10.0	3.9		Arg-317 Nη1	11.7	4.0	+1.7
		Arg-317 Nη2	7.2	3.9		Arg-317 Nη2	8.5	4.7	+1.3
		-	-	-		His-320 Cα	5.0	5.3	-
		His-320 N	12.0	3.9		His-320N	12.4	3.8	+0.4
		-	-	-		Phe-281 Cε1	7.7	5.1	-
		Pro-274 Cγ	4.8	4.1		-	-	-	-
		-	-	-		Pro-319 Cα	5.8	4.2	-
		Pro-319 Cβ	3.2	4.6		-	-	-	-
**10**	**113.0**	Ala-277 Cβ	2.0	4.4	**112.0**	-	-	-	-
		His-323 Cα	5.5	4.7		His-323 Cα	5.4	5.4	−0.1
		His-323 Cβ	4.0	3.1		His-323 Cβ	4.3	4.3	+0.3
		His-323 Cδ2-**I**	16.1	-		-	-	-	-
		His-323 Cε1-**II**	12.1	4.0		His-323 Cε1	12.3	4.3	+0.2
		Ile-273 Cα	4.3	4.4		Ile-273 Cα	4.8	4.5	+0.5
		-	-	-		Ile-273 Cδ1	2.0	3.2	-
		-	-	-		Phe-281 Cζ	7.1	4.5	-
		-	-	-		Pro-274 Cδ	6.2	4.8	-
		Tyr-326 Cδ2	11.0	5.2		-	-	-	-
		W1-**I**	7.0	-		-	-	-	-
		W1-**II**	7.9	4.2		-	-	-	-
**12^1**	**22.8**	Arg-287 Cβ	2.8	5.7	**20.6**	-	-	-	-
		His-323 Cδ2-**II**	12.8	5.0		-	-	-	-
		His-323 Nε2-**II**	14.7	4.1		His-323 Nε2	13.5	5.3	−1.2
		-	-	-		Leu-368 Cδ1	1.8	4.1	-
		Phe-281 Cζ	7.2	3.1		Phe-281 Cζ	7.1	5.1	−0.1
		Val-337 Cβ	2.2	5.5		-	-	-	-
		-	-	-		Val-337 Cγ1	1.1	3.4	-
		Val-337 Cγ2	1.3	4.1		-	-	-	-
		{W5+$[OH]^{-}_{W4}$}(**Pr-I**)	9.6	-		-	-	-	-
**12^2**	**42.1**	Arg-287 Cδ	4.6	4.7	**38.7**	-	-	-	-
		-	-	-		His-323 Cε1	12.3	3.0	-
		His-323 Nδ1-**I**	16.9	-		-	-	-	-
		His-323 Nε2-**I**	18.4	-		-	-	-	-
		-	-	-		His-323 Nε2	13.5	4.0	-
		-	-	-		His-372 Cδ2	11.5	5.5	-
		-	-	-		Ile-289 Cδ	1.9	4.4	-
		-	-	-		Met-330 Cε	3.3	5.7	-
		-	-	-		Ser-335 Cβ	4.0	4.6	-
		Val-337 Cβ	2.2	4.2		-	-	-	-
		Val-337 Cγ2	1.3	2.7		-	-	-	-
		-	-	-		Val-370 Cγ1	1.1	4.4	-
		W4 (**Pr-II**)	8.9	4.4		-	-	-	-
**13^1**	**10.3**	His-323 Cε1-**II**	12.1	4.0	**12.8**	His-323 Cε1	12.3	4.9	+0.2
		His-323 Nε2-**I**	18.4	-		-	-	-	-
		-	-	-		His-372 Cε1	9.9	6.4	-
		Ile-273 Cα	4.3	5.3		-	-	-	-
		-	-	-		Leu-368 Cβ	2.9	4.5	-
		Leu-368 Cγ	2.3	5.4		-	-	-	-
		Leu-368 Cδ1	1.7	3.2		Leu-368 Cδ1	1.8	4.7	+0.1
		-	-	-		Tyr-241 Cδ2	8.6	4.4	-
		Val-370 Cγ1	0.9	4.8		Val-370 Cγ1	1.1	3.0	+0.2

Oat PhyA3–PCB

	Pr				Pfr				
cofactor carbon	δ^{C}_{Pr} (ppm)	¹H contact (residue)	δ^{H}_{Pr} (ppm)	HM distance (Å)	δ^{C}_{Pfr} (ppm)	¹H contact (residue)	δ^{H}_{Pfr} (ppm)	HM distance (Å)	$\Delta\delta^{H}_{Pfr-Pr}$ (ppm)
**15**	**94.8**	Asp-272 $C\beta$	3.5	3.6	**93.0**	Asp-272 $C\beta$	2.9	3.8	**−0.6**
		His-323 $C\delta 2$-**I**	16.1	-		-	-	-	-
		His-323 $N\delta 1$-**I**	16.9	-		-	-	-	-
		His-323 $N\varepsilon 2$-**II**	14.7	5.7		-	-	-	-
		-	-	-		Ile-273 $C\alpha$	4.8	4.7	-
		Ile-273 $C\beta$	2.9	5.5		-	-	-	-
		-	-	-		Ile-273 $C\gamma 1$	1.4	2.8	-
		-	-	-		Ile-273 $C\delta 1$	2.0	3.5	-
		-	-	-		Tyr-241 $C\varepsilon 2$	8.8	5.3	-
		Tyr-326 $C\varepsilon 1$	11.8	5.8		-	-	-	-
		-	-	-		Tyr-326 $C\varepsilon 2$	10.8	4.3	-
		-	-	-		Tyr-326 $O\eta$	10.0	4.6	-
		W1-**II**	7.9	3.7		-	-	-	-
		W5 (**Pr-II**)	8.8	-		-	-	-	-
**17¹**	**10.1**	Arg-552 $N\eta 2$	12.4	5.8	**9.9**	-	-	-	-
		-	-	-		Ile-273 $C\gamma 1$	1.4	3.7	-
		Ile-273 $C\delta 1$	1.8	3.0		Ile-273 $C\delta 1$	2.0	3.5	**+0.2**
		-	-	-		Leu-368 $C\beta$	2.9	3.3	-
		-	-	-		Leu-368 $C\delta 1$	1.8	2.8	-
		-	-	-		Phe-243 $C\varepsilon 1$	7.9	5.3	-
		-	-	-		Phe-243 $C\zeta$	7.0	4.2	-
		-	-	-		Tyr-241 $C\beta$	3.2	4.3	-
		-	-	-		Tyr-241 $C\delta 2$	8.6	2.8	-
		Tyr-241 $C\varepsilon 1$	9.0	5.0		-	-	-	-
		-	-	-		Tyr-241 $C\varepsilon 2$	8.8	4.4	-
		Tyr-268 $C\beta$	3.8	3.4		-	-	-	-
		Tyr-268 $C\delta 1$	11.4	4.5		-	-	-	-
		Tyr-326 $O\eta$	8.3	4.0		-	-	-	-
**18¹**	**16.7**	-	-	-	**16.0**	Glu-250 $C\alpha$	5.5	5.7	-
		-	-	-		Glu-250 N	10.8	4.8	-
		-	-	-		Gly-249 $C\alpha$	4.5	3.4	-
		-	-	-		Gly-249 N	11.7	5.3	-
		His-372 $N\varepsilon 2$ (**Pr-II**)	12.2	5.3		-	-	-	-
		-	-	-		Leu-368 $C\beta$	2.9	4.3	-
		-	-	-		Lys-242 N	12.3	4.7	-
		Met-239 $C\varepsilon$	3.1	3.8		-	-	-	-
		-	-	-		Phe-243 $C\delta 1$	8.5	4.8	-
		-	-	-		Phe-243 $C\zeta$	7.0	4.0	-
		Tyr-241 $C\varepsilon 1$	9.0	4.5		-	-	-	-
		Tyr-268 $O\eta$	8.0	5.1		-	-	-	-
		Tyr-326 $O\eta$	8.3	5.4		-	-	-	-
**18²**	**12.9**	-	-	-	**13.3**	Glu-250 N	10.8	3.9	-
		-	-	-		Gly-249 $C\alpha$	4.5	2.8	-
		-	-	-		Gly-249 N	11.7	4.7	-
		-	-	-		Lys-242 N	12.3	4.1	-
		Met-239 $C\varepsilon$	3.1	3.5		-	-	-	-
		-	-	-		Phe-243 $C\varepsilon 1$	7.9	4.7	-
		-	-	-		Ser-554 $C\beta$	5.2	6.1	
		-	-	-		Tyr-241 $C\beta$	3.2	2.7	-
		Tyr-241 $C\delta 1$	10.4	3.0		-	-	-	-
		Tyr-241 $C\varepsilon 1$	9.0	3.9		-	-	-	-
		Tyr-268 $C\beta$	3.8	4.7		-	-	-	-
		Tyr-268 $C\delta 1$	11.4	6.3		-	-	-	-
		Tyr-268 $O\eta$	8.0	5.9		-	-	-	-
		Val-251 $C\alpha$	4.5	5.8		-	-	-	-

Supplementary Table S8. The interfacial ^{13}C chromophore contacts of the protein residues. This table corresponds to the interfacial $^{1}H^{residue}$–$^{13}C^{PCB}$ contacts summarized in Supplementary Table S7. ^{1}H NMR chemical shift differences ($\Delta\delta^{H}_{Pfr-Pr}$) of the observed protein residues during the Pr-to-Pfr photoconversion are reported as Pfr minus Pr and listed at the right-most column. The nearest chromophore carbon contacts of a proton attached to the protein residue ($^{1}H^{residue}$) are labeled in green (see also Supplementary Table S9).

Oat PhyA3–PCB

		Pr				Pfr				
^{1}H contact (residue)		δ^{H}_{Pr} (ppm)	cofactor carbon	δ^{C}_{Pr} (ppm)	HM distance (Å)	δ^{H}_{Pfr} (ppm)	cofactor carbon	δ^{C}_{Pfr} (ppm)	HM distance (Å)	$\Delta\delta^{H}_{Pfr-Pr}$ (ppm)
**Ala-277**	Cβ	-	-	-	-	2.3	7^1	7.3	3.9	**+0.3**
		-	-	-	-	2.3	8^2	41.2	4.5	
		2.0	10	113.0	4.4	-	-	-	-	
**Arg-287**	Cβ	2.8	12^1	22.8	5.7	-	-	-	-	-
	Cδ	4.6	12^2	42.1	4.7	-	-	-	-	-
	Nε	-	-	-	-	10.6	8^1	22.9	4.2	-
		-	-	-	-	10.6	8^2	41.2	3.9	
	Nη2	-	-	-	-	9.9	8^1	22.9	4.9	-
		-	-	-	-	9.9	8^2	41.2	3.9	
**Arg-317**	Nη1	10.0	8^2	41.4	3.9	11.7	8^1	22.9	5.4	**+1.7**
		-	-	-	-	11.7	8^2	41.2	4.0	
	Nη2	7.2	8^1	22.0	4.9	8.5	8^1	22.9	5.7	**+1.3**
		7.2	8^2	41.4	3.9	8.5	8^2	41.2	4.7	
**Arg-548**	Cβ	-	-	-	-	3.3	2	36.3	5.8	-
		-	-	-	-	3.3	2^1	17.4	5.4	
**Arg-552**	N	12.5	2	36.9	5.0	-	-	-	-	-
		12.5	2^1	17.6	4.9	-	-	-	-	
	Nη2	12.4	17^1	10.1	5.8	-	-	-	-	-
**Asp-272**	Cα	-	-	-	-	5.3	2^1	17.4	3.6	**+0.1**
		5.2	5^{b}	86.1	5.4	-	-	-	-	
	Cβ	3.5	15	93.8	3.6	2.9	15	93.0	3.8	**−0.6**
	N	12.5	2^1	17.6	5.4	-	-	-	-	-
		12.5	3^{a}	53.9	6.1	-	-	-	-	
**Cys-322**	Cα	-	-	-	-	5.8	3^1	49.0	4.6	**−0.4**
		6.2	3^{2b}	21.6	5.4	-	-	-	-	
		6.2	5^{a}	85.4	5.8	-	-	-	-	
	Cβ	4.5	3	53.9/55.8	3.8	-	-	-	-	**+0.4**
		4.5	3^{1b}	48.9	3.0	4.9	3^1	49.0	2.8	
		4.5	3^{2b}	21.6	3.8	4.9	3^2	21.5	3.4	
		4.5	5^{a}	85.4	3.0	4.9	5	86.7	3.7	
		-	-	-	-	4.9	7^1	7.3	4.5	
	N	-	-	-	-	12.0	3^1	49.0	5.0	**+1.0**
		-	-	-	-	12.0	3^2	21.5	5.6	
		11.0	5^{b}	86.1	5.6	-	-	-	-	
		11.0	7^1	7.3	4.9	-	-	-	-	
**Glu-250**	Cα	-	-	-	-	5.5	18^1	16.0	5.7	-
	N	-	-	-	-	10.8	18^1	16.0	4.8	-
		-	-	-	-	10.8	18^2	13.3	3.9	
**Gly-249**	Cα	-	-	-	-	4.5	18^1	16.0	3.4	-
		-	-	-	-	4.5	18^2	13.3	2.8	
	N	-	-	-	-	11.5	18^1	16.0	5.3	-
		-	-	-	-	11.5	18^2	13.3	4.7	
**His-320***	Cα	-	-	-	-	5.0	7^1	7.3	4.8	-
		-	-	-	-	5.0	8^2	41.2	5.3	
	Cβ	3.2	7^1	7.3	2.8	-	-	-	-	-
		3.2	8^1	22.0	3.4	-	-	-	-	

*Continued on following page.

Oat PhyA3–PCB

^{1}H contact (residue)		Pr				Pfr				
		δ^H_{Pr} (ppm)	cofactor carbon	δ^C_{Pr} (ppm)	HM distance (Å)	δ^H_{Pfr} (ppm)	cofactor carbon	δ^C_{Pfr} (ppm)	HM distance (Å)	$\Delta\delta^H_{Pfr-Pr}$ (ppm)
**His-320**	N	12.0	7^1	7.3	4.8	12.4	7^1	7.3	4.7	+0.4
		12.0	8^1	22.0	3.1	-	-	-	-	
		12.0	8^2	41.4	3.9	12.4	8^2	41.2	3.8	
	Nε2	13.2	7^1	7.3	5.9	-	-	-	-	-
**His-323**	Cα	5.5	5 [a]	85.4	4.8	5.4	5	86.7	4.8	−0.1
		5.5	10	113.0	4.7	5.4	10	112.0	5.4	
	Cβ	4.0	5 [a]	85.4	4.4	-	-	-	-	+0.3
		-	-	-	-	4.3	7^1	7.3	3.9	
		-	-	-	-	4.3	8^1	22.9	3.8	
		4.0	10	113.0	3.1	4.3	10	112.0	4.3	
	Cδ2-**I**	16.1	10	113.0	-	-	-	-	-	-
		16.1	15	93.8	-	-	-	-	-	
	Cδ2-**II**	12.8	8^1	22.0	4.9	-	-	-	-	-
		12.8	12^1	22.8	5.0	-	-	-	-	
	Cε1-**II**	-	-	-	-	12.3	8^1	22.9	6.1	+0.2
		12.1	10	113.0	4.0	12.3	10	112.0	4.3	
		-	-	-	-	12.3	12^2	38.7	3.0	
		12.1	13^1	10.3	4.0	12.3	13^1	12.8	4.9	
	N	11.7	3^1	47.7/48.9	5.6	11.4	3^1	49.0	5.3	−0.3
		11.7	5 [b]	86.1	4.6	11.4	5	86.7	4.9	
	Nδ1-**I**	16.9	8^1	22.0	-	-	-	-	-	-
		16.9	12^2	42.1	-	-	-	-	-	
		16.9	15	93.8	-	-	-	-	-	
	Nε2-**I**	18.4	12^2	42.1	-	-	-	-	-	-
		18.4	13^1	10.3	-	-	-	-	-	
	Nε2-**II**	14.7	12^1	22.8	4.1	13.5	12^1	20.6	5.3	−1.2
		-	-	-	-	13.5	12^2	38.7	4.0	
		14.7	15	93.8	5.7	-	-	-	-	
**His-372**	Cδ2	-	-	-	-	11.5	12^2	38.7	5.5	-
	Cε1-**II**	-	-	-	-	9.9	13^1	12.8	5.1	-
**His-550**	Cα	6.0	3^1[a]	47.7	4.8	-	-	-	-	-
		6.0	3^2[b]	21.6	4.3	-	-	-	-	
	N	11.4	3^1[a]	47.7	5.9	-	-	-	-	-
		11.4	3^2[b]	21.6	4.7	-	-	-	-	
**Ile-273**	Cα	4.3	10	113.0	4.4	4.8	10^1	112.0	4.5	+0.5
		4.3	13^1	10.3	5.3	-	-	-	-	
		-	-	-	-	4.8	15	93.0	4.7	
	Cβ	2.9	15	93.8	5.5	-	-	-	-	-
	Cγ1	-	-	-	-	1.4	15	93.0	2.8	-
		-	-	-	-	1.4	17^1	9.9	3.7	
	Cδ1	-	-	-	-	2.0	10	112.0	3.2	+0.2
		-	-	-	-	2.0	15	93.0	3.5	
		1.8	17^1	10.1	3.0	2.0	17^1	9.9	3.5	
**Ile-289**	Cδ1	-	-	-	-	1.9	12^2	38.7	4.4	-
**Ile-67***	Cγ1	3.6	2	36.9	5.1	-	-	-	-	−1.4
		-	-	-	-	2.2	3	53.9	4.4	
		-	-	-	-	2.2	3^1	49.0	4.4	
		-	-	-	-	2.2	3^2	21.5	3.3	

*Continued on following page.

Oat PhyA3–PCB

^{1}H contact (residue)		Pr δ^{H}_{Pr} (ppm)	Pr cofactor carbon	Pr δ^{C}_{Pr} (ppm)	Pr HM distance (Å)	Pfr δ^{H}_{Pfr} (ppm)	Pfr cofactor carbon	Pfr δ^{C}_{Pfr} (ppm)	Pfr HM distance (Å)	$\Delta\delta^{H}_{Pfr-Pr}$ (ppm)
**Ile-67**	Cγ2	-	-	-	-	2.2	3	53.9	4.1	-
		-	-	-	-	2.2	3^1	49.0	4.1	
		-	-	-	-	2.2	5	86.7	4.8	
	Cδ1	-	-	-	-	1.3	2	36.3	3.9	-
		-	-	-	-	1.3	3	53.9	3.5	
		-	-	-	-	1.3	3^2	21.5	4.1	
		-	-	-	-	1.3	5	86.7	4.9	
**Leu-368**	Cβ	-	-	-	-	2.9	13^1	12.8	4.5	-
		-	-	-	-	2.9	17^1	9.9	3.3	
		-	-	-	-	2.9	18^1	16.0	4.3	
	Cγ	2.3	13^1	10.3	5.4	-	-	-	-	-
	Cδ1	-	-	-	-	1.8	12^1	20.6	4.1	**+0.1**
		1.7	13^1	10.3	3.2	1.8	13^1	12.8	4.7	
		-	-	-	-	1.8	17^1	9.9	2.8	
**Lys-242**	N	-	-	-	-	12.3	18^1	16.0	4.7	-
		-	-	-	-	12.3	18^2	13.3	4.1	
**Met-239**	Cε	3.1	18^1	16.7	3.8	-	-	-	-	-
		3.1	18^2	12.9	3.5	-	-	-	-	
**Met-330**	Cε	-	-	-	-	3.3	12^2	38.7	5.7	-
**Met-549**	Cα	5.2	3^{2b}	21.6	5.1	-	-	-	-	-
	Cβ	2.6	3^{2b}	21.6	3.2	-	-	-	-	-
	Cγ	-	-	-	-	4.3	2	36.3	5.3	-
	Cε	-	-	-	-	2.9	2	36.3	4.1	-
		-	-	-	-	2.9	3^1	49.0	4.8	
**Phe-243**	Cδ1	-	-	-	-	8.5	18^1	16.0	4.8	-
	Cε1	-	-	-	-	7.9	17^1	9.9	5.3	-
		-	-	-	-	7.9	18^2	13.3	4.7	
	Cζ	-	-	-	-	7.0	17^1	9.9	4.2	-
		-	-	-	-	7.0	18^1	16.0	4.0	
**Phe-281**	Cε1	-	-	-	-	7.7	8^1	22.9	4.8	-
		-	-	-	-	7.7	8^2	41.2	5.1	
	Cζ	-	-	-	-	7.1	8^1	22.9	3.9	**−0.1**
		-	-	-	-	7.1	10	112.0	4.5	
		7.2	12^1	22.8	3.1	7.1	12^1	20.6	5.1	
**Pro-274**	Cβ	2.3	5	85.4/86.1	5.7	2.9	5	86.7	5.5	**+0.6**
		2.3	7^1	7.3	5.2	2.9	7^1	7.3	4.7	
	Cγ	-	-	-	-	3.7	2	36.3	4.7	**−1.1**
		-	-	-	-	3.7	3	53.9	3.7	
		-	-	-	-	3.7	3^1	49.0	5.2	
		-	-	-	-	3.7	5	86.7	3.2	
		4.8	7^1	7.3	3.1	-	-	-	-	
		4.8	8^1	22.0	4.2	-	-	-	-	
		4.8	8^2	41.4	4.1	-	-	-	-	
	Cδ	-	-	-	-	6.2	3	53.9	5.1	-
		-	-	-	-	6.2	5	86.7	3.1	
		-	-	-	-	6.2	7^1	7.3	3.8	
		-	-	-	-	6.2	10	112.0	4.8	
**Pro-319**	Cα	-	-	-	-	5.8	8^2	41.2	4.2	-
	Cβ	3.2	8^2	41.4	4.6	2.9	8^1	22.9	4.7	**−0.3**

		**Oat PhyA3–PCB**								
		**Pr**				**Pfr**				
^{1}H contact (residue)		δ^{H}_{Pr} (ppm)	cofactor carbon	δ^{C}_{Pr} (ppm)	HM distance (Å)	δ^{H}_{Pfr} (ppm)	cofactor carbon	δ^{C}_{Pfr} (ppm)	HM distance (Å)	$\Delta\delta^{H}_{Pfr-Pr}$ (ppm)
**Pro-551**	Cα	4.5	2	36.9	3.4	-	-	-	-	-
		4.5	3	53.9/55.8	4.6	-	-	-	-	
		4.5	3^{1b}	48.9	4.7	-	-	-	-	
		4.5	3^{2b}	21.6	4.5	-	-	-	-	
	Cβ	2.2	2	36.9	3.6	-	-	-	-	-
		2.2	3^{a}	53.9	4.6	-	-	-	-	
	Cδ	6.4	2	36.9	4.9	-	-	-	-	-
		6.4	2^{1}	17.6	6.2	-	-	-	-	
**Ser-335**	Cβ	-	-	-	-	4.0	12^{2}	38.7	4.6	-
**Ser-541**	Cα	5.7	2	36.9	4.9	-	-	-	-	-
		5.7	2^{1}	17.6	4.4	-	-	-	-	
	Cβ	3.2	2^{1}	17.6	4.9	-	-	-	-	-
		3.2	3^{2b}	21.6	5.3	-	-	-	-	
**Ser-554**	Cβ	-	-	-	-	5.2	18^{2}	13.3	6.1	-
**Ser-63**	Oγ	-	-	-	-	6.9	2	36.3	5.4	-
		-	-	-	-	6.9	2^{1}	17.4	3.9	
**Thr-271**	Cβ	4.8	2	36.9	5.0	-	-	-	-	-
	Cγ2	2.1	2^{1}	17.6	5.7	-	-	-	-	-
	Oγ1	-	-	-	-	9.7	2	36.3	5.4	**+0.3**
		9.4	2^{1}	17.6	5.9	9.7	2^{1}	17.4	4.4	
**Tyr-241**	Cβ	-	-	-	-	3.2	17^{1}	9.9	4.3	-
		-	-	-	-	3.2	18^{2}	13.3	2.7	
	Cδ1	10.4	18^{2}	12.9	3.0	-	-	-	-	-
	Cδ2	-	-	-	-	8.6	13^{1}	12.8	4.4	-
		-	-	-	-	8.6	17^{1}	9.9	2.8	
	Cε1	9.0	17^{1}	10.1	5.0	-	-	-	-	-
		9.0	18^{1}	16.7	4.5	-	-	-	-	
		9.0	18^{2}	12.9	3.9	-	-	-	-	
	Cε2	-	-	-	-	8.8	15	93.0	5.3	-
		-	-	-	-	8.8	17^{1}	9.9	4.4	
**Tyr-268**	Cβ	3.8	17^{1}	10.1	3.4	-	-	-	-	-
		3.8	18^{2}	12.9	4.7	-	-	-	-	
	Cδ1	11.4	17^{1}	10.1	4.5	-	-	-	-	-
		11.4	18^{2}	12.9	6.3	-	-	-	-	
	Oη	8.0	18^{1}	16.7	5.1	-	-	-	-	-
		8.0	18^{2}	12.9	5.9	-	-	-	-	
**Tyr-326**	Cδ1	-	-	-	-	10.5	2	36.3	4.9	-
	Cδ2	11.0	10	113.0	5.2	-	-	-	-	-
	Cε1	11.8	2	36.9	5.5	-	-	-	-	-
		11.8	15	93.8	5.8	-	-	-	-	
	Cε2	-	-	-	-	10.0	15	93.0	4.6	-
	Oη	-	-	-	-	10.0	15	93.0	4.6	**+1.7**
		8.3	17^{1}	10.1	4.0	-	-	-	-	
		8.3	18^{1}	16.7	5.4	-	-	-	-	
**Tyr-69**	Cδ2	-	-	-	-	9.4	3^{1}	49.0	4.7	-
	Cε2	-	-	-	-	10.1	3	53.9	5.0	-
		-	-	-	-	10.1	3^{2}	21.5	2.8	
**Val-251**	Cα	4.5	18^{2}	12.9	5.8	-	-	-	-	-
**Val-337***	Cβ	2.2	12^{1}	22.8	5.5	-	-	-	-	-
		2.2	12^{2}	42.1	4.2	-	-	-	-	

*Continued on following page.

Oat PhyA3–PCB

^{1}H contact (residue)		Pr: δ^H_{Pr} (ppm)	Pr: cofactor carbon	Pr: δ^C_{Pr} (ppm)	Pr: HM distance (Å)	Pfr: δ^H_{Pfr} (ppm)	Pfr: cofactor carbon	Pfr: δ^C_{Pfr} (ppm)	Pfr: HM distance (Å)	$\Delta\delta^H_{Pfr-Pr}$ (ppm)
**Val-337**	Cγ1	-	-	-	-	1.1	12^1	20.6	3.4	-
	Cγ2	1.3	12^1	22.8	4.1	-	-	-	-	-
		1.3	12^2	42.1	2.7	-	-	-	-	
**Val-370**	Cγ1	-	-	-	-	1.1	12^2	38.7	4.4	**+0.2**
		0.9	13^1	10.3	4.8	1.1	13^1	12.8	3.0	
**W1**	W1-**I**	7.0	5 [a]	85.4	-	-	-	-	-	-
		7.0	10	113.0	-	-	-	-	-	
	W1-**II** (pw)	7.9	2	36.9	5.0	-	-	-	-	-
		7.9	10	113.0	4.2	-	-	-	-	
		7.9	15	93.8	3.7	-	-	-	-	
**W4 (Pr-II)**		8.9	12^2	42.1	4.4	-	-	-	-	-
**W5 (Pr-II)**		8.8	15	93.8	-	-	-	-	-	-
**{W5+[OH]$^-_{W4}$} (Pr-I)**		9.6	12^1	22.8	-	-	-	-	-	-

Supplementary Table S9. The intermolecular ^{13}C chromophore contacts of the protein residues. The table corresponds to the MELODI–HETCOR and SIDY correlations (denoted by "M" and "S", respectively) summarized in Supplementary Tables S6 and S8. d_{min} represents the shortest intermolecular $^1H^{residue}$–$^{13}C^{PCB}$ distance in the structural models for characterization of protein residues (the relevant carbon atoms labeled in green). $\Delta\delta^{H}_{Pfr-Pr}$ of the observed protein residues during Pr-to-Pfr photoconversion are reported as Pfr minus Pr and illustrated in Figure 5. The nearest chromophore carbon contacts of a proton attached to the protein residues ($^1H^{residue}$) are labeled in green.

Oat PhyA3–PCB

		Pr			Pfr			
^{1}H contact (residue)		δ^{H}_{Pr} (ppm)	cofactor carbon (M / S)	HM distance (d_{min}, Å)	δ^{H}_{Pfr} (ppm)	cofactor carbon (M / S)	HM distance (d_{min}, Å)	$\Delta\delta^{H}_{Pfr-Pr}$ (ppm)
**Ala-277**	Cα	3.8	8^2, 8^3 / -	4.5	-	- / -	-	-
	Cβ	2.0	8, 8^3, 9, 11, 12^1 / 10	3.8	2.3	8^1, 8^2 / 7^1, 8^2	3.8	+0.3
**Arg-287**	Cβ	2.8	12^2, 12^{3a} / 12^1	4.1	-	- / -	-	-
	Cδ	4.6	- / 12^2	4.7	5.0	8^3 / -	4.6	+0.4
	Nε	-	- / -	-	10.6	8^1, 8^2, 8^3 / 8^1, 8^2	2.6	-
	Nη1	9.2	8^3 / -	-	-	- / -	-	-
	Nη2	-	- / -	-	9.9	8^1, 8^2, 8^3 / 8^1, 8^2	2.4	-
**Arg-317**	Nη1	10.0	8^1, 8^3 / 8^2	2.4	11.7	8^2, 8^3 / 8^1, 8^2	3.1	+1.7
	Nη2	7.2	8^3 / 8^1, 8^2	2.5	8.5	8^3 / 8^1, 8^2	4.5	+1.3
**Arg-548**	Cβ	-	- / -	-	3.3	- / 2, 2^1	5.4	-
**Arg-552**	N	12.5	- / 2, 2^1	4.9	-	- / -	-	-
	Nη2	12.4	- / 17^1	5.8	-	- / -	-	-
**Asp-272**	Cα	5.2	1^a, 2, 4 / 5^b	3.3	5.3	1, 2, 4 / 2^1	3.0	+0.1
	Cβ	3.5	15, 17^1 / 15	2.9	2.9	14, 15, 16, 19 / 15	3.8	−0.6
	N	12.5	1^a, 2^1 / 2^1, 3^a	5.4	-	- / -	-	-
**Cys-322**	Cα	6.2	3^{1b}, 3^{2b} / 3^{2b}, 5^a	4.7	5.8	3^1 / 3^1	4.6	−0.4
	Cβ	4.5	3, 3^1, 3^{2b}, 7^1 / 3, 3^{1b}, 3^{2b}, 5^a	3.0	4.9	3^1, 3^2, 4, 5 / 3^1, 3^2, 5, 7^1	2.8	+0.4
	N	-	- / -	-	12.0	- / 3^1, 3^2	5.0	-
**Glu-250**	Cα	-	- / -	-	5.5	- / 18^1	5.7	-
	N	-	- / -	-	10.8	18^2 / 18^1, 18^2	3.9	-
**Gly-249**	Cα	-	- / -	-	4.5	18, 18^1, 18^2 / 18^1, 18^2	2.8	-
	N	-	- / -	-	11.7	18, 18^2 / 18^1, 18^2	4.7	-
**His-320**	Cα	-	- / -	-	5.0	7^1 / 7^1, 8^2	4.8	-
	Cβ	3.2	7, 7^1, 8^1 / 7^1, 8^1	2.8	-	- / -	-	-
	N	12.0	8^1, 8^3 / 7^1, 8^1, 8^2	3.1	12.4	7^1, 8^2, 8^3 / 7^1, 8^2	3.8	+0.4
	Nε2	13.2	- / 7^1	5.9	-	- / -	-	-
**His-323**	Cα	5.5	6, 7, 9 / 5^a, 10	4.3	5.4	6, 8, 9 / 5, 10	4.5	−0.1
	Cβ	4.0	7, 8, 9, 10 / 5^a, 10	2.5	4.3	7, 7^1, 8, 9 / 7^1, 8^1, 10	3.2	+0.3
	Cδ2-**I**	16.1	10, 11, 12, 13 / 10, 15	-	-	- / -	-	-
	Cδ2-**II**	12.8	8^1, 11, 12 / 8^1, 12^1	4.9	-	- / -	-	-
	Cε1-**I**	13.6	9, 10, 12, 12^{3b}, 15 / -	-	-	- / -	-	-
	Cε1-**II**	12.1	10, 11, 12, 12^2, 14, 15 / 10, 13^1	3.0	12.3	8, 10, 11, 12^1 / 8^1, 10, 12^2, 13^1	3.0	+0.2
	N	11.7	6, 7^1, 9 / 3^1, 5^b	4.2	-	7^1, 9 / 3^1, 5	4.9	−0.3
	Nδ1-**I**	16.9	11, 12^2, 12^{3b}, 15 / 8^1, 13^1, 15	-	-	- / -	-	-
	Nε2-**I**	18.4	11, 13, 13^1 / 12^2, 13^1	-	-	- / -	-	-
	Nε2-**II**	14.7	10, 12, 12^1, 12^{3a}, 13^1 / 12^1, 15	2.4	13.5	- / 12^1, 12^2	4.0	−1.2
**His-372**	Cδ2	-	- / -	-	11.5	12^3 / 12^2	4.0	-
	Cε1-**I**	10.4	19 / -	-	-	- / -	-	-
	Cε1-**II**	11.4	19 / -	4.8	9.9	12^3 / 13^1	4.3	−1.5
	Nε2-**II**	12.2	18, 19 / 18^1	3.3	12.8	12^2, 12^3, 13^1 / -	2.3	+0.6
**His-550**	Cα	6.0	- / 3^{1a}, 3^{2b}	4.3	-	- / -	-	-
	N	11.4	3^{2b} / 3^{1a}, 3^{2b}	4.7	-	- / -	-	-

Oat PhyA3–PCB

^{1}H contact (residue)		Pr: δ^{H}_{Pr} (ppm)	Pr: cofactor carbon (M/S)	Pr: HM distance (d_{min}, Å)	Pfr: δ^{H}_{Pfr} (ppm)	Pfr: cofactor carbon (M/S)	Pfr: HM distance (d_{min}, Å)	$\Delta\delta^{H}_{Pfr-Pr}$ (ppm)
**Ile-273**	Cα	4.3	10, 11 / 10, 13^{1}	4.0	4.8	9, 10, 14, 15 / 10, 15	4.2	+0.5
	Cβ	2.9	- / 15	5.5	-	- / -	-	-
	Cγ1	1.0	12, 14, 15, 16 / -	2.6	1.4	14, 15, 16, 17, 17^{1}, 18 / 15, 17^{1}	2.8	+0.4
	Cδ1	1.8	13, 13^{1}, 14, 15 / 17^{1}	3.0	2.0	10, 12, 13, 13^{1}, 14, 15 17, 17^{1} / 10, 15, 17^{1}	2.9	+0.2
**Ile-289**	Cδ1	-	- / -	-	1.9	- / 12^{2}	4.4	-
**Ile-67**	Cγ1	3.6	3^{1b}, 3^{2b} / 2	3.9	2.2	2^{1}, 3, 3^{1}, 3^{2} / 3, 3^{1}, 3^{2}	3.3	−1.4
	Cγ2	-	- / -	-	2.2	3, 3^{1}, 3^{2}, 5 / 3, 3^{1}, 5	3.5	-
	Cδ1	-	- / -	-	1.3	2, 3, 3^{2} / 2, 3, 3^{2}, 5	3.5	-
**Leu-368**	Cβ	-	- / -	-	2.9	13^{1}, 17^{1} / 13^{1}, 17^{1}, 18^{1}	3.3	-
	Cγ	2.3	- / 13^{1}	5.4	-	- / -	-	-
	Cδ1	1.7	12^{2}, 13^{1} / 13^{1}	3.2	1.8	17, 17^{1}, 18 / 12^{1}, 13^{1}, 17^{1}	2.8	+0.1
**Lys-242**	N	-	- / -	-	12.3	- / 18^{1}, 18^{2}	4.1	-
**Met-239**	Cε	3.1	18^{1}, 18^{2}, 19 / 18^{1}, 18^{2}	3.5	-	- / -	-	-
**Met-330**	Cε	-	- / -	-	3.3	12^{3} / 12^{2}	4.6	-
**Met-549**	Cα	5.2	3^{2b} / 3^{2b}	5.1	-	- / -	-	-
	Cβ	2.6	3^{1b}, 3^{2b} / 3^{2b}	3.2	-	- / -	-	-
	Cγ	-	- / -	-	4.3	- / 2	5.3	-
	Cε	2.8	3^{b}, 3^{1b}, 3^{2b} / -	3.2	2.9	1, 2 / 2, 3^{1}	3.7	+0.1
**Phe-243**	Cδ1	-	- / -	-	8.5	- / 18^{1}	4.8	-
	Cε1	-	- / -	-	7.9	17, 17^{1}, 18, 18^{1}, 18^{2} / 17^{1}, 18^{2}	3.7	-
	Cζ	-	- / -	-	7.0	17, 17^{1}, 18^{1} / 17^{1}, 18^{1}	4.0	-
**Phe-281**	Cε1	-	- / -	-	7.7	8^{3} / 8^{1}, 8^{2}	4.5	-
	Cζ	7.2	12, 12^{1}, 12^{2}, 12^{3} / 12^{1}	3.1	7.1	8, 8^{1}, 10 / 8^{1}, 10, 12^{1}	3.9	−0.1
**Pro-274**	Cβ	2.3	- / 5, 7^{1}	5.2	2.9	7 / 5, 7^{1}	4.7	+0.6
	Cγ	4.8	5, 6, 7, 7^{1} / 7^{1}, 8^{1}, 8^{2}	3.0	3.7	3, 5, 6, 7 / 2, 3, 3^{1}, 5	3.2	−1.1
	Cδ	-	5^{b}, 6, 8 / -	3.4	6.2	4, 5, 6, 7, 9 / 3, 5, 7^{1}, 10	3.1	−0.1
**Pro-319**	Cα	5.7	8^{2}, 8^{3} / -	3.9	5.8	8^{2}, 8^{3} / 8^{2}	3.5	+0.1
	Cβ	3.2	8^{1}, 8^{2}, 8^{3} / 8^{2}	3.7	2.9	8^{1}, 8^{2}, 8^{3} / 8^{1}	3.1	−0.3
**Pro-540**	Cβ	3.1	2, 2^{1} / -	3.9	-	- / -	-	-
**Pro-551**	Cα	4.5	2 / 2, 3, 3^{1b}, 3^{2b}	3.4	-	- / -	-	-
	Cβ	2.2	2 / 2, 3^{a}	3.6	-	- / -	-	-
	Cδ	6.4	- / 2, 2^{1}	4.9	-	- / -	-	-
**Ser-335**	Cβ	-	- / -	-	4.0	12^{2}, 12^{3} / 12^{2}	4.1	-
**Ser-541**	Cα	5.7	2^{1} / 2, 2^{1}	4.4	-	- / -	-	-
	Cβ	3.2	- / 2^{1}, 3^{2b}	4.9	-	- / -	-	-
**Ser-554**	Cβ	-	- / -	-	5.2	19 / 18^{2}	4.9	-
	Oγ	-	- / -	-	6.8	19 / -	5.3	-
**Ser-63**	Oγ	-	- / -	-	6.9	2^{1} / 2, 2^{1}	3.9	-
**Thr-271**	Cβ	4.8	2^{1} / 2	3.6	4.7	2, 2^{1} / -	3.5	−0.1
	Cγ2	2.1	- / 2^{1}	5.7	-	- / -	-	-
	Oγ1	9.4	- / 2^{1}	5.9	9.7	1 / 2, 2^{1}	4.4	+0.3
**Tyr-241***	Cβ	-	18^{1}, 18^{2} / -	2.8	3.2	17^{1}, 18^{1} / 17^{1}, 18^{2}	2.7	−0.6

*Continued on following page.

Oat PhyA3–PCB

		Pr			Pfr			
^{1}H contact (residue)		δ^{H}_{Pr} (ppm)	cofactor carbon (M/S)	HM distance (d_{min}, Å)	δ^{H}_{Pfr} (ppm)	cofactor carbon (M/S)	HM distance (d_{min}, Å)	$\Delta\delta^{H}_{Pfr-Pr}$ (ppm)
**Tyr-241**	Cδ1	10.4	18, 18^{1}, 18^{2} / 18^{2}	3.0	-	- / -	-	-
	Cδ2	-	- / -	-	8.6	17, 17^{1}, 18, 18^{1} / 13^{1}, 17^{1}	2.8	-
	Cε1	9.0	17^{1}, 18^{1}, 18^{2} / 17^{1}, 18^{1}, 18^{2}	3.9	-	- / -	-	-
	Cε2	-	- / -	-	8.8	13, 13^{1}, 17^{1} / 15, 17^{1}	3.6	-
**Tyr-268**	Cβ	3.8	- / 17^{1}, 18^{2}	3.4	3.7	16, 17, 18 / -	3.8	**−0.1**
	Cδ1	11.4	- / 17^{1}, 18^{2}	4.5	-	- / -	-	-
	Cε1	9.6	17^{1}, 18, 18^{1} / -	5.1	-	- / -	-	-
	Cε2	9.7	18^{1}, 18^{2} / -	4.3	10.5	18 / -	4.8	**+0.8**
	Oη	8.0	18^{1}, 18^{2} / 18^{1}, 18^{2}	5.1	-	- / -	-	-
**Tyr-326**	Cβ	4.0	1 [a] / -	4.5	-	- / -	-	-
	Cδ1	10.6	1 [b] / -	3.5	10.5	1 / 2	3.8	**−0.1**
	Cδ2	11.0	- / 10	5.2	-	- / -	-	-
	Cε1	11.8	1 [a], 17^{1} / 2, 15	4.2	11.7	1 / -	3.5	**−0.1**
	Cε2	11.2	15, 17 / -	4.1	10.8	14, 15 / 15	4.3	**−0.4**
	Oη	8.3	15, 16, 17, 17^{1} / 17^{1}, 18^{1}	4.0	10.0	15, 16 / 15	5.2	**+1.7**
**Tyr-69**	Cδ2	-	- / -	-	9.4	3^{2} / 3^{1}	4.2	-
	Cε2	-	- / -	-	10.1	3, 3^{1}, 3^{2} / 3, 3^{2}	2.8	-
**Val-251**	Cα	4.5	- / 18^{2}	5.8	-	- / -	-	-
	Cγ2	1.1	18^{1}, 18^{2} / -	3.5	-	- / -	-	-
**Val-337**	Cβ	2.2	- / 12^{1}, 12^{2}	4.2	2.3	12^{2}, 12^{3} / -	4.0	**+0.1**
	Cγ1	0.9	12^{2}, 13^{1} / -	4.0	1.1	12, 12^{1}, 12^{2} / 12^{1}	3.4	**+0.2**
	Cγ2	1.3	12^{1}, 12^{2}, 12^{3a} / 12^{1}, 12^{2}	2.7	-	- / -	-	-
**Val-370**	Cβ	2.2	13^{1}, 19 / -	4.4	-	- / -	-	-
	Cγ1	0.9	13^{1}, 19 / 13^{1}	2.8	1.1	12^{3}, 13 / 12^{2}, 13^{1}	3.0	**+0.2**
**W1**	W1-**I**	7.0	1, 2, 5 [a], 10 / 5 [a], 10	-	-	- / -	-	-
	W1-**II**	7.9	1 [a], 4, 5, 10, 11, 15 / 2, 10, 15	3.6	7.8	1, 4, 6, 9 / -	3.6	**−0.1**
**W2**		8.0	12^{3b} / -	3.3	-	- / -	-	-
**W3**		8.1	12^{2}, 12^{3} / -	3.3	-	- / -	-	-
**W4 (Pr-II)**		8.9	12^{2}, 12^{3b} / 12^{2}	3.9	-	- / -	-	-
**W5 (Pr-II)**		8.8	12^{3a}, 13, 19 / 15	-	-	- / -	-	-
**{W5+$[OH]^{-}_{W4}$}(Pr-I)**		9.6	12^{1}, 13, 14, 16, 19 / 12^{1}	-	-	- / -	-	-

Supplementary Table S10. Reassigning the interfacial ^{1}H contacts of the PCB carbon atoms in the Pr state. The MELODI–HETCOR data were obtained from *u*-[^{13}C,^{15}N]-PCB-*As*.phyA3 with an LG–CP contact time of 2.3 ms (Song et al., 2012) and the tentative ^{1}H assignment was based on the structural model for the PΦB adduct of *As*.phyA3 (Mroginski et al., 2011). Here, the reassignment of ^{1}H contacts was based on the model for the *As*.phyA3–PCB adduct. The $^{1}H^{residue}$–$^{13}C^{PCB}$ distances ("HM distance") were extracted from the two QM/MM models. Model-derived changes on the ^{1}H assignment are tracked by colors and shown in Supplementary Figure S4.

		Oat PhyA3–PCB Pr					
		Structural model for the native PΦB adduct			Structural model for the PCB adduct		
cofactor carbon	δ^{C}_{Pr} (ppm)	^{1}H contact (residue)	δ^{H}_{Pr} (ppm)	HM distance (Å)	^{1}H contact (residue)	δ^{H}_{Pr} (ppm)	HM distance (Å)
**1**	**182.4[a]/183.2[b]**	Asp-272 Cα[a]	5.2	3.8	Asp-272 Cα[a]	5.2	3.3
		Asp-272 N[a]	12.5	4.6	Asp-272 N[a]	12.5	5.8
		Tyr-326 Cβ[a]	4.0	5.0	Tyr-326 Cβ[a]	4.0	4.5
					Tyr-326 Cδ1[b]	10.6	3.5
		Tyr-326 Cδ2[b]	10.6	4.1			
					Tyr-326 Cε1[a]	11.8	4.2
		Tyr-326 Cε2[a]	11.8	4.6			
		W1-**I**	7.0	-	W1-**I**	7.0	-
		W1-**II**[a]	7.9	-	W1-**II**[a]	7.9	3.6
**2**	**36.9**	Asp-272 Cα	5.2	4.8	Asp-272 Cα	5.2	4.2
		Asp-542 Cβ	3.1	4.6			
					Pro-540 Cβ	3.1	5.3
		Pro-551 Cα	4.5	4.6	Pro-551 Cα	4.5	3.4
		Pro-551 Cβ	2.2	5.0	Pro-551 Cβ	2.2	3.6
		W1-**I**	7.0	-	W1-**I**	7.0	-
**2[1]**	**17.6**	Asp-272 N	12.5	4.7	Asp-272 N	12.5	5.4
		Asp-542 Cβ	3.1	3.8			
					Pro-540 Cβ	3.1	3.9
					Ser-541 Cα	5.7	4.9
		Thr-271 Cα	5.7	4.8			
		Thr-271 Cβ	4.8	4.2	Thr-271 Cβ	4.8	3.6
		Thr-271 Oγ1	9.4	4.0	-	-	-
**3**	**53.9[a]/55.8[b]**	Cys-322 Cβ	4.5	4.4	Cys-322 Cβ	4.5	3.8
		-	-	-	Met-549 Cε[b]	2.8	5.0
**3[1]**	**47.7[a]/48.9[b]**	Cys-322 Cα[b]	6.2	4.3	Cys-322 Cα[b]	6.2	4.7
		Cys-322 Cβ	4.5	3.0	Cys-322 Cβ	4.5	3.0
		His-323 N[b]	11.7	4.9	-	-	-
					Ile-67 Cγ1[b]	3.6	3.9
		Met-549 Cβ[b]	2.6	4.3	Met-549 Cβ[b]	2.6	4.4
		Met-549 Cγ[b]	3.6	4.8	-	-	-
		-	-	-	Met-549 Cε[b]	2.8	4.1
**3[2]**	**23.0[a]/21.6[b]**	Cys-322 Cα[b]	6.2	4.8	Cys-322 Cα[b]	6.2	5.4
		Cys-322 Cβ[b]	4.5	3.5	Cys-322 Cβ[b]	4.5	3.8
		His-550 N[b]	11.4	3.9	His-550 N[b]	11.4	4.7
					Ile-67 Cγ1[b]	3.6	3.9
		Met-549 Cα[b]	5.2	4.2	Met-549 Cα[b]	5.2	5.1
		Met-549 Cβ[b]	2.6	3.0	Met-549 Cβ[b]	2.6	3.2
		Met-549 Cγ[b]	3.6	3.7			
		-	-	-	Met-549 Cε[b]	2.8	3.2
**4**	**153.1**	Asp-272 Cα	5.2	3.8	Asp-272 Cα	5.2	4.5
		Asp-272 Cβ	3.3	4.3	-	-	-
		W1-**II**	7.9	-	W1-**II**	7.9	3.8
**5**	**85.4[a]/86.1[b]**	Pro-274 Cγ	4.8	4.0	Pro-274 Cγ	4.8	3.5
		Pro-274 Cδ[b]	6.3	4.1	Pro-274 Cδ[b]	6.3	3.7
		W1-**I**[a]	7.0	-	W1-**I**[a]	7.0	-
		W1-**II**	7.9	-	W1-**II**	7.9	4.2
**6**	**155.9**	His-323 Cα	5.5	4.6	His-323 Cα	5.5	4.3
		His-323 N	11.7	4.7	His-323 N	11.7	4.2
		Pro-274 Cγ	4.8	3.7	Pro-274 Cγ	4.8	3.0
		Pro-274 Cδ	6.3	3.6	Pro-274 Cδ	6.3	3.4

Oat PhyA3–PCB Pr

cofactor carbon	δ^{C}_{Pr} (ppm)	Structural model for the native PΦB adduct			Structural model for the PCB adduct		
		^{1}H contact (residue)	δ^{H}_{Pr} (ppm)	HM distance (Å)	^{1}H contact (residue)	δ^{H}_{Pr} (ppm)	HM distance (Å)
**7**	**124.3**	His-320 Cβ	3.2	4.5	His-320 Cβ	3.2	3.6
		His-323 Cα	5.5	4.8	His-323 Cα	5.5	4.9
		His-323 Cβ	4.0	4.1	His-323 Cβ	4.0	3.1
		His-323 Nδ1-**I**	13.6	-	-	-	-
		Pro-274 Cγ	4.8	3.9	Pro-274 Cγ	4.8	3.0
**7^{1}**	**7.3**	-	-	-	Cys-322 Cβ	4.5	3.6
		His-320 Cα	6.0	4.8	-	-	-
		His-320 Cβ	3.2	3.5	His-320 Cβ	3.2	2.8
		His-323 Cβ	4.0	4.9	-	-	-
		His-323 N	11.7	5.0	His-323 N	11.7	4.3
		Pro-274 Cγ	4.8	5.0	Pro-274 Cγ	4.8	3.1
**8**	**146.0**	Ala-277 Cβ	2.0	4.1	Ala-277 Cβ	2.0	4.1
		His-323 Cβ	4.0	3.9	His-323 Cβ	4.0	3.9
		Pro-274 Cδ	6.3	4.8	Pro-274 Cδ	6.3	4.8
**8^{1}**	**22.0**	-	-	-	Arg-317 Nη1	10.0	4.4
		His-320 Cβ	3.2	5.1	His-320 Cβ	3.2	3.4
		His-320 N	12.0	4.9	His-320 N	11.9	3.1
		His-323 Cδ2-**II**	12.8	5.0	His-323 Cδ2-**II**	12.8	4.9
		His-323 Nε2-**II**	14.7	5.5	-	-	-
		-	-	-	Pro-319 Cβ	3.2	3.7
**8^{2}**	**41.4**				Ala-277 Cα	3.8	4.5
		Gln-71 Cβ	3.8	-			
		His-320 Cα	6.0	4.5	-	-	-
		Pro-319 Cα	5.7	4.6	Pro-319 Cα	5.7	5.0
		Pro-319 Cβ	3.1	4.7	Pro-319 Cβ	3.2	4.6
**8^{3}**	**179.6**				Ala-277 Cα	3.8	4.5
					Ala-277 Cβ	2.0	3.8
		Arg-287 Nη1	9.2	4.0	Arg-287 Nη1	9.2	7.4
		Arg-317 Nη1	10.0	3.8	Arg-317 Nη1	10.0	2.4
		Arg-317 Nη2	7.2	4.5	Arg-317 Nη2	7.2	2.5
		Gln-71 Cβ	3.8	-			
		His-320 N	12.0	3.7	His-320 N	11.9	3.5
		Leu-280 Cδ2	2.0	4.9			
		Pro-319 Cα	5.7	3.9	Pro-319 Cα	5.7	3.9
		Pro-319 Cβ	3.1	4.1	Pro-319 Cβ	3.2	4.4
		W2	8.0	-	-	-	-
**9**	**128.2**	Ala-277 Cβ	2.0	4.3	Ala-277 Cβ	2.0	4.7
		His-323 Cα	5.5	4.8	His-323 Cα	5.5	4.3
		His-323 Cβ	4.0	3.9	His-323 Cβ	4.0	2.5
		His-323 Cε1-**I**	16.9	-	His-323 Cε1-**I**	13.6	-
		His-323 Nδ1-**I**	13.6	-			
		-	-	-	His-323 N	11.7	4.6
		Pro-274 N	11.0	5.2	-	-	-
**10**	**113.0**	-	-	-	His-323 Cβ	4.0	3.1
		His-323 Cδ2-**I**	16.1	-	His-323 Cδ2-**I**	16.1	-
		His-323 Cδ2-**II**	12.8	4.4	-	-	-
					His-323 Cε1-**I**	13.6	-
		His-323 Cε1-**II**	12.1	3.5	His-323 Cε1-**II**	12.1	4.0
		His-323 Nδ1-**I**	13.6	-			
		His-323 Nε2-**II**	14.7	4.2	His-323 Nε2-**II**	14.7	4.4
		Ile-273 Cα	4.3	4.8	Ile-273 Cα	4.3	4.4
		W1-**I**	7.0	-	W1-**I**	7.0	-
		W1-**II**	7.9	-	W1-**II**	7.9	4.2

Oat PhyA3–PCB Pr

cofactor carbon	δ^{C}_{Pr} (ppm)	Structural model for the native PΦB adduct: ^{1}H contact (residue)	δ^{H}_{Pr} (ppm)	HM distance (Å)	Structural model for the PCB adduct: ^{1}H contact (residue)	δ^{H}_{Pr} (ppm)	HM distance (Å)
**11**	**127.4**	Ala-277 Cβ	2.0	5.0	Ala-277 Cβ	2.0	4.8
		His-323 Cδ2-**I**	16.1	-	His-323 Cδ2-**I**	16.1	-
		His-323 Cδ2-**II**	12.8	4.7	His-323 Cδ2-**II**	12.8	4.9
		His-323 Cε1-**I**	16.9	-			
		His-323 Cε1-**II**	12.1	3.1	His-323 Cε1-**II**	12.1	3.2
					His-323 Nδ1-**I**	16.9	-
		His-323 Nε2-**I**	18.4	-	His-323 Nε2-**I**	18.4	-
		Ile-273 Cα	4.3	4.5	Ile-273 Cα	4.3	4.0
		Ile-273 Cβ	2.9	4.6	-	-	-
		Ile-273 N	11.2	5.1	-	-	-
		W1-**II**	7.9	-	W1-**II**	7.9	3.9
**12**	**144.1**	His-323 Cδ2-**I**	16.1	-	His-323 Cδ2-**I**	16.1	-
		His-323 Cδ2-**II**	12.8	4.9	His-323 Cδ2-**II**	12.8	5.1
		His-323 Cε1-**II**	12.1	3.4	His-323 Cε1-**II**	12.1	3.2
					His-323 Cε1-**I**	13.6	-
		His-323 Nδ1-**I**	13.6	-			
		His-323 Nε2-**II**	14.7	4.1	His-323 Nε2-**II**	14.7	3.7
		Ile-273 Cγ1	1.0	4.2	Ile-273 Cγ1	1.0	3.8
		Phe-281 Cε2	7.2	4.9			
					Phe-281 Cζ	7.2	4.2
		W4 (**Pr-II**)	8.9	-	-	-	-
**12^1**	**22.8**				Ala-277 Cβ	2.0	4.3
		Phe-281 Cε2	7.2	4.0			
		-	-	-	His-323 Nε2-**II**	14.7	3.7
					Phe-281 Cζ	7.2	3.2
		Val-337 Cβ	2.0	5.1			
		Val-337 Cγ1	1.3	4.8			
					Val-337 Cγ2	1.3	4.1
		{W5+$[OH]^{-}_{W4}$} (**Pr-I**)	9.6	-	{W5+$[OH]^{-}_{W4}$} (**Pr-I**)	9.6	-
**12^2**	**42.1**	Arg-287 Cβ	2.8	4.7	Arg-287 Cβ	2.9	4.7
		His-323 Cε1-**I**	16.9	-			
		His-323 Cε1-**II**	12.1	4.5	His-323 Cε1-**II**	12.1	4.2
					His-323 Nδ1-**I**	16.9	-
		Leu-368 Cδ1	1.7	4.9	Leu-368 Cδ1	1.7	4.9
		Phe-281 Cε1	7.2	4.6	-	-	-
		Phe-281 Cε2	7.2	4.2	-	-	-
					Phe-281 Cζ	7.2	3.1
		Phe-281 Cζ	8.1	3.6	-	-	-
		Val-337 Cβ	2.0	3.7	-	-	-
					Val-337 Cγ1	0.9	4.0
		Val-337 Cγ2	0.9	3.9			
		-	-	-	Val-337 Cγ2	1.3	2.7
		W3	8.1	-	W3	8.1	4.4
		W4 (**Pr-II**)	8.9	-	W4 (**Pr-II**)	8.9	4.4
**12^3***	**176.7[a]/177.2[b]**	Arg-287 Cβ[a]	2.8	4.5	Arg-287 Cβ[a]	2.9	4.1
		His-323 Cδ2-**II**[a]	12.8	4.3	-	-	-
		His-323 Cε1-**I**[b]	16.9	-	His-323 Cε1-**I**[b]	13.6	-
		His-323 Cε1-**II**[a]	12.1	4.0	-	-	-
		His-323 Nδ1-**I**[b]	13.6	-	His-323 Nδ1-**I**[b]	16.9	-
		His-323 Nε2-**II**[a]	14.7	3.5	His-323 Nε2-**II**[a]	14.7	2.4
		Met-327 Cε[a]	3.7	4.5	-	-	-
		Phe-281 Cε1	7.2	4.7			
					Phe-281 Cζ	7.2	3.2
		Ser-335 Oγ	7.2	4.6	-	-	-
		Val-337 Cγ1[a]	1.3	3.7			
					Val-337 Cγ2[a]	1.3	3.4

*Continued on following page.

		Oat PhyA3–PCB Pr					
		Structural model for the native PΦB adduct			Structural model for the PCB adduct		
cofactor carbon	δ^{C}_{Pr} (ppm)	^{1}H contact (residue)	δ^{H}_{Pr} (ppm)	HM distance (Å)	^{1}H contact (residue)	δ^{H}_{Pr} (ppm)	HM distance (Å)
**12^{3}**	**176.7[a]/177.2[b]**	W2[b]	8.0	-	W2[b]	8.0	3.3
		W3	8.1	-	W3	8.1	3.3
		W4 (**Pr-II**)[a]	8.9	-	W4 (**Pr-II**)[b]	8.9	3.9
		W5 (**Pr-II**)[a]	8.8	-	W5 (**Pr-II**)[a]	8.8	-
**13**	**123.4**	His-323 Cδ2-**I**	16.1	-	His-323 Cδ2-**I**	16.1	-
		His-323 Nε2-**I**	18.4	-	His-323 Nε2-**I**	18.4	-
		Ile-273 Cδ1	1.8	3.9	Ile-273 Cδ1	1.8	4.2
		W5 (**Pr-II**)	8.8	-	W5 (**Pr-II**)	8.8	-
		{W5+[OH]$^{-}_{W4}$} (**Pr-I**)	9.6	-	{W5+[OH]$^{-}_{W4}$} (**Pr-I**)	9.6	-
**13^{1}**	**10.3**	His-323 Nε2-**I**	18.4	-	His-323 Nε2-**I**	18.4	-
		His-323 Nε2-**II**	14.7	5.4	His-323 Nε2-**II**	14.7	5.0
		-	-	-	Ile-273 Cδ1	1.8	3.6
		Leu-368 Cδ1	1.7	4.5	Leu-368 Cδ1	1.7	3.2
					Val-337 Cγ1	0.9	4.2
		Val-337 Cγ2	0.9	4.4			
		Val-370 Cβ	2.2	4.8	Val-370 Cβ	2.2	4.4
		Val-370 Cγ1	0.9	4.4	Val-370 Cγ1	0.9	4.8
		Val-370 Cγ2	0.9	4.0	-	-	-
		W6	7.7	-	-	-	-
**14**	**147.1**	His-323 Cε1-**II**	12.1	3.8	His-323 Cε1-**II**	12.1	3.0
		Ile-273 Cβ	2.9	4.5	-	-	-
		Ile-273 Cγ1	1.0	3.2	Ile-273 Cγ1	1.0	2.6
		Ile-273 Cγ2	1.0	5.1	-	-	-
		Ile-273 Cδ1	1.8	3.8	Ile-273 Cδ1	1.8	4.2
		{W5+[OH]$^{-}_{W4}$} (**Pr-I**)	9.6	-	{W5+[OH]$^{-}_{W4}$} (**Pr-I**)	9.6	-
**15**	**93.8**	Asp-272 Cβ	3.3	4.6	Asp-272 Cβ	3.5	3.6
					His-323 Cε1-**I**	13.6	-
		His-323 Cε1-**II**	12.1	4.6	His-323 Cε1-**II**	12.1	3.7
		His-323 Nδ1-**I**	13.6	-			
		Ile-273 Cγ1	1.0	3.6	Ile-273 Cγ1	1.0	2.6
		Ile-273 Cδ1	1.8	4.0	Ile-273 Cδ1	1.8	3.6
		Tyr-326 Cε1	11.2	4.6			
					Tyr-326 Cε2	11.2	4.3
		Tyr-326 Oη	8.3	4.6	Tyr-326 Oη	8.3	4.9
		W1-**II**	7.9	-	W1-**II**	7.9	3.7
**16**	**146.3**	-	-	-	Ile-273 Cγ1	1.0	3.6
		Tyr-326 Oη	8.3	4.4	Tyr-326 Oη	8.3	4.6
		{W5+[OH]$^{-}_{W4}$} (**Pr-I**)	9.6	-	{W5+[OH]$^{-}_{W4}$} (**Pr-I**)	9.6	-
**17**	**143.0**	Tyr-268 Cδ2	10.5	4.7	-	-	-
		Tyr-326 Cε1	11.2	4.3			
					Tyr-326 Cε2	11.2	4.1
		Tyr-326 Oη	8.3	3.5	Tyr-326 Oη	8.3	4.3
**17^{1}**	**10.1**				Asp-272 Cβ	3.5	2.9
		Tyr-241 Cε1	8.9	5.0	Tyr-241 Cε1	9.0	5.0
		Tyr-268 Cβ	3.5	4.2			
					Tyr-268 Cε1	9.6	5.1
		Tyr-268 Cε2	9.6	4.0			
					Tyr-326 Cε1	11.8	5.0
		Tyr-326 Cε2	11.8	4.7			
		Tyr-326 Oη	8.3	2.7	Tyr-326 Oη	8.3	4.0

		Oat PhyA3–PCB Pr					
		**Structural model for the native PΦB adduct**			**Structural model for the PCB adduct**		
cofactor carbon	δ^{C}_{Pr} (ppm)	^{1}H contact (residue)	δ^{H}_{Pr} (ppm)	HM distance (Å)	^{1}H contact (residue)	δ^{H}_{Pr} (ppm)	HM distance (Å)
**18**	**133.1**	His-372 Cε1	10.4	4.4			
		His-372 Nδ1 (**Pr-I**)	12.2	5.0			
					His-372 Nε2 (**Pr-II**)	12.2	4.7
		Met-320 Cε	3.8	4.7	-	-	-
					Tyr-241 Cδ1	10.4	5.6
		Tyr-268 Cδ2	10.5	4.9	-	-	-
					Tyr-268 Cε1	9.6	5.7
		Tyr-268 Cε2	9.6	4.7			
**18^{1}**	**16.7**	His-372 Cε1 (**Pr-I**)	10.4	4.7			
		His-372 Cε1 (**Pr-II**)	9.0	4.7			
		Met-239 Cε	3.1	4.6	Met-239 Cε	3.1	3.8
		Met-320 Cε	3.8	4.3			
					Tyr-241 Cβ	3.8	4.3
					Tyr-241 Cδ1	10.4	4.3
					Tyr-241 Cε1	9.0	4.5
		Tyr-268 Cδ2	10.5	4.2	-	-	-
		Tyr-268 Cε1	9.7	4.0	Tyr-268 Cε1	9.6	5.3
		Tyr-268 Cε2	9.6	3.7	Tyr-268 Cε2	9.7	4.3
		Tyr-268 Oη	8.0	3.8	Tyr-268 Oη	8.0	5.1
		Val-251 Cγ2	1.1	4.8	Val-251 Cγ2	1.1	4.2
**18^{2}**	**12.9**	His-372 Cε1 (**Pr-I**)	10.4	3.7			
		His-372 Cε1 (**Pr-II**)	9.0	3.7			
		His-372 Nδ1 (**Pr-I**)	12.2	4.5	-	-	-
		His-372 Nε2 (**Pr-II**)	11.5	4.4	-	-	-
		Met-239 Cε	3.1	3.4	Met-239 Cε	3.1	3.5
		Met-320 Cε	3.8	4.2			
					Tyr-241 Cβ	3.8	2.8
					Tyr-241 Cδ1	10.4	3.0
					Tyr-241 Cε1	9.0	3.9
		Tyr-263 Cβ	4.5	4.9	-	-	-
		Tyr-263 Cδ2	8.9	4.0	-	-	-
		Tyr-268 Cε1	9.7	4.9			
					Tyr-268 Cε2	9.7	4.7
		Tyr-268 Oη	8.0	4.0	Tyr-268 Oη	8.0	5.9
		Val-251 Cγ2	1.1	4.9	Val-251 Cγ2	1.1	3.5
**19**	**172.4**	His-372 Cε1 (**Pr-I**)	10.4	3.9	His-372 Cε1 (**Pr-I**)	10.4	-
		His-372 Cε1 (**Pr-II**)	9.0	3.9	-	-	-
					His-372 Cε1 (**Pr-II**)	11.4	5.3
		His-372 Nδ1 (**Pr-I**)	12.2	5.2			
		His-372 Nε2 (**Pr-II**)	11.5	3.7	His-372 Nε2 (**Pr-II**)	12.2	3.3
		Met-239 Cε	3.1	4.7	Met-239 Cε	3.1	5.4
		Val-370 Cβ	2.2	4.5	Val-370 Cβ	2.2	5.0
		Val-370 Cγ1	0.9	3.9	Val-370 Cγ1	0.9	2.8
		Val-370 Cγ2	0.9	4.7	-	-	-
		W5 (**Pr-II**)	8.8	-	W5 (**Pr-II**)	8.8	-
		{W5+$[OH]^{-}_{W4}$} (**Pr-I**)	9.6	-	{W5+$[OH]^{-}_{W4}$} (**Pr-I**)	9.6	-

Supplementary Table S11. Reassigning the interfacial 2.5–6.5 Å ^{1}H contacts of the non-quaternary PCB carbon atoms as Pr. The SIDY data were obtained from *u*-[^{13}C,^{15}N]-PCB-*As*.phyA3 with a ^{1}H spin-diffusion mixing time of 800 μs and a CP contact time of 96 μs (Song et al., 2012), however the tentative ^{1}H assignment was based on the structural model for the PΦB adduct of *As*.phyA3 (Mroginski et al., 2011). Here, the reassignment of ^{1}H SIDY contacts of the PCB carbons is based on the structural model for the *As*.phyA3–PCB adduct. The ^{1}H^{residue}–^{13}C^{PCB} distances ("HM distance") were extracted from the two QM/MM models. Model-derived changes on the ^{1}H assignment are tracked by colors and shown in Supplementary Figure S5.

		Oat PhyA3–PCB Pr					
		Structural model for the native PΦB adduct			Structural model for the PCB adduct		
cofactor carbon	δ^C_{Pr} (ppm)	^{1}H contact (residue)	δ^H_{Pr} (ppm)	HM distance (Å)	^{1}H contact (residue)	δ^H_{Pr} (ppm)	HM distance (Å)
**2**	**36.9**				Arg-552 N	12.5	5.0
		Asp-272 N	12.5	5.3			
		Asp-542 Cα	5.7	5.7			
					Ile-67 Cγ1	3.6	5.1
		-	-	-	Pro-551 Cα	4.5	3.4
		Pro-551 Cβ	2.2	5.0	Pro-551 Cβ	2.2	3.6
		Pro-551 Cδ	6.4	5.7	Pro-551 Cδ	6.4	4.9
		Met-549 Cγ	3.6	5.9			
					Ser-541 Cα	5.7	4.9
		Thr-271 Cβ	4.8	5.5	Thr-271 Cβ	4.8	5.0
		Tyr-326 Cβ	4.0	5.9	-	-	-
					Tyr-326 Cε1	11.8	5.5
		Tyr-326 Cε2	11.8	5.2			
		Val-66 Cγ1	1.4	-	-	-	-
		W1-**II**	7.9	-	W1-**II**	7.9	5.0
**2^1**	**17.6**				Arg-552 N	12.5	4.9
					Asp-272 N	12.5	5.4
		Asp-542 Cα	5.7	5.0			
		Asp-542 Cβ	3.1	3.8			
		Met-549 Cε	3.9	5.6	-	-	-
		Pro-551 Cδ	6.4	6.1	Pro-551 Cδ	6.4	6.2
		Pro-551 N	12.5	5.9			
					Ser-541 Cα	5.7	4.4
					Ser-541 Cβ	3.2	4.9
		Thr-271 Cγ2	2.1	5.4	Thr-271 Cγ2	2.1	5.7
		Thr-271 Oγ1	9.4	4.0	Thr-271 Oγ1	9.4	5.9
**3**	**53.9^a/55.8^b**				Asp-272 N^a	12.5	6.1
		-	-	-	Cys-322 Cβ	4.5	3.8
		Pro-551 Cα	4.5	5.5	Pro-551 Cα	4.5	4.6
		Pro-551 Cβ^a	2.2	5.7	Pro-551 Cβ^a	2.2	4.6
		Pro-551 N^a	12.5	5.9	-	-	-
**3^1**	**47.7^a/48.9^b**	Cys-322 Cβ^b	4.5	3.0	Cys-322 Cβ^b	4.5	3.0
		His-323 N	11.7	4.9	His-323 N	11.7	5.6
		His-550 Cα^a	6.0	5.6	His-550 Cα^a	6.0	4.8
		His-550 N^a	11.4	5.4	His-550 N^a	11.4	5.9
		Met-549 Cα^b	5.2	5.6	-	-	-
					Pro-551 Cα^b	4.5	4.7
		Pro-551 N^b	12.5	5.2	-	-	-
**3^2**	**23.0^a/21.6^b**	Asp-542 Cβ^b	3.1	5.2			
		Cys-322 Cα^b	6.2	4.8	Cys-322 Cα^b	6.2	5.4
		-	-	-	Cys-322 Cβ^b	4.5	3.8
		His-550 Cα^b	6.0	4.4	His-550 Cα^b	6.0	4.3
		His-550 N^b	11.4	3.9	His-550 N^b	11.4	4.7
		Met-549 Cα^b	5.2	4.2	Met-549 Cα^b	5.2	5.1
		Met-549 Cβ^b	2.6	3.0	Met-549 Cβ^b	2.6	3.2
		Met-549 Cε^b	3.9	5.0	-	-	-
		Pro-551 Cα^b	4.5	4.7	Pro-551 Cα^b	4.5	4.5
					Ser-541 Cβ^b	3.2	5.3

	Oat PhyA3–PCB Pr						
		Structural model for the native PΦB adduct			Structural model for the PCB adduct		
cofactor carbon	δ^{C}_{Pr} (ppm)	^{1}H contact (residue)	δ^{H}_{Pr} (ppm)	HM distance (Å)	^{1}H contact (residue)	δ^{H}_{Pr} (ppm)	HM distance (Å)
**5**	**85.4[a]/86.1[b]**	Asp-272 Cα[b]	5.2	5.5	Asp-272 Cα[b]	5.2	5.4
		Cys-322 Cα[a]	6.2	5.6	Cys-322 Cα[a]	6.2	5.8
		Cys-322 Cβ[a]	4.5	4.6	Cys-322 Cβ[a]	4.5	3.0
					Cys-322 N[b]	11.0	5.6
		His-323 Cα[a]	5.5	5.0	His-323 Cα[a]	5.5	4.8
		His-323 Cβ[a]	4.0	5.1	His-323 Cβ[a]	4.0	4.4
		His-323 N[b]	11.7	4.8	His-323 N[b]	11.7	4.6
		Ile-273 N[a]	11.2	5.9	-	-	-
		Pro-274 Cβ	2.3	5.1	Pro-274 Cβ	2.3	5.7
		Pro-274 N[b]	11.0	5.4	-	-	-
		W1-**I**[a]	7.0	-	W1-**I**[a]	7.0	-
**7[1]**	**7.3**	Cys-322 N	11.0	5.9	Cys-322 N	11.0	4.9
		Gln-71 Cβ	3.8	5.8	-	-	-
					His-320 Cβ	3.2	2.8
		His-320 Cε1	12.0	5.7	-	-	-
		-	-	-	His-320 N	12.0	4.8
		His-320 Nε2	13.2	5.7	His-320 Nε2	13.2	5.9
		Pro-274 Cβ	2.3	5.6	Pro-274 Cβ	2.3	5.2
		Pro-274 Cγ	4.8	5.0	Pro-274 Cγ	4.8	3.1
		Tyr-69 Cβ	3.2	5.5			
**8[1]**	**22.0**	Ala-277 N	12.0	5.6			
		Arg-287 Nη1	9.2	5.6	-	-	-
		Arg-317 Nη2	7.2	6.1	Arg-317 Nη2	7.2	4.9
		-	-	-	His-320 Cβ	3.2	3.4
					His-320 N	12.0	3.1
		His-323 Cδ2-**II**	12.8	5.0	His-323 Cδ2-**II**	12.8	4.9
		His-323 Cε1-**I**	16.9	-			
					His-323 Nδ1-**I**	16.9	-
		His-323 Nε2-**II**	14.7	5.5	-	-	-
		Pro-274 Cγ	4.8	5.4	Pro-274 Cγ	4.8	4.2
**8[2]**	**41.4**	Ala-277 Cα	4.8	5.4			
		Arg-317 Nη1	10.0	5.2	Arg-317 Nη1	10.0	3.9
		Arg-317 Nη2	7.2	5.3	Arg-317 Nη2	7.2	3.9
		His-320 N	12.0	3.8	His-320 N	12.0	3.9
					Pro-274 Cγ	4.8	4.1
		Pro-319 Cβ	3.1	4.7	Pro-319 Cβ	3.2	4.6
		W3	8.1	-	-	-	-
**10**	**113.0**	Ala-277 Cα	4.8	5.7	-	-	-
		Ala-277 Cβ	2.0	4.2	Ala-277 Cβ	2.0	4.4
		His-323 Cα	5.5	5.4	His-323 Cα	5.5	4.7
		His-323 Cβ	4.0	4.3	His-323 Cβ	4.0	3.1
		His-323 Cδ2-**I**	16.1	-	His-323 Cδ2-**I**	16.1	-
		His-323 Cε1-**II**	12.1	3.5	His-323 Cε1-**II**	12.1	4.0
		Ile-273 Cα	4.3	4.8	Ile-273 Cα	4.3	4.4
		Pro-274 N	11.0	5.7			
					Tyr-326 Cδ2	11.0	5.2
		W1-**I**	7.0	-	W1-**I**	7.0	-
		W1-**II**	7.9	-	W1-**II**	7.9	4.2

Oat PhyA3–PCB Pr

cofactor carbon	δ^{C}_{Pr} (ppm)	Structural model for the native PΦB adduct: ^{1}H contact (residue)	δ^{H}_{Pr} (ppm)	HM distance (Å)	Structural model for the PCB adduct: ^{1}H contact (residue)	δ^{H}_{Pr} (ppm)	HM distance (Å)
**12^1**	**22.8**				Arg-287 Cβ	2.8	5.7
		Arg-287 Cγ	2.2	5.8			
		His-323 Cδ2-**II**	12.8	4.9	His-323 Cδ2-**II**	12.8	5.0
		-	-	-	His-323 Nε2-**II**	14.7	4.1
		Ile-273 Cβ	2.8	6.0			
		Phe-281 Cε2	7.2	4.0	-	-	-
		Phe-281 Cζ	8.1	3.8	Phe-281 Cζ	7.2	3.1
					Val-337 Cβ	2.2	5.5
		Val-337 Cγ1	1.3	4.8			
					Val-337 Cγ2	1.3	4.1
		{W5+$[OH]^{-}_{W4}$} (**Pr-I**)	9.6	-	{W5+$[OH]^{-}_{W4}$} (**Pr-I**)	9.6	-
**12^2**	**42.1**	Arg-287 Cγ	2.2	5.2			
		Arg-287 Cδ	4.6	5.6	Arg-287 Cδ	4.6	4.7
		His-323 Cε1-**I**	16.9	-	His-323 Cε1-**I**	16.9	-
					His-323 Nδ1-**I**	16.9	-
		His-323 Nε2-**I**	18.4	-	His-323 Nε2-**I**	18.4	-
					Val-337 Cβ	2.2	4.2
		Val-337 Cα	5.6	5.1	-	-	-
		Val-337 Cγ1	1.3	3.4			
					Val-337 Cγ2	1.3	2.7
		W4 (**Pr-II**)	8.9	-	W4 (**Pr-II**)	8.9	4.4
**13^1**	**10.3**	His-323 Cε1-**II**	12.1	4.9	His-323 Cε1-**II**	12.1	4.0
		His-323 Nδ1-**I**	13.6	-	-	-	-
		His-323 Nε2-**I**	18.4	-	His-323 Nε2-**I**	18.4	-
					Ile-273 Cα	4.3	5.3
		Leu-368 Cγ	2.3	5.4	Leu-368 Cγ	2.3	5.4
		Leu-368 Cδ1	1.7	5.7	Leu-368 Cδ1	1.7	3.2
		Val-370 Cα	4.4	5.9			
					Val-370 Cγ1	0.9	4.8
		Val-370 Cγ2	0.9	4.0			
		W6	7.7	-	-	-	-
**15**	**93.8**	Asp-272 Cβ	3.5	4.6	Asp-272 Cβ	3.5	3.6
		His-323 Cδ2-**I**	16.1	-	His-323 Cδ2-**I**	16.1	-
		His-323 Cε1-**I**	16.9	-			
					His-323 Nδ1-**I**	16.9	-
		His-323 Nε2-**II**	14.7	5.8	His-323 Nε2-**II**	14.7	5.7
		Ile-273 Cβ	2.9	5.0	Ile-273 Cβ	2.9	5.5
		Tyr-326 Cδ1	11.4	5.2	-	-	-
					Tyr-326 Cε1	11.8	5.8
		Tyr-326 Cε2	11.8	5.5			
		W1-**II**	7.9	-	W1-**II**	7.9	3.7
		W5 (**Pr-II**)	8.8	-	W5 (**Pr-II**)	8.8	-
**17^1**	**10.1**	Arg-552 Nη1	12.4	5.1			
					Arg-552 Nη2	12.4	5.8
		Ile-273 Cδ1	1.8	5.6	Ile-273 Cδ1	1.8	3.0
		Met-320 Cε	3.8	5.0			
		Tyr-241 Cε1	9.0	5.0	Tyr-241 Cε1	9.0	5.0
		Tyr-268 Cα	4.9	5.7	-	-	-
					Tyr-268 Cβ	3.8	3.4
					Tyr-268 Cδ1	11.4	4.5
		Tyr-326 Cδ1	11.4	5.4			
		Tyr-326 Oη	8.3	3.5	Tyr-326 Oη	8.3	4.0
		W7	7.2	-	-	-	-

		**Oat PhyA3–PCB Pr**					
		**Structural model for the native PΦB adduct**			**Structural model for the PCB adduct**		
cofactor carbon	δ^{C}_{Pr} (ppm)	¹H contact (residue)	δ^{H}_{Pr} (ppm)	HM distance (Å)	¹H contact (residue)	δ^{H}_{Pr} (ppm)	HM distance (Å)
**18[1]**	**16.7**	His-372 Nδ1 (**Pr-I**)	12.2	5.7			
					His-372 Nε2 (**Pr-II**)	12.2	5.3
		Met-239 Cε	3.1	4.6	Met-239 Cε	3.1	3.8
		Tyr-241 Cε1	9.0	5.2	Tyr-241 Cε1	9.0	4.5
		Tyr-263 Cδ2	8.9	5.3	-	-	-
		Tyr-263 Cβ	4.5	5.7	-	-	-
		Tyr-268 Oη	8.0	3.8	Tyr-268 Oη	8.0	5.1
		Tyr-326 Oη	8.3	4.4	Tyr-326 Oη	8.3	5.4
**18[2]**	**12.9**	His-372 Cδ2	12.1	5.5	-	-	-
		His-372 Cε1	10.4	3.7			
		His-372 Nε2 (**Pr-II**)	11.4	4.4			
		Met-239 Cγ	3.1	5.6			
					Met-239 Cε	3.1	3.5
		Met-320 Cγ	3.8	5.6			
					Tyr-241 Cδ1	10.4	3.0
					Tyr-241 Cε1	9.0	3.9
		Tyr-263 Cβ	4.5	4.9			
		Tyr-263 Cδ2	9.0	4.0			
					Tyr-268 Cβ	3.8	4.7
					Tyr-268 Cδ1	11.4	6.3
		Tyr-268 Oη	8.0	4.0	Tyr-268 Oη	8.0	5.9
					Val-251 Cα	4.5	5.8

Supplementary Table S12. Selected structural parameters of the PCB chromophore of the QM/MM models in both Pr and Pfr states for oat phyA3. The Pr structural data were extracted from the NMR-refined QM/MM model of oat phyA adducts with the PCB chromophore as Pr (deposited in Supplementary Material). The Pfr data were extracted from the corresponding Pfr model (also deposited in Supplementary Material). The methine bridge torsional angle represents the sum of the deviations of the dihedral angles from the ideal planar configuration and conformation, that is, 0° for *Z* and *syn* and 180° for *E* and *anti* (Mroginski et al., 2011).

bond length (Å) and dihedral angle (°)		Oat PhyA3–PCB Pr†	Oat PhyA3–PCB Pfr‡
ring A	**C1–C2**	1.519	1.519
	**C2–C3**	1.559	1.556
	**C3–C4**	1.535	1.538
	**C4=N21**	1.374	1.357
	**C1=N21**	1.402	1.392
	**N21–H21**	1.021	1.026
	**C1=O**	1.237	1.215
A–B bridge	**C4=C5**	1.371	1.371
	**C5–C6**	1.427	1.433
	**N21=C4–C5–C6**	–0.36	3.84
	**C4–C5–C6=N22**	13.22	2.64
ring B	**C6–C7**	1.441	1.440
	**C7–C8**	1.389	1.392
	**C8–C9**	1.444	1.434
	**C9=N22**	1.412	1.400
	**C6=N22**	1.378	1.368
	**N22–H22**	1.014	1.018
B–C bridge	**C9=C10**	1.386	1.392
	**C10=C11**	1.402	1.401
	**N22=C9–C10–C11**	2.85	9.63
	**C9–C10–C11=N23**	–7.58	10.97
ring C	**C11–C12**	1.427	1.431
	**C12–C13**	1.418	1.406
	**C13–C14**	1.420	1.415
	**C14=N23**	1.373	1.364
	**C11=N23**	1.393	1.384
	**N23–H23**	1.019	1.022
C–D bridge	**C14=C15**	1.447	1.447
	**C15=C16**	1.357	1.364
	**N23=C14–C15–C16**	–137.71	129.24
	**C14–C15–C16=N24**	11.88	–164.81
ring D	**C16–C17**	1.478	1.481
	**C17–C18**	1.364	1.362
	**C18–C19**	1.482	1.474
	**C19=N24**	1.394	1.374
	**C16=N24**	1.404	1.405
	**N24–H24**	1.006	1.011
	**C19=O**	1.249	1.235
methine bridge torsional angle (°)		**Pr†**	**Pfr‡**
	A–B	14.49	8.60
	B–C	13.05	20.07
	C–D	55.96	125.29

†NMR-refined Pr model based on Mroginski et al., 2011; ‡present work.

Supplementary Table S13. Amino acid conservation of *As*.phyA3 and *At*.phyB in the direct bilin environment. The sequence alignment was performed by PRALINE (Heringa,1999). The amino acid codes ranging from 1 to 627 for *As*.phyA3 (Song et al., 2012) follow the equivalents for *At*.phyB sequence (amino acid range of 89–632, Burgie et al., 2014). The scoring scheme ranges from for the least conserved alignment position to 10 for the most conserved alignment position 1 (conserved amino acids indicted by asterisks). "X" or "–" signify insertions / deletions or unresolved regions, respectively. Entries in color (green for *At*.phyB and red/blue for *As*.phyA3) indicate the amino acids in the close proximity to the respective bilin (for example, within a region of 6 Å). For *As*.phyA3, entries colored red and blue indicate the residues recognized in our previous $^{1}H^{residue}$–$^{13}C^{PCB}$ correlation experiments on this protein in its Pr state (Supplementary Table S9, see also Supplementary Figure S12), while blue for residues within the 6-Å sphere but not resolved in the NMR spectra (Supplementary Figures S2, S3).

At.phyB		*As*.phyA3			*At*.phyB		*As*.phyA3			*At*.phyB		*As*.phyA3			*At*.phyB		*As*.phyA3			*At*.phyB		*As*.phyA3			*At*.phyB		*As*.phyA3		
		1	MET		96	VAL	61	GLY	1	156	ILE	121	ARG	1	216	LEU	181	PHE	4	276	TYR	241	TYR	*	336	GLN	301	GLU	6
		2	SER		97	PRO	62	ARG	2	157	LEU	122	LEU	*	217	GLU	182	GLU	*	277	LYS	242	LYS	*	337	ASP	302	ALA	2
		3	SER		98	GLU	63	SER	4	158	ALA	123	GLY	4	218	PRO	183	PRO	*	278	PHE	243	PHE	*	338	ASP	303	GLU	6
		4	SER		99	GLN	64	GLU	6	159	MET	124	ILE	5	219	ALA	184	VAL	5	279	HIS	244	HIS	*	339	ARG	304	ALA	3
		5	ARG		100	GLN	65	LYS	5	160	GLY	125	GLY	*	220	ARG	185	LYS	6	280	GLU	245	GLU	*	340	LEU	305	LEU	*
		6	PRO		101	ILE	66	VAL	8	161	THR	126	THR	*	221	THR	186	PRO	3	281	ASP	246	ASP	*	341	THR	306	PRO	3
		7	ALA		102	THR	67	ILE	3	162	ASP	127	ASN	5	222	GLU	187	THR	3	282	GLU	247	ASP	6	342	GLN	307	PHE	1
		8	SER		103	ALA	68	ALA	*	163	VAL	128	VAL	*	223	ASP	188	GLU	6	283	HIS	248	HIS	*	343	SER	308	ASP	4
		9	SER		104	TYR	69	TYR	*	164	ARG	129	ARG	*	224	PRO	189	PHE	0	284	GLY	249	GLY	*	344	MET	309	ILE	5
		10	SER		105	LEU	70	LEU	*	165	SER	130	SER	*	225	ALA	190	PRO	3	285	GLU	250	GLU	*	345	CYS	310	SER	2
		11	SER		106	SER	71	GLN	4	166	LEU	131	LEU	*	226	LEU	191	ALA	3	286	VAL	251	VAL	*	346	LEU	311	LEU	*
		12	SER		107	ARG	72	HIS	3	167	PHE	132	PHE	*	227	SER	192	THR	5	287	VAL	252	PHE	3	347	VAL	312	CYS	2
		13	ARG		108	ILE	73	ILE	*	168	THR	133	SER	5	228	ILE	193	ALA	3	288	ALA	253	SER	6	348	GLY	313	GLY	*
		14	ASN		109	GLN	74	GLN	*	169	SER	134	ASP	4	229	ALA	194	ALA	*	289	GLU	254	GLU	*	349	SER	314	SER	*
		15	ARG		110	ARG	75	LYS	6	170	SER	135	GLN	4	230	GLY	195	GLY	*	290	SER	255	ILE	2	350	THR	315	ALA	4
		16	GLN		111	GLY	76	GLY	*	171	SER	136	GLY	4	231	ALA	196	ALA	*	291	LYS	256	THR	3	351	LEU	316	LEU	*
		17	SER		112	GLY	77	LYS	2	172	SER	137	ALA	6	232	VAL	197	LEU	6	292	ARG	257	LYS	6	352	ARG	317	ARG	*
		18	SER		113	TYR	78	LEU	3	173	ILE	138	THR	3	233	GLN	198	GLN	*	293	ASP	258	PRO	2	353	ALA	318	ALA	*
		19	GLN		114	ILE	79	ILE	*	174	LEU	139	ALA	3	234	SER	199	SER	*	294	ASP	259	GLY	3	354	PRO	319	PRO	*
		20	ALA		115	GLN	80	GLN	*	175	LEU	140	LEU	*	235	GLN	200	TYR	3	295	LEU	260	LEU	*	355	HIS	320	HIS	*
		21	ARG		116	PRO	81	THR	3	176	GLU	141	HIS	3	236	LYS	201	LYS	*	296	GLU	261	GLU	*	356	GLY	321	SER	4
		22	VAL		117	PHE	82	PHE	*	177	ARG	142	LYS	6	237	LEU	202	LEU	*	297	PRO	262	PRO	*	357	CYS	322	CYS	*
		23	LEU		118	GLY	83	GLY	*	178	ALA	143	ALA	*	238	ALA	203	ALA	*	298	TYR	263	TYR	*	358	HIS	323	HIS	*
		24	ALA		119	CYS	84	CYS	*	179	PHE	144	LEU	4	239	VAL	204	ALA	5	299	ILE	264	LEU	7	359	SER	324	LEU	2
		25	GLN		120	MET	85	LEU	7	180	VAL	145	GLY	1	240	ARG	205	LYS	6	300	GLY	265	GLY	*	360	GLN	325	GLN	*
		26	THR		121	ILE	86	LEU	7	181	ALA	146	PHE	2	241	ALA	206	ALA	*	301	LEU	266	LEU	*	361	TYR	326	TYR	*
		27	THR		122	ALA	87	ALA	*	182	ARG	147	ALA	3	242	ILE	207	ILE	*	302	HIS	267	HIS	*	362	MET	327	MET	*
		28	LEU		123	VAL	88	LEU	6	183	GLU	148	ASP	6	243	SER	208	SER	*	303	TYR	268	TYR	*	363	ALA	328	GLU	3
		29	ASP		124	ASP	89	ASP	*	184	ILE	149	VAL	8	244	GLN	209	LYS	5	304	PRO	269	PRO	*	364	ASN	329	ASN	*
		30	ALA		125	GLU	90	GLU	*	185	THR	150	SER	5	245	LEU	210	ILE	7	305	ALA	270	ALA	*	365	MET	330	MET	*
		31	GLU		126	SER	91	LYS	4	186	LEU	151	LEU	*	246	GLN	211	GLN	*	306	THR	271	THR	*	366	GLY	331	ASN	4
		32	LEU		127	SER	92	SER	*	187	LEU	152	LEU	*	247	ALA	212	SER	6	307	ASP	272	ASP	*	367	SER	332	SER	*
		33	ASN		128	PHE	93	PHE	*	188	ASN	153	ASN	*	248	LEU	213	LEU	*	308	ILE	273	ILE	*	368	ILE	333	ILE	*
		34	ALA		129	ARG	94	ASN	4	189	PRO	154	PRO	*	249	PRO	214	PRO	*	309	PRO	274	PRO	*	369	ALA	334	ALA	*
		35	GLU		130	ILE	95	VAL	8	190	VAL	155	ILE	8	250	GLY	215	GLY	*	310	GLN	275	GLN	*	370	SER	335	SER	*
		36	TYR		131	ILE	96	ILE	*	191	TRP	156	LEU	1	251	GLY	216	GLY	*	311	ALA	276	ALA	*	371	LEU	336	LEU	*
		37	GLU		132	GLY	97	ALA	4	192	ILE	157	VAL	8	252	ASP	217	SER	4	312	SER	277	ALA	6	372	ALA	337	VAL	5
		38	GLU		133	TYR	98	PHE	6	193	HIS	158	GLN	3	253	ILE	218	MET	5	313	ARG	278	ARG	*	373	MET	338	MET	*
		39	SER		134	SER	99	SER	*	194	SER	159	CYS	2	254	LYS	219	GLU	5	314	PHE	279	LEU	4	374	ALA	339	ALA	*
		40	GLY		135	GLU	100	GLU	*	195	LYS	160	LYS	*	255	LEU	220	VAL	6	315	LEU	280	LEU	*	375	VAL	340	VAL	*
		41	ASP		136	ASN	101	ASN	*	196	ASN	161	THR	4	256	LEU	221	LEU	*	316	PHE	281	PHE	*	376	ILE	341	VAL	8
		42	SER		137	ALA	102	ALA	*	197	THR	162	SER	5	257	CYS	222	CYS	*	317	LYS	282	MET	3	377	ILE	342	VAL	8
		43	PHE		138	ARG	103	PRO	2	198	GLY	163	GLY	*	258	ASP	223	ASN	5	318	GLN	283	LYS	5	378	ASN	343	ASN	*
		44	ASP		139	GLU	104	GLU	*	199	LYS	164	LYS	*	259	THR	224	THR	*	319	ASN	284	ASN	*	379	GLY	344	GLU	2
		45	TYR		140	MET	105	MET	*	200	PRO	165	PRO	*	260	VAL	225	VAL	*	320	ARG	285	LYS	6	380	ASN	345	ASN	*
		46	SER		141	LEU	106	LEU	*	201	PHE	166	PHE	*	261	VAL	226	VAL	*	321	VAL	286	VAL	*	381	GLU	346	GLU	*
		47	LYS		142	GLY	107	THR	2	202	TYR	167	TYR	*	262	GLU	227	LYS	5	322	ARG	287	ARG	*	382	ASP	347	GLU	6
		48	LEU		143	ILE	108	THR	3	203	ALA	168	ALA	*	263	SER	228	GLU	4	323	MET	288	MET	*	383	ASP	348	ASP	*
89	MET	49	VAL	5	144	MET	109	VAL	5	204	ILE	169	ILE	*	264	VAL	229	VAL	*	324	ILE	289	ILE	*	384	GLY	349	ASP	3
90	THR	50	GLU	3	145	PRO	110	SER	3	205	LEU	170	VAL	6	265	ARG	230	PHE	1	325	VAL	290	CYS	2	385	SER	350	GLU	4
91	THR	51	ALA	4	146	GLN	111	HIS	3	206	HIS	171	HIS	*	266	ASP	231	ASP	*	326	ASP	291	ASP	*	386	ASN	351	ALA	2
92	TYR	52	GLN	2	147	SER	112	ALA	6	207	ARG	172	ARG	*	267	LEU	232	LEU	*	327	CYS	292	CYS	*	-	-	352	GLU	0
93	GLY	53	ARG	3	148	VAL	113	VAL	*	208	ILE	173	ALA	3	268	THR	233	THR	*	328	ASN	293	ARG	4	-	-	353	SER	0
-	-	54	ASP	0	149	PRO	114	PRO	*	209	ASP	174	THR	3	269	GLY	234	GLY	*	329	ALA	294	ALA	*	-	-	354	GLU	0
-	-	55	GLY	0	150	THR	115	SER	5	210	VAL	175	GLY	1	270	TYR	235	TYR	*	330	THR	295	ARG	3	-	-	355	GLN	0
-	-	56	PRO	0	151	LEU	116	VAL	6	211	GLY	176	CYS	0	271	ASP	236	ASP	*	331	PRO	296	SER	3	387	VAL	356	PRO	2
-	-	57	PRO	0	152	GLU	117	ASP	6	212	VAL	177	LEU	6	272	ARG	237	ARG	*	332	VAL	297	ILE	8	388	ALA	357	ALA	*
-	-	58	VAL	0	153	LYS	118	ASP	3	213	VAL	178	VAL	*	273	VAL	238	VAL	*	333	LEU	298	LYS	2	389	SER	358	GLN	4
94	SER	59	GLN	4	154	PRO	119	PRO	*	214	ILE	179	VAL	8	274	MET	239	MET	*	334	VAL	299	VAL	*	390	GLY	359	GLN	2
95	SER	60	GLN	4	155	GLU	120	PRO	3	215	ASP	180	ASP	*	275	VAL	240	ALA	5	335	VAL	300	ILE	8	391	ARG	360	GLN	5

At.phyB		*As*.phyA3			*At*.phyB		*As*.phyA3			*At*.phyB		*As*.phyA3			*At*.phyB		*As*.phyA3			*At*.phyB		*As*.phyA3			*At*.phyB		*As*.phyA3		
392	SER	361	LYS	4	442	LYS	411	LYS	*	491	GLY	461	ARG	2	541	VAL	511	VAL	*	591	VAL	561	VAL	*	-	-	611	GLY	0
393	SER	362	LYS	4	443	ARG	412	ASN	4	492	VAL	462	ASN	1	542	ALA	512	ALA	*	592	VAL	562	VAL	*	-	-	612	LEU	0
394	MET	363	LYS	3	444	VAL	413	ILE	8	493	ALA	463	ALA	*	543	TYR	513	LYS	2	593	LYS	563	LYS	*	-	-	613	ALA	0
395	ARG	364	LYS	6	445	LEU	414	LEU	*	494	PRO	464	PRO	*	544	ILE	514	ILE	*	594	SER	564	MET	3	-	-	614	GLU	0
396	LEU	365	LEU	*	446	ARG	415	LYS	6	495	SER	465	THR	5	545	THR	515	ASN	4	595	ARG	565	LYS	6	-	-	615	LEU	0
397	TRP	366	TRP	*	447	THR	416	MET	3	496	X	466	GLU	6	546	LYS	516	SER	4	596	SER	566	SER	*	-	-	616	GLN	0
398	GLY	367	GLY	*	448	GLN	417	GLN	*	497	X	467	SER	7	547	ARG	517	LYS	6	597	GLN	567	LEU	2	-	-	617	ALA	0
399	LEU	368	LEU	*	449	THR	418	THR	*	498	X	468	GLN	6	548	ASP	518	ASP	*	598	PRO	568	PRO	*	-	-	618	VAL	0
400	VAL	369	LEU	6	450	LEU	419	MET	7	499	X	469	ILE	7	549	PHE	519	ILE	4	599	TRP	569	TRP	*	-	-	619	THR	0
401	VAL	370	VAL	*	451	LEU	420	LEU	*	500	X	470	HIS	5	550	LEU	520	LEU	*	600	GLU	570	SER	4	-	-	620	SER	0
402	CYS	371	CYS	*	452	CYS	421	SER	2	501	X	471	ASP	6	551	PHE	521	PHE	*	601	THR	571	ASP	3	-	-	621	GLU	0
403	HIS	372	HIS	*	453	ASP	422	ASP	*	502	X	472	ILE	7	552	TRP	522	TRP	*	602	ALA	572	TYR	2	-	-	622	MET	0
404	HIS	373	HIS	*	454	MET	423	MET	*	503	X	473	ALA	7	553	PHE	523	PHE	*	603	GLU	573	GLU	*	-	-	623	VAL	0
405	THR	374	GLU	3	455	LEU	424	LEU	*	504	X	474	PHE	6	554	ARG	524	ARG	*	604	MET	574	MET	*	-	-	624	ARG	0
406	SER	375	SER	*	456	LEU	425	PHE	4	505	X	475	TRP	5	555	SER	525	SER	*	605	ASP	575	ASP	*	-	-	625	LEU	0
407	SER	376	PRO	3	457	ARG	426	ARG	*	506	X	476	LEU	7	556	HIS	526	HIS	*	606	ALA	576	ALA	*	-	-	626	MET	0
408	ARG	377	ARG	*	458	ASP	427	GLU	6	507	X	477	SER	7	557	THR	527	THR	*	607	ILE	577	ILE	*	-	-	627	GLU	0
409	CYS	378	TYR	1	459	SER	428	ALA	6	508	X	478	ASP	6	558	ALA	528	ALA	*	608	HIS	578	HIS	*					
410	ILE	379	VAL	8	-	-	429	SER	0	509	ASN	479	VAL	1	559	LYS	529	ALA	3	609	SER	579	SER	*					
411	PRO	380	PRO	*	460	PRO	430	PRO	*	510	HIS	480	HIS	*	560	GLU	530	GLU	*	610	LEU	580	LEU	*					
412	PHE	381	PHE	*	461	ALA	431	LEU	3	511	ALA	481	ARG	3	561	ILE	531	ILE	*	611	GLN	581	GLN	*					
413	PRO	382	PRO	*	462	GLY	432	THR	2	512	ASP	482	ASP	*	562	LYS	532	ARG	6	612	LEU	582	LEU	*					
414	LEU	383	LEU	*	463	ILE	433	ILE	*	513	SER	483	SER	*	563	TRP	533	TRP	*	613	ILE	583	ILE	*					
415	ARG	384	ARG	*	464	VAL	434	VAL	*	514	THR	484	THR	*	564	GLY	534	GLY	*	614	LEU	584	LEU	*					
416	TYR	385	TYR	*	465	THR	435	SER	5	515	GLY	485	GLY	*	565	GLY	535	GLY	*	615	ARG	585	ARG	*					
417	ALA	386	ALA	*	466	GLN	436	GLY	2	516	LEU	486	LEU	*	566	ALA	536	ALA	*	616	ASP	586	GLY	3					
418	CYS	387	CYS	*	467	SER	437	THR	5	517	SER	487	SER	*	567	LYS	537	LYS	*	617	SER	587	THR	5					
419	GLU	388	GLU	*	468	PRO	438	PRO	*	518	THR	488	THR	*	568	HIS	538	ASN	4	618	PHE	588	LEU	4					
420	PHE	389	PHE	*	469	SER	439	ASN	5	519	ASP	489	ASP	*	569	HIS	539	ASP	2	619	LYS	589	ASN	4					
421	LEU	390	LEU	*	470	ILE	440	ILE	*	520	SER	490	SER	*	570	PRO	540	PRO	*	620	GLU	590	ASP	6					
422	MET	391	ALA	3	471	MET	441	MET	*	521	LEU	491	LEU	*	571	GLU	541	SER	4	621	SER	591	ALA	6					
423	GLN	392	GLN	*	472	ASP	442	ASP	*	522	GLY	492	HIS	1	572	ASP	542	ASP	*	622	GLU	592	SER	4					
424	ALA	393	VAL	3	473	LEU	443	LEU	*	523	ASP	493	ASP	*	573	LYS	543	MET	3	623	ALA	593	LYS	3					
425	PHE	394	PHE	*	474	VAL	444	VAL	*	524	ALA	494	ALA	*	574	ASP	544	ASP	*	624	ALA	594	PRO	3					
426	GLY	395	ALA	4	475	LYS	445	LYS	*	525	X	495	GLY	6	575	ASP	545	ASP	*	625	SER	595	LYS	4					
427	LEU	396	VAL	6	476	CYS	446	CYS	*	526	X	496	TYR	6	576	GLY	546	SER	4	626	LEU	596	ARG	2					
428	GLN	397	HIS	3	477	ASP	447	ASP	*	527	X	497	PRO	6	577	GLN	547	ARG	5	627	HIS	597	GLU	3					
429	LEU	398	VAL	6	478	GLY	448	GLY	*	528	X	498	GLY	6	578	ARG	548	ARG	*	628	HIS	598	ALA	2					
430	ASN	399	ASN	*	479	ALA	449	ALA	*	529	X	499	ALA	7	579	MET	549	MET	*	629	HIS	599	SER	3					
431	MET	400	ARG	3	480	ALA	450	ALA	*	530	X	500	ALA	7	580	HIS	550	HIS	*	630	HIS	600	LEU	5					
432	GLU	401	GLU	*	481	PHE	451	LEU	4	531	ALA	501	ALA	*	581	PRO	551	PRO	*	631	HIS	601	ASP	1					
433	LEU	402	PHE	4	482	LEU	452	LEU	*	532	LEU	502	LEU	*	582	ARG	552	ARG	*	632	HIS	602	ASN	2					
434	GLN	403	GLU	6	483	TYR	453	TYR	*	533	GLY	503	GLY	*	583	SER	553	LEU	2	-	-	603	GLN	0					
435	LEU	404	LEU	*	484	HIS	454	GLY	1	534	ASP	504	ASP	*	584	SER	554	SER	*	-	-	604	ILE	0					
436	ALA	405	GLU	3	485	GLY	455	GLY	*	535	ALA	505	MET	3	585	PHE	555	PHE	*	-	-	605	GLY	0					
437	LEU	406	LYS	2	486	LYS	456	LYS	*	536	VAL	506	ILE	8	586	GLN	556	LYS	5	-	-	606	ASP	0					
438	GLN	407	GLN	*	487	TYR	457	VAL	3	537	CYS	507	CYS	*	587	ALA	557	ALA	*	-	-	607	LEU	0					
439	MET	408	LEU	7	488	TYR	458	TRP	4	538	GLY	508	GLY	*	588	PHE	558	PHE	*	-	-	608	LYS	0					
440	SER	409	ARG	3	489	PRO	459	ARG	2	539	MET	509	MET	*	589	LEU	559	LEU	*	-	-	609	LEU	0					
441	GLU	410	GLU	*	490	LEU	460	LEU	*	540	ALA	510	ALA	*	590	GLU	560	GLU	*	-	-	610	ASP	0					
